# Assessing Health-Related Quality of life and socioeconomic impacts in low-endemic schistosomiasis: A study from Rural China

**DOI:** 10.1371/journal.pntd.0014544

**Published:** 2026-07-16

**Authors:** Fei Hu, Jun Ge, Yifeng Li, Zhihong Gong, Huiqun Xie, Jun Wu, Zhuo Wang, Sheng Ding

**Affiliations:** 1 Jiangxi Provincial Institute of Parasitic Diseases, Nanchang, Jiangxi, P.R. China; 2 Department of Geriatrics, The Affiliated Wuxi People’s Hospital of Nanjing Medical University, Wuxi, Jiangsu, P.R. China; Jiangsu Institute of Parasitic Diseases, CHINA

## Abstract

To describe and compare health-related quality of life (HRQL) across disease stages (advanced schistosomiasis [AS], schistosomiasis-induced liver fibrosis [SLF], and healthy controls [HP]) in a low-endemic setting, and to explore associations between HRQL and socioeconomic/geographic factors. The findings aim to inform targeted interventions and policies at specific disease stages. A cross-sectional study was conducted in a schistosomiasis-endemic county in Jiangxi Province, China. A total of 2,652 participants, including 884 each in the AS, SLF, and healthy population (HP) groups, were enrolled. HRQL was assessed using the validated Chinese version of the SF-36 questionnaire. Socioeconomic variables (per-capita disposable income of rural households [P-DIRH], per-capita gross domestic product [P-GDP]) and geographic variables (type of schistosomiasis endemic area, endemic status) were analyzed. Non-parametric tests and multivariate linear regression (SPSS 27.0) were employed for statistical analyses. It was observed that the HRQL scores showed a gradually increasing trend (AS <SLF < HP) for the physical component summary (PCS). The median PCS and mental component summary (MCS) scores were lowest in the AS group (71.30 and 71.95), significantly lower than those in the SLF group (78.80 and 77.20) and the HP group (83.80 and 80.80). Patients with AS exhibited the most severe impairments in physical functioning (PF), vitality (VT), social function (SF), and general health (GH). Age ≥ 60 years was most strongly associated with lower HRQL scores (PCS: *β* = -0.217 to -0.266; MCS: *β* = -0.153 to -0.168). Areas meeting elimination criteria were positively associated with HRQL (*β* = 0.114 to 0.109), while low P-GDP regions were strongly associated with poorer HRQL (P < 0.05). HRQL is significantly impaired among schistosomiasis patients in low-endemic areas, driven by age, economic status, and regional disease control efforts. This study underscores the need for integrated interventions combining biomedical care, mental health support, and economic policies to alleviate the long-term burden of schistosomiasis and advance health equity, aligning with the World Health Organization’s 2030 elimination goals.

## Author summary

In areas where schistosomiasis is nearly eliminated (“low-endemic”), the long-term health and socioeconomic impacts on patients with chronic complications like Advanced Schistosomiasis (AS) and Schistosomiasis-induced Liver Fibrosis (SLF) remain poorly understood. These patients often face irreversible disabilities, yet most research focuses on high-transmission settings. We assessed health-related quality of life (HRQL) in 2,652 participants across rural China (884 AS, 884 SLF, and 884 healthy individuals) using validated surveys. HRQL showed a steep decline: AS patients scored lowest (median physical/mental scores: 71.3/71.95), followed by SLF (78.8/77.2), and healthy groups (83.8/80.8). Age ≥ 60 years was the strongest factor associated with lower HRQL. Economically disadvantaged areas (low GDP) and regions not meeting elimination targets had significantly poorer outcomes. Physical function, vitality, and social health were most severely impaired in AS patients. Even in low-transmission zones, chronic schistosomiasis profoundly reduces quality of life, driven by aging, economic inequality, and regional disease control gaps. Interventions must integrate medical care, mental health support, and economic policies—especially for elderly patients in resource-limited areas—to align with WHO elimination goals. This study provides stage-specific, equity-focused strategies to alleviate the enduring burden of schistosomiasis.

## Introduction

Schistosomiasis, a neglected tropical disease caused by Schistosoma spp., remains a major global health burden, with an estimated 251.4 million individuals requiring annual preventive treatment [1]. While global control efforts have significantly reduced disease prevalence, the long-term consequences on patients’ health-related quality of life (HRQL) persist, particularly in low-prevalence settings where the health burden of residual cases may persist as a result of prolonged periods of inadequate treatment [2].

Over the past few decades, China has significantly reduced schistosomiasis transmission risk through integrated control strategies [3], with all endemic areas achieving transmission interruption or elimination [4]. Although the ecological supporting pathogen transmission (e.g., presence of infected snails or contaminated water bodies) still exist in some areas, the effective implementation of control measures has led to a marked decline in population-level infection rates. However, even in low-transmission settings, advanced schistosomiasis (AS) and schistosomiasis-induced liver fibrosis (SLF) continue to affect previously infected individuals, leading to irreversible organ damage, disability, and severe HRQL impairment [5]. These chronic conditions pose ongoing threats to affected populations, underscoring the necessity of long-term disease management and HRQL assessment in post-control settings. In alignment with this need, the World Health Organization (WHO) emphasizes addressing the enduring burden of schistosomiasis to ensure sustainable elimination [6].

While existing studies have prioritized epidemiological surveillance and reducing the incidence of presenting infections in high-transmission settings, research on HRQL in low-prevalence areas—particularly among individuals with SLF—remains limited, resulting in their underrepresentation. AS and SLF are associated with physical disability, psychological distress, and socioeconomic challenges [7]. In schistosomiasis-endemic regions, these conditions frequently co-occur with aging populations, exacerbating the disease burden [2]. Furthermore, socioeconomic disparities in rural areas exacerbate health disparities in affected populations [8]. These gaps hinder the development of targeted interventions for residual infections—i.e., chronic, long-standing schistosomiasis cases (including AS and SLF) that persist even after transmission has been largely interrupted—because such patients are often excluded from routine surveillance focused on new infections.

Over the past two decades, HRQL assessment in schistosomiasis has garnered growing attention, driven by the recognition that chronic infection profoundly affects physical, psychological, and social well-being. For instance, King et al. conducted a systematic evaluation of the long-term consequences of schistosomiasis, specifically the hepatosplenic form, on quality of life [2]. Similarly, Roriz et al. reported severe impairments across all HRQL dimensions in Brazilian patients with hepatosplenic Schistosomiasis mansoni [9], a pattern corroborated in other countries among individuals with schistosomiasis-associated liver disease [10]. These collective findings highlight the value of generic HRQL instruments in comprehensively assessing multidimensional health impacts [11].

In China, HRQL in schistosomiasis have predominantly focused on presenting infection or isolated disease stages. For instance, an HRQL assessment of patients with diverse schistosomiasis subtypes in Hubei Province revealed significant disparities in quality of life scores across four dimensions: somatic role functioning, general health, emotional role functioning, and psychological well-being [12]. Among patients diagnosed with advanced schistosomiasis (AS), over 90% reported impairments in at least one health domain, including pain or discomfort (90.7%), daily activities (87.9%), and anxiety or depression (80.9%) [13]. However, the interplay between comorbidities—particularly irreversible liver damage in AS and schistosomiasis-induced liver fibrosis (SLF)—remains poorly understood. Notably, Ning et al. identified liver function abnormalities in an AS/SLF cohort but did not correlate these findings with HRQL outcomes [14]. Additionally, although Jia et al. showed that poverty exacerbates chronic schistosomiasis-related disability in low-income communities [15], socio-environmental correlates (e.g., regional heterogeneity in disease prevalence) are seldom incorporated into analyses.

Despite some progress in schistosomiasis-related quality of life research, certain gaps remain. Most studies continue to prioritize high-transmission areas, where disease dynamics and healthcare infrastructure differ substantially from low-transmission settings. Furthermore, existing research predominantly focuses on AS patients, with limited stratification by disease severity (e.g., AS vs. SLF). This oversight obscures the necessity for stage-specific interventions. Additionally, socioeconomic correlates—such as income disparities and regional heterogeneity—are frequently neglected, undermining comprehensive understanding of HRQL disparities.

The MOS-item Short Form Health Survey (SF-36) is widely recognized as a universal quality-of-life assessment tool with high reliability and validity [16]. Previous studies have demonstrated its utility in population health monitoring, disease severity estimation, clinical outcome evaluation, and treatment effectiveness assessment [17]. However, the application of survey scales for HRQL assessment requires rigorous validation across diverse populations, as cultural, religious, educational, and contextual factors may influence their performance [18]. In China, the reliability and validity of the SF-36 have been specifically evaluated in schistosomiasis patient populations [19]. Standardized global use of the SF-36 facilitates cross-regional comparisons, aligning with the World Health Organization’s recommendation to integrate HRQL metrics into neglected tropical disease programs [20].

In China, populations at risk of schistosomiasis are predominantly rural. Therefore, this study was conducted in rural areas of Jiangxi Province, where AS cases are concentrated. Jiangxi Province has historically been one of the regions most severely affected by schistosomiasis japonica, with an estimated 5,229,400 individuals at risk [4]; alongside 17,575 registered cases of schistosome-related liver damage [21]. Against this backdrop, this study aimed to assess HRQL of schistosomiasis patients in Jiangxi’s low-transmission setting and identify factors associated with it. The findings provide a scientific basis for optimizing disease prevention strategies and improving patient-centered care.

This study provides a stratified analysis of HRQL distinguishing between AS and SLF in a low-endemic setting and makes three key contributions: (i) Stage-specific insights: By distinguishing between AS and SLF cohorts, we identified distinct HRQL profiles, providing a foundation for stage-targeted rehabilitation interventions. (ii) Socioeconomic integration: By integrating regional economic development data with schistosomiasis prevalence, we identified associations suggesting an interplay between structural inequalities and health outcomes, advocating for multisectoral policy interventions. (iii) Methodological rigor: The application of the SF-36 scale ensured cross-study comparability and facilitated evidence-based policy discussions. These findings align with the World Health Organization’s 2030 Roadmap for Neglected Tropical Diseases [22], which prioritizes holistic rehabilitation and equitable care in eradication efforts. Combining epidemiological and quality-of-life approaches, this study promotes a paradigm shift—from morbidity reduction to comprehensive health restoration—in schistosomiasis control strategies.

This study employed a descriptive and exploratory approach to compare HRQL across disease stages and to identify factors associated with HRQL outcomes, without implying causal mechanisms.

## Results

### Sample characteristics

The study included 2,652 participants, equally stratified into AS, SLF, and HP groups based on predefined criteria. Among them, 1,566 (59.05%) were male. The age range was 22–75 years, with a mean age of 64.43 ± 5.42 years; 77.15% of participants were aged ≥60 years. A total of 1,803 participants (67.99%) resided in areas where P-DIRH exceeded the provincial average. However, a higher proportion (77.83%) lived in regions with P-GDP below the provincial average. Geographically, 41.29% of participants inhabited marshland/lake regions, whereas 72.06% were from areas meeting schistosomiasis transmission interruption criteria.

### Overall assessment of quality of life

**Fig 1:**
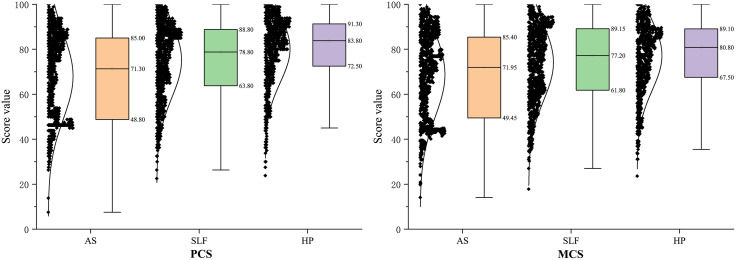
Distribution of scores for the physical component summary (PCS) and the mental component summary (MCS) across population groups (AS: advanced schistosomiasis; SLF: schistosomiasis-induced liver fibrosis; HP: healthy population. ).

The median PCS and MCS scores were 71.30 (48.80, 85.00) and 71.95 (49.45, 85.40) in the AS group, 78.80 (63.80, 88.80) and 77.20 (61.80, 89.15) in the SLF group, and 83.80 (72.50, 91.30) and 80.80 (67.50, 89.10) in the HP group (Fig 1). Both PCS and MCS scores differed significantly across the three groups (*Η* = 187.639 and 90.125, *all* P < 0.001). Pairwise comparisons revealed that PCS and MCS scores were significantly lower in the AS group than in the SLF and HP groups (*adj.* P < 0.001). Furthermore, the SLF group exhibited significantly lower PCS scores than the HP group (*adj.* P < 0.001). These results show lower HRQL scores in the SLF group compared to the HP group, and lowest scores in the AS group among the three groups.

### Comparison of dimension scores

**Fig 2:**
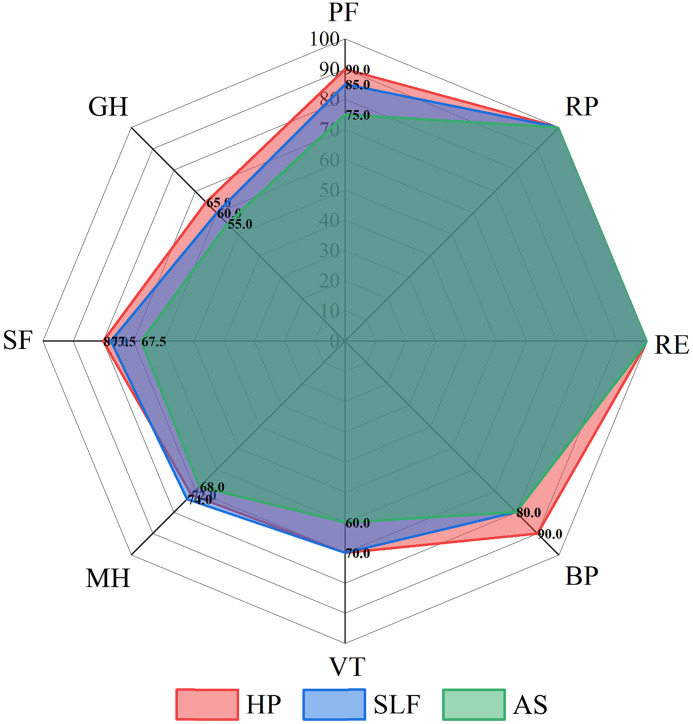
Medians for different populations for each dimension of the SF-36 scale (AS: advanced schistosomiasis; SLF: schistosomiasis-induced liver fibrosis; HP: healthy population. PF: physical functioning; RP: role- physical; RE: role-emotional; BP: bodily pain; VT: vitality; MH: mental health; SF: social function; GH: general health.).

The median scores for the eight SF-36 dimensions varied significantly across groups (Fig 2). In the AS group, scores were PF: 75.0 (60.0, 90.0), RP: 100 (0, 100), RE: 100 (0, 100), BP: 80.0 (80.0, 100), VT: 60.0 (50.0, 80.0), MH: 68.0 (60.0, 80.0), SF: 67.5 (65.0, 90.0), and GH: 55.0 (45.0, 70.0). For the SLF group, scores were PF: 85.0 (65.0, 95.0), RP: 100 (75.0, 100), RE: 100 (66.67, 100), BP: 80.0 (80.0, 100), VT: 70.0 (50.0, 80.0), MH: 74.0 (56.0, 84.0), SF: 77.5 (57.5, 100), and GH: 60.0 (50.0, 70.0). In contrast, the HP group demonstrated higher median scores: PF: 90.0 (75.0, 98.75), RP: 100 (100, 100), RE: 100 (100, 100), BP: 90.0 (80.0, 100), VT: 70.0 (50.0, 85.0), MH: 72.0 (60.0 84.0), SF: 80.0 (67.5, 100), and GH: 65.0 (50.0, 75.0). Tests revealed significant differences (*H* = 119.184[PF], 48.037[BP], 42.505[VT], 102.000[MH], 80.883[SF], 107.434[GH]; *all P* < 0.05) in all dimensions except RP and RE. Pairwise comparisons showed that PF, VT, SF, and GH scores were significantly lower in the AS group than in both the SLF and HP groups (*adj. P* < 0.001). Additionally, BP scores were significantly lower in the AS group compared to the HP group (*adj. P* < 0.001), while MH scores were significantly lower in the AS group than in the SLF group (*adj. P* < 0.05). In the SLF group, PF, BP, SF, and GH scores remained significantly lower than those in the HP group (*adj. P* < 0.001).

### Analysis of factors affecting HRQL in schistosomiasis patients

Multiple linear regression (enter regression) analyses revealed distinct correlates of HRQL across groups (Table 1). In the AS group, gender (female: *β* = -0.097, *P* < 0.001), age ≥ 60 years (*β* = -0.223, *P* < 0.001), low P-GDP (*β* = -0.102, *P* < 0.001), and elimination-status areas (*β* = 0.122, *P* < 0.001) were significantly associated with PCS scores. For MCS scores, gender (female: *β* = -0.093, *P* < 0.001), age ≥ 60 years (*β* = -0.147, *P* < 0.001), low P-DIRH (*β* = -0.067, *P* = 0.023), hilly/mountainous regions (*β* = -0.118, *P* < 0.001), and elimination status (*β* = 0.110, *P* < 0.001) were key predictors. Age ≥ 60 years showed the largest negative standardized coefficients on both PCS (*β* = -0.223) and MCS (*β* = -0.147) in AS patients. Elimination status was associated with higher PCS (*β* = 0.122) and MCS (*β* = 0.110) scores in this group.

**Table 1:** Factors associated with SF-36 scores in a population of schistosomiasis patients (multivariable linear regression results).

Overall rating category	Significant Predictors	AS			SLF		
		*β*		*p*	*β*		*p*
		Value	*95% CI*		Value	*95% CI*	
PCS	Gender(Female)	-0.097	-5.236 ~ -1.910	<0.001	-0.112	-5.107 ~ -2.186	<0.001
	Age(60–75)	-0.223	-11.722 ~ -7.600	<0.001	-0.263	-11.890 ~ -8.269	<0.001
	P-DIRH(Low)	-0.048	-4.088 ~ 0.348	0.098	-0.020	-2.638 ~ 1.256	0.486
	P-GDP(Low)	-0.102	-6.830 ~ -2.142	<0.001	-0.035	-3.407 ~ 0.714	0.200
	TSE(Hilly/mountainous regions)	-0.019	-2.876 ~ 1.498	0.537	0.067	2.261 ~ 4.101	0.026
	ESS(Elimination)	0.122	2.777 ~ 7.115	<0.001	-0.033	-3.074 ~ 0.735	0.229
	Sample size (N)	884			884		
	Model fit (R²/adjusted R²)	0.079/0.076			0.089/0.086		
MCS	Gender(Female)	-0.093	-5.027 ~ -1.721	<0.001	-0.122	-5.539 ~ -2.548	<0.001
	Age(60–75)	-0.147	-8.268 ~ -4.170	<0.001	-0.170	-8.423 ~ -4.715	<0.001
	P-DIRH(Low)	-0.067	-4.758 ~ -0.348	0.023	-0.084	-4.906 ~ -0.919	0.004
	P-GDP(Low)	-0.031	-3.671 ~ 0.989	0.259	0.011	-1.685 ~ 2.534	0.693
	TSE(Hilly/mountainous regions)	-0.118	-6.434 ~ -2.086	<0.001	-0.070	-4.263 ~ -0.331	0.022
	ESS(Elimination)	0.110	2.178 ~ 6.490	<0.001	-0.057	-4.108 ~ -0.118	0.038
	Sample size (N)	884			884		
	Model fit (R²/adjusted R²)	0.043/0.040			0.064/0.061		

*β*: Standard Beta Coefficient; *95%CI*: 95% confidence interval for the unstandardized coefficient; AS: advanced schistosomiasis; SLF: schistosomiasis-induced liver fibrosis; PCS: physical component summary; MCS: mental component summary; P-DIRH: Per-capita disposable income of rural households; P-GDP: Per-capita gross domestic product; TSE: type of schistosomiasis endemic area; ESS: Endemic status of schistosomiasis.

Reference categories for categorical variables: Gender: Male; Age: 20–59 years; P-DIRH: High; P-GDP: High; TSE: Marshland/lake regions; ESS: Transmission interruption

In the SLF group, PCS scores were associated with gender (female: *β* = -0.112, *P* < 0.001), age ≥ 60 years (*β* = -0.263, *P* < 0.001), and hilly/mountainous regions (*β* = 0.067, *P* = 0.026). MCS scores were associated by gender (female: *β* = -0.122, *P* < 0.001), age ≥ 60 years (*β* = -0.170, *P* < 0.001), low P-DIRH (*β* = -0.084, *P* = 0.004), hilly/mountainous regions (*β* = -0.070, *P* = 0.022), and elimination status (*β* = -0.057, *P* = 0.038). Age ≥ 60 years showed the largest negative standardized coefficients for PCS (*β* = -0.263) and MCS (*β* = -0.170) in the SLF group. Regression diagnostics (multicollinearity, homoscedasticity, and residual normality) confirmed that the linear models met key assumptions (see Methods for details).

## Discussion

Given the cross-sectional design, the observed associations should be interpreted as correlational and do not imply causation.

This study provides two principal findings regarding HRQL in low-endemic schistosomiasis. First, HRQL scores followed a stepwise decline from healthy controls to SLF to AS patients for physical health (PCS), scores followed a stepwise decline (HP > SLF> AS); for mental health (MCS), AS patients showed significantly lower scores than both SLF and HP groups, while no significant difference was observed between SLF and HP, with AS patients showing the most severe impairments in physical functioning, vitality, social function, and general health. Second, age ≥ 60 years was the strongest factor associated with lower HRQL, outweighing the impact of economic indicators. Additionally, an exploratory observation was that residing in areas that had achieved official elimination status was positively associated with HRQL among AS patients, although this association was not consistently observed across disease stages (it was in the opposite direction in the SLF group) and should be interpreted with caution given potential confounding.

Our cross-sectional survey included 2,652 participants. The result showed differences in HRQL scores among those with AS, SLF and HP, indicating severely impaired quality of life in AS and SLF patients. This highlights the impact of schistosomiasis of varying degrees on physical and mental health (Fig 1). The median PCS and MCS scores for AS patients were 71.30 and 71.95, respectively, significantly lower than those of the SLF group (78.80 and 77.20) and the HP group (83.80 and 80.80). These findings underscore a progressive decline in physical function with disease severity, consistent with the pathophysiology of AS-associated liver fibrosis and portal hypertension [7]. Our results align with the work of Jia et al. (2011), who reported that severe *Schistosoma japonicum* infection correlates with increased disability weight and reduced functional capacity [13]. These observations are further supported by global evidence. For instance, studies in Brazil documented severe HRQL impairments in patients with hepatosplenic schistosomiasis [9], particularly among those with complications such as upper gastrointestinal bleeding, which exacerbate physical limitations and psychological distress [10]. Although an overall trend was observed (AS <SLF < HP), it was noted that the magnitude of MCS differences and statistical significance in the comparison between the SLF and HP groups may be weaker than that of PCS. This suggests that the manifestation of mental health impacts may not be entirely parallel to physical health impacts across different disease stages, which warrants further investigation in future studies.

Notably, the AS group exhibited significant deficits in the PF, VT, SF, and GH dimensions, likely attributable to severe schistosomiasis complications such as portal hypertension, ascites, and anemia—hallmarks of advanced disease [13,14]. In contrast, SLF patients demonstrated moderate impairment, reflecting subclinical progression of liver fibrosis, a precursor to advanced hepatosplenomegaly. This observation aligns with findings from Nascimento et al. (2018), who reported that even non-advanced fibrosis significantly reduces HRQL due to persistent pain and fatigue [23].

Multiple regression analyses identified age ≥ 60 years as the primary factor associated with lower HRQL. Older age exerted the strongest negative association on both physical component summary (PCS) (*β*_AS_=-0.223, *β*_SLF_ = -0.263) and mental component summary (MCS) scores (*β*_AS_=-0.147, *β*_SLF_ = -0.170), aligning with global evidence on aging and chronic disease comorbidity [2,17]. For example, King et al. (2005) highlighted that in schistosomiasis-endemic areas, ageing populations face a compounded burden of cumulative organ damage and limited access to healthcare [2].

Patients living in counties with lower overall economic development levels have lower HRQL scores compared to those living in counties with higher development levels. For instance, AS and SLF patients residing in counties categorized as “Low P-GDP” had significantly lower PCS scores compared to those in “High P-GDP” counties (*β*_AS_ = -0.102, *β*_SLF_ = -0.035). We hypothesize that this observed association could be explained by structural differences in resource accessibility, health service infrastructure, or social support systems across regions with different economic development levels. Specifically, it is plausible that economically disadvantaged regions may face inadequate healthcare infrastructure and potentially delayed treatment-seeking behaviors, which might compound existing disease burdens. However, because our study did not directly measure individual-level healthcare access, treatment timing, or health system characteristics, these mechanisms remain speculative and require confirmation in future studies specifically designed to test causal pathways. These findings align with prior studies: Fürst et al. (2012) demonstrated that poverty and soil-transmitted helminth co-infections synergistically reduced HRQL in Côte d’Ivoire [24], while Jia et al. identified poverty as a key driver of chronic disability in schistosomiasis [15]. In China, integrating health education into national schistosomiasis control programs could enhance economic productivity and HRQL by facilitating early diagnosis and treatment [25]. However, our findings underscore the need to strengthen economic interventions in low-GDP regions to address persistent inequalities. Targeted subsidies for older patients, who experience the most pronounced quality-of-life reductions, may mitigate age-related vulnerabilities [26].

While physical health impairments have been predominantly documented in schistosomiasis research, our study underscores severe mental health deficits. Reductions in vitality (VT) and social functioning (SF) contributed to significantly lower mental component summary (MCS) scores in AS patients (71.95 *vs.* 80.80 in HP). These findings align with earlier reports by Kamel et al. (2002), who linked schistosomiasis to anxiety, depression, and reduced workplace productivity in Egypt [27]. Similarly, Xiao (2013) and Zhou et al. (2014) observed a high prevalence of depression among late-stage patients in China, attributing it to social isolation and economic strain [28,29]. Gender disparities further associated mental health outcomes, with women exhibiting lower MCS scores—a trend potentially tied to gendered caregiving responsibilities and healthcare access barriers, as noted by Kinung’hi et al. (2016) in sub-Saharan Africa [30].

An interesting contextual finding was the positive association between residing in areas that had reached schistosomiasis elimination status (ESS) and higher PCS/MCS scores among AS patients. While this correlation might reflect the benefits of sustained control programs and associated healthcare infrastructure improvements in these areas, it must be interpreted with caution. This association could also be influenced by unmeasured area-level confounders (e.g., overall socioeconomic development, healthcare access) or model-specific factors. Therefore, it should not be construed as direct evidence of a causal program effect but rather as a hypothesis-generating observation warranting further targeted investigation. China’s National Schistosomiasis Control Programme prioritizes surveillance and community education [25], strategies that likely contributed to the observed HRQL improvements. However, persistently low HRQL gains in AS patients [31] indicate insufficient post-treatment support systems. Integrating mental health services into existing control programs represents a critical opportunity to bridge this gap. Like positive psychology interventions [32] and structured nurse-led counseling [28] demonstrate efficacy in alleviating anxiety and enhancing HRQL among chronic disease cohorts.

An unexpected yet noteworthy finding emerged from our multivariable regression: the direction of association between ESS and HRQL differed across disease stages. Among AS patients, residing in areas that had officially achieved schistosomiasis elimination was positively associated with both PCS (*β* = 0.122, *P* < 0.001) and MCS (*β* = 0.110, *P* < 0.001). In contrast, among SLF patients, elimination status was negatively associated with MCS scores (*β* = -0.057, *P* = 0.038), with no significant association with PCS. To our knowledge, this opposite-direction finding has not been previously reported and warrants careful interpretation. One plausible explanation involves surveillance intensity and detection bias. In elimination-certified areas, the healthcare system maintains highly sensitive surveillance for schistosomiasis, which may lead to the identification and registration of SLF patients with milder or subclinical symptoms who might otherwise remain undiagnosed in non-elimination areas. The inclusion of these “healthier” SLF patients could paradoxically lower the group’s average HRQL if they perceive their newly labeled condition as a source of anxiety or stigma—an effect less likely to occur in AS patients whose severe symptoms are unequivocal and well-recognized. Alternatively, the positive association in AS patients may reflect tangible benefits from targeted social support programs that are often strengthened during the elimination phase (e.g., regular home visits, subsidized medications, rehabilitation services), which disproportionately benefit the most disabled group. A third possibility is residual confounding by unmeasured area-level factors, such as the density of primary care providers, local economic subsidies for chronic patients, or differential health-seeking behaviors. Given the cross-sectional design and the exploratory nature of this subgroup analysis, the opposite associations should be interpreted as hypothesis-generating rather than causal. Future mixed-methods studies incorporating patient interviews and health system audits are needed to elucidate the mechanisms underlying this stage-specific heterogeneity.

To this end, the SF-36 scale demonstrated strong discriminatory power in distinguishing HRQL across disease stages, validating its utility in schistosomiasis research. For instance, PF, BP, and GH scores were significantly lower in AS patients than in HP patients, aligning with previous validations in Chinese populations [19,33]. It should be noted that the PCS and MCS scores used in this study were derived from the norm-based weighted scoring algorithm recommended for the Chinese version of the SF-36, which enhances comparability with other studies using the same standardized approach [34]. However, cultural nuances, such as urban-rural disparities in China, necessitate local adaptations. For example, studies in Chinese schistosomiasis patients have shown that the SF-36’s domain structure is sensitive to liver fibrosis severity, with general health (GH) and vitality (VT) scores correlating with clinical markers of hepatic dysfunction [19,35], underscoring the importance of disease-specific contextual interpretation.

In summary, by stratifying and analyzing the AS and SLF groups, this study provided novel insights into stage-specific HRQL profiles. Patients with AS exhibited irreversible liver damage and multidimensional deficits, aligning with Ning et al.’s findings of severe hepatic dysfunction in comparable cohorts [14]. Notably, weaker associations between SLF and mental health outcomes challenge assumptions of uniform psychosocial impacts across disease stages. Socioeconomic factors, particularly low P-GDP, emerged as critical correlates of HRQL. Regions with low P-GDP frequently lacked accessible healthcare resources, which may be associated with delayed interventions for AS patients, underscoring structural inequalities that could exacerbate disease burden. These results align with the World Health Organization (WHO) 2030 Roadmap, which emphasizes integrated interventions targeting both biomedical and socioeconomic correlates [20].

### Limitations

Several methodological limitations should be considered when interpreting the findings of this study. These limitations pertain to study design, sample representativeness, measurement of key variables, and statistical considerations.

*Study design constraints*: The cross-sectional design precludes any causal or temporal inferences regarding the observed associations between disease stage, socioeconomic factors, and HRQL. Additionally, the SF-36 instrument has inherent methodological considerations: the norm-based algorithm used to compute PCS and MCS scores, while validated in Chinese populations, may not fully capture culturally specific dimensions of well-being; ceiling effects in the Role-Physical and Role-Emotional dimensions (as evident in our data) may reduce sensitivity in discriminating higher-functioning subgroups.

*Representativeness and generalizability*: Our findings are subject to several sampling constraints. First, the study was conducted exclusively in Jiangxi Province, which limits generalizability to regions with different epidemiological profiles (e.g., hilly vs. marshland areas) or healthcare infrastructures. Second, the matched case-sectional design—while enhancing comparability and statistical power for inter-group contrasts—resulted in a sample that does not reflect the true distribution of AS, SLF, and HP in the source community. Thus, our results should be interpreted as revealing associational patterns rather than estimating population prevalence or absolute HRQL burden. Third, we excluded participants with major chronic comorbidities (cardiovascular disease, diabetes, renal disorders, hepatitis, or cirrhosis) to strengthen internal validity; this necessarily limits generalizability to real-world populations where multimorbidity is common and may introduce selection bias.

*Measurement and modeling limitations*: We did not collect individual-level data on potential confounders such as education, occupation, or healthcare access. The socioeconomic indicators (P-DIRH, P-GDP) are county-level contextual variables, and their dichotomization (high/low relative to provincial average) risks ecological fallacy when interpreting associations at the individual level and may result in loss of statistical information. Furthermore, our regression models did not account for potential clustering of responses by geographic unit (e.g., county or township), which could affect standard error estimates.

*Statistical considerations*: Although we used the Enter method to avoid variable selection bias inherent in stepwise regression, the multiple comparisons conducted across different groups and outcomes increase the risk of Type I error. Readers should interpret the reported p-values with this in mind.

*Future directions*: To address these limitations, future research should prioritize longitudinal studies to track HRQL changes following treatment and evaluate intervention effectiveness. Comparative analyses across diverse epidemiological settings (e.g., marshland/lake versus hilly/mountainous regions) could elucidate environmental influences on HRQL. Mixed-methods studies integrating qualitative evidence—such as patient narratives or caregiver perspectives—may uncover unmet psychosocial needs and guide patient-centered rehabilitation models. Finally, expanding socioeconomic indicators (e.g., household debt, healthcare affordability) would enhance understanding of structural barriers to health equity.

## Methods

### Ethical considerations

Ethical approval for this study was obtained from the Medical Ethics Committee of the Jiangxi Provincial Institute of Parasitic Diseases (No. 2023–008). All study procedures adhered to the committee’s ethical guidelines and regulations.

All participants provided written informed consent after being fully informed of the study’s purpose, procedures, and nature. During data collection, participants retained the right to withdraw their data at any time. All personal identifiers were removed to ensure anonymity.

### Participants

This study employed a cross-sectional design and was conducted in schistosomiasis-endemic counties of Jiangxi Province, China. The study population comprised three groups:

(i) *Advanced schistosomiasis (AS)*: Participants were diagnosed according to the national standardized diagnostic criteria for schistosomiasis (WS261–2006) and exhibited complications such as portal hypertension syndrome with liver fibrosis, severe growth/developmental disorders, or significant granulomatous proliferation in the colon due to chronic pathological progression following inadequate treatment.
(ii) *Schistosomiasis-induced liver fibrosis (SLF)*: Individuals were confirmed to have liver fibrosis caused by *Schistosoma* infection, characterized by egg deposition in the liver, granulomatous inflammatory reactions, portal/periportal inflammation, and vascular fibrosis, but without progression to AS [14].
(iii) *Healthy population (HP)*: Residents of schistosomiasis-endemic areas with no history of *Schistosoma* infection.

This study adopted a matched cross-sectional design. The sampling procedure was as follows: First, from the historical schistosomiasis endemic counties in Jiangxi Province, we selected all counties that had at least one registered AS patient on the official provincial patient list (Jiangxi Provincial Institute of Parasitic Diseases registry, updated to December 2023). Second, within each selected county, all patients who met the national diagnostic criteria and were registered on the AS patient list were invited to participate if they met the inclusion and exclusion criteria. The AS group served as the reference (index) group, while the SLF and HP groups were matched at a 1:1:1 ratio. Matching was performed using a hierarchical rule: (i) priority was given to matching within the same township; (ii) if no eligible control participant was identified in the same township after complete screening of the candidate pool, matching was then performed within the same county but outside the index township. Using this protocol, township-level matching was achieved for 91.3% (807/884) of AS participants. For the remaining 8.7% (77/884), county-level matching was applied. All other matching criteria (gender, age ± 5 years) remained unchanged.

Inclusion criteria were: (i) permanent residence (not less than 10 years) within the boundaries of the surveyed district; (ii) age between 20–75 years; (iii) normal thinking ability and ability to answer questions clearly and accurately; and (iv) consent to participate in the study after being informed of the background of the survey and the content of the situation. Exclusion criteria comprised individuals with chronic comorbidities known to significantly impair quality of life, including cardiovascular diseases, tumors, diabetes mellitus, renal disorders, hepatitis, or cirrhosis. If there were multiple eligible candidates in the same matching pool, one person was randomly selected using the random number table method.

The study aims to compare differences in HRQL among populations at different disease stages (AS, SLF, HP). To this end, we adopted a case-sectional design, setting the AS group as the reference group and constructing the SLF and HP groups through 1:1:1 matching. This matched design is intended to maximize comparability among the three groups to clearly reveal the association between disease stage and HRQL; it does not imply a longitudinal follow-up (cohort) structure. The results should not be used to infer the actual prevalence of AS, SLF, or HP in the source population, nor the absolute HRQL levels in the general population.

### Sample size estimation

The primary goal of this study is to detect differences in the SF-36 PCS scores between pairs of groups, specifically AS *vs.* HP and AS *vs.* SLF. Although the study involves three groups, the core comparative design a matched cross-sectional comparison, with AS as the reference group and SLF and HP as two independent control groups. Therefore, the sample size was calculated based on the comparison that is likely to yield the smallest expected difference, i.e., between AS and SLF, as this represents the most conservative basis for power calculation.

We estimated σ (standard deviation) = 20 and δ (minimum clinically important difference) = 10, set *Z*_α/2_ (two-tailed test) = 1.96, and *Z*_β_ (power of 90%) = 1.28. Using the standard formula for two independent samples: $\text{n}=\frac{{\left({\text{Z}}_{\alpha /\text{2}}+{\text{Z}}_{\beta }\right)}^{\text{2}}\times \text{2}\times \sigma^{\text{2}}}{\delta^{\text{2}}}$, it was calculated that approximately 336 participants are needed per control comparison. To maintain balanced design and facilitate consistent pairwise comparisons across both contrasts, we set the sample size to 336 per group, resulting in a total of 336 × 3 = 1008 participants. This sample size also provides adequate power for the Kruskal–Wallis H test (a nonparametric alternative to one-way ANOVA) to detect overall differences among the three groups, given its asymptotic relative efficiency comparable to ANOVA.

To further validate the robustness of the sample size, we recalculated using a more conservative δ = 7.5 (reflecting a potentially smaller AS–SLF difference), which yielded a required sample size of approximately 590 per group. Our actual sample (n = 884 per group) still provides adequate power, confirming the study is well-designed for detecting differences between all three groups.

### Survey methodology

The survey spanned three months, from late May to early September 2024. HRQL assessments were conducted using the SF-36 scale and administered by trained investigators. Responses were recorded electronically via the “Questionnaire Star” platform (https://www.wjx.cn/), with mandatory fields implemented to ensure complete data entry. All data collection procedures were performed by professionals from local schistosomiasis control organizations, who underwent standardized training prior to the study.

### Assessment Instrument Scaling

The Chinese version of the SF-36 questionnaire was employed to evaluate participants’ HRQL. Studies comparing the SF-36 and EQ-5D-5L in AS patients found the SF-36 to be more sensitive to subtle declines in physical and social functioning [33,35]. This validated instrument comprises eight dimensions: physical functioning (PF), role limitations due to physical health (role-physical, RP), bodily pain (BP), general health (GH), vitality (VT), social function (SF), role limitations due to emotional problems (role-emotional, RE), and mental health (MH). These dimensions were aggregated into two composite scores: the physical component summary (PCS) (PF, RP, BP, GH) and the mental component summary (MCS) (VT, SF, RE, MH), representing physical and mental health outcomes, respectively. The Chinese version of the SF-36, has demonstrated high reliability and validity in both general and schistosomiasis patient populations [19,34].

The scoring of SF-36 follows a standard procedure: first, reverse-score specific items according to the questionnaire manual to ensure that high scores for all items represent better health status. Then, calculate the initial raw score for each dimension (the sum of scores for each item). Next, use a linear transformation formula to convert the raw score of each dimension into a standard score ranging from 0 to 100, with the formula as follows:

$Standardized\;Score=\frac{Raw\;Score-Lowest\;Possible\;Score}{Highest\text{ Possible Score}-\text{Lowest Possible Score}}\times \text{1}\text{0}\text{0}$

Finally, the PCS and MCS scores were then calculated using norm-based scoring algorithms. Specifically, the eight dimension standardized scores were aggregated using weight coefficients derived from factor analysis based on the Chinese general population norm (validated by Li et al. [34]). These coefficients account for the differential contributions of each dimension to physical and mental health constructs. This norm-based weighting method enables cross-study comparisons and avoids the bias inherent in simple arithmetic averaging.

This conversion ensured that all dimensions were normalized to a 0–100 scale, facilitating cross-dimensional comparisons [36]. Higher scores indicated better functional status and quality of life in each domain. As the final PCS and MCS scores are T-scores (mean = 50, SD = 10), they are interpreted relative to the Chinese general population norm: scores above 50 indicate better-than-average health status, while scores below 50 indicate worse-than-average health status.

### Classification by socio-economic and demographic characteristics

We collected socio-economic and demographic data from participants concurrently with the HRQL survey.

*Socio-economics v*ariables: The per-capita disposable income (P-DIRH) and per-capita gross domestic product (P-GDP) data used were sourced from the “Jiangxi Statistical Yearbook (2022)” at the county level. These are county-level contextual variables, reflecting the broader economic environment of participants’ residential areas rather than individual household economic status. For each indicator, we compared the county-level value against the provincial average: counties with value above the average were categorized as “High”, and those below as “Low”.

*Age (of a person)*: participants were stratified into two age groups: 20–59 years and 60–75 years [37].

*Type of schistosomiasis endemic area(TSE)*: In China, schistosomiasis-endemic areas are classified into three geographical types: marshland/lake regions, hilly/mountainous regions, and plain regions with waterway networks [38]. Jiangxi Province, however, comprises only marshland/lake and hilly/mountainous regions.

*Endemic status of schistosomiasis(ESS)*: According to the National Standardized for the Control and Elimination of Schistosomiasis (GB 15976–2015), the endemic status of each county was classified into two categories based on official assessments:

- Transmission interruption: All of the following criteria were met for five consecutive years prior to classification: (i) no locally acquired schistosomiasis cases; (ii) no locally infected animal reservoirs; (iii) no infected Oncomelania snails; and (iv) a functional surveillance system in place.
- Elimination: Achieved when an additional five consecutive years of zero local cases, zero infected animals, and zero infected snails were documented after transmission interruption criteria had already been satisfied.

Operationalization in this study: For each county included in our analysis, we applied the latest official endemic status as certified by the Health Commission of Jiangxi Provincial as of December 31, 2023 (i.e., the year prior to data collection). Counties were classified as “Elimination” if they had formally received elimination certification; otherwise, they were classified as “Transmission interruption” (no county in our dataset remained at the “transmission control” or “prevention” stage). This binary variable (elimination *vs.* interruption) was used in all regression models.

### Statistical analysis

Statistical analyses were conducted using SPSS Statistics 27.0 (SPSS Inc., Chicago, IL, USA). Two-tailed hypothesis tests were employed, with statistical significance set at *α* = 0.05.

Since the dimension scores exhibit a non-normal distribution, measures of central tendency are reported as the medians(M), first quartile (Q1), and third quartile (Q3) [*M(Q1, Q3)*]. Group comparisons were performed using the Kruskal-Wallis H test. When the overall test indicated significant differences (*P* < 0.05), post-hoc pairwise comparisons were conducted using the Bonferroni correction method to control for Type I error inflation due to multiple comparisons. The “*adj. P*” in the text refers to the *P* value after Bonferroni correction.

Multivariate linear regression analysis was employed to examine the associated between various factors and PCS, MCS scores. All prespecified independent variables (gender, age, P-DIRH, P-GDP, TSE, and ESS) were simultaneously entered into the model using the Enter method to avoid variable selection bias and significance inflation that may result from stepwise regression. Statistical significance was set at *P* ≤ 0.05. The standardized beta coefficients (*β*) are interpreted as the number of standard deviations by which the dependent variable (PCS/MCS) is expected to change for a one-standard-deviation increase in the independent variable. This allows comparison of the relative strength of associations across variables; however, for binary variables, their standard deviation is influenced by the category proportion, so *β* values should be interpreted cautiously in conjunction with the specific variable distribution.

Assessment of linear regression assumptions: Before interpreting the regression results, we systematically evaluated the underlying assumptions of linear regression. Normality of residuals was assessed using the Shapiro–Wilk test and visual inspection of Q-Q plots. Although the Shapiro–Wilk test indicated slight deviations from perfect normality (*P* < 0.05) due to the large sample size, the Q-Q plots showed residuals to be approximately normally distributed without severe skewness or heavy tails. Given the sample size (n = 884 per group), the central limit theorem supports the robustness of the regression estimates. Homoscedasticity was examined by plotting standardized residuals against predicted values; no clear funnel patterns or systematic increases in residual variance were observed, indicating that the assumption of constant error variance was reasonably met. Multicollinearity was assessed using variance inflation factor (VIF) and tolerance. All VIF values were below 2.0 (range: 1.03–1.89) and tolerance values exceeded 0.5, indicating no substantial multicollinearity among the independent variables. Independence of residuals was confirmed by the study design (independent observations with no clustering or repeated measures), and the Durbin–Watson statistic for the models ranged from 1.85 to 2.10, suggesting no significant autocorrelation. These diagnostic checks collectively support the appropriateness of linear regression for our analyses.

## Conclusion

This study conducted a stratified analyze HRQL differences between AS and SLF patients in a low-prevalence schistosomiasis setting in Jiangxi Province, China. By integrating regional economic and epidemiological data, this work explores the association between HRQL and socioeconomic contextual factors. The significant HRQL impairment observed in this study suggests that in areas with low prevalence, the extent of long-term health-related quality of life damage in patients may not be fully recognized by current strategies primarily focused on transmission control. Combining disease control measures with HRQL-focused interventions—such as integrating mental health support and economic assistance—may help mitigate the enduring damage caused by this neglected tropical disease. To align with the World Health Organization’s schistosomiasis elimination goals [20], multidimensional strategies are imperative. Future efforts must prioritize equitable healthcare access, holistic rehabilitation, and multisectoral collaboration to achieve comprehensive health recovery in schistosomiasis-affected communities. These findings highlight associations between disease stage, socioeconomic context, and HRQL, which warrant further investigation in longitudinal or interventional studies to establish causal pathways.

## Supporting information

S1 Data List of raw questionnaire data. (XLSX)
