# Supplementary material for: Assessing Health-Related Quality of life and socioeconomic impacts in low-endemic schistosomiasis: A study from Rural China
Source: PLoS Negl Trop Dis. 2026 Jul 16;20(7):e0014544. doi: 10.1371/journal.pntd.0014544 (PMC13374899; doi:10.1371/journal.pntd.0014544)
Supplement: Attachment — Submitted filename: Response to Reviewers.pdf [file pntd.0014544.s003.pdf]

Dear Reviewers:

Thank you for your interest in our manuscript entitled “Assessing Health-Related Quality of Life and Socioeconomic Impacts in Low-Endemic Schistosomiasis: A Study from Rural China”. We have studies comments carefully and have made correction which we hope meet the final approval. The alterations in the text are highlighted in the paper as requested. The main corrections in the paper and the responses to the reviewer’s comments are as following:

Methods

Reviewer #2:

The study objective is clearly stated, but it is framed broadly and not articulated as a prespecified, testable hypothesis. Moreover, terms such as “influencing factors,” “determinants,” or references to “risk/protective factors” imply causality that is not supported by the cross-sectional design. The design is appropriate for describing and comparing HRQL across disease stages (AS, SLF, HP) and for assessing associations with covariates, but it cannot substantiate causal interpretations or mechanistic claims.

Response: Thank you for your valuable comments on this paper. Your two points— that the research objectives are somewhat broad and lack predefined, testable hypotheses, and that certain terms in the text (such as 'influencing factors' and 'risk/protective factors') may imply causal relationships, which is inconsistent with a cross-sectional design—are very insightful and constructive. We have carefully read and understood your concerns, and made corresponding revisions and clarifications to the original manuscript. Below are our specific responses and explanations of the modifications:

1. You pointed out that the research objectives are somewhat broad and do not clearly state pre-defined testable hypotheses. We fully agree with this point. The aim of this study is to **describe** and **compare** health-related quality of life (HRQL) across different disease stages of schistosomiasis in low-endemic areas (AS, SLF, HP), and to **explore** its associations with socioeconomic and geographic factors, rather than testing a causal hypothesis.

To address this, we have refined the statement of research objectives in the **abstract** and **introduction** sections, explicitly clarifying the **descriptive** and **exploratory** nature of the study:

(1) **In the abstract**, we clearly define the goal as: “*To describe and compare health-related quality of life (HRQL) across disease stages (advanced schistosomiasis [AS], schistosomiasis-induced liver fibrosis [SLF], and healthy*

controls [HP]) in a low-endemic setting, and to explore associations between HRQL and socioeconomic/geographic factors."

(2) **At the end of the introduction**, we add a note: "*This study employed a descriptive and exploratory approach to compare HRQL across disease stages and to identify factors associated with HRQL outcomes, without implying causal mechanisms.*"

2. You correctly pointed out that using terms such as 'determinants', 'risk/protective factors' in cross-sectional studies may inappropriately imply causal relationships. We have comprehensively revised the entire text to avoid these potentially misleading terms and have uniformly adopted more neutral descriptive language instead.

- Replace "determinants" or "influencing factors" with "factors associated with" or "correlates".
- Replace "risk factor" with "factor associated with lower HRQL".
- Replace "protective factor" with "factor associated with higher HRQL".
- In statistical analysis and result description, clearly emphasize "association" rather than "influence" or "effect".

For example:

Original sentence: "Age ≥60 years was identified as the primary risk factor for HRQL decline."

Modify to: "*Age ≥60 years was most strongly associated with lower HRQL scores*."

3. We have further strengthened the description of research design limitations in the discussion and conclusion sections, emphasizing that the findings of this study are only cross-sectional associations and should not be interpreted as causal relationships.

At the beginning of the discussion, we add: "*Given the cross-sectional design, the observed associations should be interpreted as correlational and do not imply causation.*"

In the conclusion, we clearly state: "*These findings highlight associations between disease stage, socioeconomic context, and HRQL, which warrant further investigation in longitudinal or interventional studies to establish causal pathways.*"

The study population is described, yet key aspects remain insufficiently clear for appraisal of

internal and external validity—particularly the sampling strategy, the administrative/unit level of contextual variables (P-DIRH, P-GDP, ESS, TSE), and the implications of the equal group allocation. This design supports between-group comparisons, but it limits inference about population-level HRQL distributions or average HRQL in the underlying community.

The sample size is large and likely adequate to detect between-group differences; however, no formal sample size or power calculation is provided, and power for analyses involving area-level predictors may depend on the number of geographic units represented.

Response: Thank you very much for your careful review of this paper and for providing highly professional and important comments on the research methods section. The points you raised regarding sampling strategy, definition of background variables, rationality of research design, and sample size calculation are crucial for enhancing the transparency, rigor of this study, and readers' assessment of its validity. We have made detailed supplements and clarifications in the original manuscript's "Methods" section based on each of your comments. Below are our specific responses and modification explanations:

1. You correctly pointed out that the original manuscript did not clearly describe how the study population was selected. We have added a detailed sampling process in the 'Methods - Participants' section to enhance the assessability of the study's internal and external validity.

The following content is added:

"*This study adopted a two-stage cluster sampling method. First, all Schistosomiasis japonica endemic counties in Jiangxi Province that cover AS patients were selected from the historical endemic counties of schistosomiasis in Jiangxi Province. Second, within each selected county, all patients who met the national diagnostic criteria and were registered on the AS patient list were invited to participate if they met the inclusion and exclusion criteria.*"

"*If there were multiple eligible candidates in the same matching pool, one person was randomly selected using the random number table method.*"

2. You mentioned that the administrative levels of variables P-DIRH, P-GDP, ESS, and TSE are unclear, which indeed affects readers' understanding of the ecological association level of the data. We have clarified the data sources and units of all regional-level variables in the 'Methods - Classification of Socioeconomic and Demographic Characteristics' section.

The modified content is as follows:

"*The per-capita disposable income (P-DIRH) and per-capita gross domestic product (P-GDP) data used were sourced from the "Jiangxi Statistical Yearbook (2022)" at the county level. We compared the P-DIRH and P-GDP values of the surveyed counties with the provincial average. Those higher than the average were categorized as "High", and those lower than the average were categorized as "Low".*"

3. You pointed out that while group design is beneficial for inter-group comparisons, it limits inference about the overall distribution or average level of HRQL at the community level. We fully agree with this view and have explained this in the opening section of "Methods - Participants" and the "Discussion - Limitations" section.

Add at the end of the 'Methods - Participants' section:

"*The study aims to compare differences in health-related quality of life (HRQL) among populations at different disease stages (AS, SLF, HP). To this end, we adopted a case-control study approach, setting the AS group as the 'case' baseline and constructing the SLF group and HP group through equal matching. This design is intended to maximize comparability between groups to clearly reveal the association between disease severity and HRQL; however, its results should not be used to infer the actual prevalence of AS, SLF, or HP in the source population or the overall average HRQL level.*"

In the 'Discussion - Limitations' section, add:

"*More importantly, the matched case-control design, while enhancing comparability and statistical power for inter-group contrasts, resulted in a sample that does not reflect the true distribution of disease stages in the underlying community. Therefore, the findings are applicable to revealing association patterns but should not be extrapolated to estimate the overall HRQL burden in the endemic population.*"

4. You mentioned that formal sample size calculations were not provided, and the analytical power for regional-level variables depends on the number of geographic units. This is a very valid point. We have calculated the sample size and added a 'Sample Size Estimation' subsection in the 'Methods' section. The following is the new content to be added (**placed either after the 'Participants' section**):

"*Sample size estimation*

The primary goal of this study is to detect differences in the SF-36 scores

among the three groups: AS, SLF, and HP. We estimated σ (standard deviation) = 20 and δ (minimum clinically important difference) = 10, set Z α /2 (two-tailed test) = 1.96, and Z β (power of 90%) = 1.28. Using the formula $n = \frac{(Z_{\alpha/2}+Z_\beta)^2 \times 2 \times \sigma^2}{\delta^2}$*, it was calculated that approximately 336 participants are needed per group, resulting in a total sample size of no less than 1008 people.*"

Statistical analyses are partly appropriate: summarising SF-36 scores with medians and IQRs is reasonable given non-normality. Nonetheless, the use of the median test is nonstandard and low power compared with Kruskal–Wallis tests with appropriate post-hoc procedures. The manuscript reports "adjusted P" values without specifying the multiple-comparison correction method. The regression approach raises additional concerns: linear regression on bounded 0–100 outcomes can be acceptable, but model diagnostics and robustness checks are not reported; Standardized β coefficients should not be interpreted as 'unit changes', they represent changes in the outcome (in SD units) per 1 SD change in the predictor and are particularly hard to interpret for binary predictors, where the SD depends on category prevalence; backward/stepwise selection is prone to unstable variable selection and inflated significance unless justified and supported by sensitivity analyses. Further, potential clustering is not clearly addressed despite the inclusion of area-level predictors. Finally, SF-36 scoring is not described with sufficient detail for reproducibility (the manuscript suggests a generic 1,2,3… item coding approach without explicitly reporting the SF-36 item-specific recoding/reverse-scoring rules and missing-item handling), and the PCS/MCS computation appears to rely on a simple unweighted average of four domains rather than the standard norm-based, weighted PCS/MCS algorithm.

Response: Thank you very much for your professional review of the statistical methods section of this paper. Your comments are extremely fair and have helped us identify several deficiencies in the method description. We have carefully analyzed each of your suggestions and made corresponding clarifications and revisions in the "Methods" section of the manuscript. Below are our specific responses and modifications:

1. You correctly pointed out that for non-normally distributed data, the Kruskal-Wallis test is more commonly and efficiently used than the median test. We have accepted this suggestion and clarified the multiple comparison correction method.

Modify the content (the '**Statistical Analysis**' section):

"*Since the dimension scores showed non-normal distribution, measures of*

central tendency are presented as medians and interquartile ranges (IQRs) [M(IQR)]. Group comparisons were performed using the Kruskal-Wallis H test. When the overall test indicated significant differences (P < 0.05), post-hoc pairwise comparisons were conducted using the Bonferroni correction method to control for Type I error inflation due to multiple comparisons. The 'adj. P' in the text refers to the P value after Bonferroni correction."

2. The several concerns raised about the regression model are the focus of our revisions. We have adjusted the analytical methods and added necessary explanations. Furthermore, given that the SF-36 scores are widely distributed between 0 and 100 without excessive clustering at the boundaries, linear regression has been shown to be suitable for association analysis of such data. We examined the model's residual plots and found no obvious heteroscedasticity or non-linear patterns.

Modify the content (the '**Statistical Analysis**' section):

"*A multiple linear regression analysis was used to explore the associations between various factors and PCS, MCS scores. All preset predictor variables (gender, age group, P-DIRH, P-GDP, TSE, ESS) were simultaneously included in the model using the Enter method to avoid variable selection bias and significance inflation that may result from stepwise regression.*"

"*The standardized beta coefficients (β) in the reported were interpreted as: the number of standard deviations by which the dependent variable (PCS/MCS) is expected to change for a one-standard-deviation increase in the independent variable. This is used to compare the relative strength of associations of different variables with the outcome, but it should be noted that for binary variables, their standard deviation is influenced by the proportion of categories, so the magnitude of their β values needs to be cautiously interpreted in conjunction with the specific distribution of the variable.*"

3. We agree that the original manuscript's description of the scoring process was overly brief, which affected the reproducibility of the research. We have now supplemented it with detailed standardized scoring procedures and moved the revised content to the 'Assessment Instrument Scaling' subsection. In addition, since this survey was conducted online, each dimension had specified completion conditions (ensuring data integrity), so there is no missing data for any dimension.

Modify the content:

"*The scoring of SF-36 follows a standard procedure: first, reverse-score specific items according to the questionnaire manual to ensure that high scores for all items represent better health status. Then, calculate the initial raw score for each dimension (the sum of scores for each item). Next, use a linear transformation formula to convert the raw score of each dimension into a standard score ranging from 0 to 100, with the formula as follows:*

$$Standardized\ Score = \frac{Raw\ Score - Lowest\ Possible\ Score}{Highest\ Possible\ Score - Lowest\ Possible\ Score} \times 100$$

Finally, the Physical Component Summary (PCS) and Mental Component Summary (MCS) scores are not simple arithmetic averages of the four-dimensional standard scores, but are weighted summaries derived using weight coefficients based on the Chinese norm (validated by Li et al. [23]). This algorithm more accurately reflects the different contributions of physical and mental health to the total score."

Reviewer #3:

The study design and population groups are described in detail; however, a clearly articulated study objective and explicit, testable hypothesis are not stated in this section. While it is implied that the authors aim to compare health-related quality of life across populations with advanced schistosomiasis, schistosomiasis-induced liver fibrosis, and healthy individuals, this should be explicitly stated, ideally with a directional hypothesis. Clarifying whether the authors hypothesize graded HRQL impairment across disease severity would strengthen the conceptual framing and guide the interpretation of results.

Response: Thank you for your valuable comments on this paper. Your two points— that the research objectives are somewhat broad and lack predefined. We have carefully read and understood your concerns, and made corresponding revisions and clarifications to the original manuscript. Below are our specific responses and explanations of the modifications:

You pointed out that the research objectives are somewhat broad and do not clearly state pre-defined testable hypotheses. We fully agree with this point. The aim of this study is to **describe** and **compare** health-related quality of life (HRQL) across different disease stages of schistosomiasis in low-endemic areas (AS, SLF, HP), and to **explore** its associations with socioeconomic and geographic factors, rather than testing a causal hypothesis.

To address this, we have refined the statement of research objectives in the **abstract** and **introduction** sections, explicitly clarifying the **descriptive** and

exploratory nature of the study:

(3) **In the abstract**, we clearly define the goal as: "*To describe and compare health-related quality of life (HRQL) across disease stages (advanced schistosomiasis [AS], schistosomiasis-induced liver fibrosis [SLF], and healthy controls [HP]) in a low-endemic setting, and to* explore associations between HRQL and socioeconomic/geographic factors."

(4) **At the end of the introduction**, we add a note: "*This study employed a descriptive and exploratory approach to compare HRQL across disease stages and to identify factors associated with HRQL outcomes, without implying causal mechanisms.*"

The cross-sectional design is appropriate for comparing HRQL outcomes across disease states at a single time point. Given that the study's aim appears to be descriptive and comparative rather than causal, the chosen design is acceptable. However, the limitations inherent to cross-sectional studies, particularly the inability to infer temporality or causation between disease status and HRQL outcomes, should be acknowledged explicitly in the manuscript.

Response: Thank you very much for your correctly pointed out. We may have inappropriately implied a causal relationship by using terms such as 'determinants', 'risk/protective factors' in cross-sectional studies may inappropriately imply causal relationships. We have comprehensively revised the entire text to avoid these potentially misleading terms and have uniformly adopted more neutral descriptive language instead.

- Replace "determinants" or "influencing factors" with "factors associated with" or "correlates".
- Replace "risk factor" with "factor associated with lower HRQL".
- Replace "protective factor" with "factor associated with higher HRQL".
- In statistical analysis and result description, clearly emphasize "association" rather than "influence" or "effect".

For example:

Original sentence: "Age ≥60 years was identified as the primary risk factor for HRQL decline."

Modify to: "*Age ≥60 years was most strongly associated with lower HRQL scores.*"

At the same time, we have further strengthened the description of research design limitations in the discussion and conclusion sections, emphasizing that

the findings of this study are only cross-sectional associations and should not be interpreted as causal relationships.

At the beginning of the discussion, we add: "*Given the cross-sectional design, the observed associations should be interpreted as correlational and do not imply causation.*"

In the conclusion, we clearly state: "*These findings highlight associations between disease stage, socioeconomic context, and HRQL, which warrant further investigation in longitudinal or interventional studies to establish causal pathways.*"

The study population is well defined, with clear diagnostic criteria for AS and SLF based on national standards. The matching strategy by county, gender, and age enhances comparability between groups and is a strength. The exclusion of individuals with chronic comorbidities that significantly affect quality of life is appropriate for reducing confounding. However, this may limit generalizability to real-world schistosomiasis populations where comorbidities are common. This trade-off should be discussed.

Response: We sincerely thank the reviewer for the positive feedback on the clarity of our study population definitions and the rigorous matching strategy, as well as for raising the important point regarding the exclusion of comorbidities. We fully agree that this design choice involves a trade-off between internal validity and generalizability.

In the revised manuscript, we have added a dedicated paragraph in the Limitations section to explicitly discuss this issue (see below). We acknowledge that by excluding individuals with significant chronic comorbidities, our sample may not fully represent the real-world schistosomiasis population, among whom comorbidities are indeed common. However, this exclusion was necessary to isolate the specific effect of schistosomiasis disease stage on HRQL and to minimize confounding by other health conditions that independently impair quality of life. This approach strengthens our ability to draw clearer conclusions about the associations between schistosomiasis severity and HRQL.

We believe this clarification adds transparency to our methodological choices and helps readers appropriately interpret the study's findings and their applicability. Thank you again for this valuable suggestion.

Supplementary content:

"*Furthermore, the exclusion of participants with major chronic comorbidities (e.g., cardiovascular diseases, diabetes) strengthens internal validity but limits generalizability to real-world populations where comorbidities are prevalent, potentially introducing selection bias.*"

The manuscript does not report the total sample size per group in this section, nor does it describe any a priori sample size or power calculation. Without this information, it isn't easy to assess whether the study was adequately powered to detect meaningful differences in HRQL scores or associations in multivariate analyses. The authors should clarify the sample size rationale and discuss potential implications of insufficient power, if applicable.

Response: You mentioned that formal sample size calculations were not provided, this is a very valid point. We have calculated the sample size and added a 'Sample Size Estimation' subsection in the 'Methods' section.

The following is the new content to be added (**placed either after the 'Participants' section**):

"*Sample size estimation*

The primary goal of this study is to detect differences in the SF-36 scores among the three groups: AS, SLF, and HP. We estimated σ *(standard deviation) = 20 and* δ *(minimum clinically important difference) = 10, set* $Z_{\alpha/2}$ *(two-tailed test) = 1.96, and* Z_{β} *(power of 90%) = 1.28. Using the formula* $n = \frac{(Z_{\alpha/2}+Z_{\beta})^2 \times 2 \times \sigma^2}{\delta^2}$*, it was calculated that approximately 336 participants are needed per group, resulting in a total sample size of no less than 1008 people.*"

The use of nonparametric methods for non-normally distributed SF-36 scores is appropriate, and the normalization of scores to a 0-100 scale follows standard practice. The description of the PCS and MCS computation is clear. However, there are some concerns regarding statistical consistency. While group comparisons use nonparametric tests and medians, multivariate linear regression assumes normally distributed residuals and linear relationships. The authors should justify the use of linear regression for PCS and MCS scores, report diagnostic checks, or consider alternative models if assumptions are violated. In addition, backward elimination can increase the risk of overfitting and model instability; justification for this approach, along with the reporting of model diagnostics, would strengthen the analysis.

Response: The several concerns raised about the regression model are the focus of our revisions. We have adjusted the analytical methods and added

necessary explanations. Furthermore, given that the SF-36 scores are widely distributed between 0 and 100 without excessive clustering at the boundaries, linear regression has been shown to be suitable for association analysis of such data. We examined the model's residual plots and found no obvious heteroscedasticity or non-linear patterns.

Modify the content (the '**Statistical Analysis**' section):

"*A multiple linear regression analysis was used to explore the associations between various factors and PCS, MCS scores. All preset predictor variables (gender, age group, P-DIRH, P-GDP, TSE, ESS) were simultaneously included in the model using the Enter method to avoid variable selection bias and significance inflation that may result from stepwise regression.*"

Results

Reviewer #2:

The Results section is generally well organised and reports clear between-group differences in HRQL; however, several aspects require correction. First, a standard baseline characteristics table comparing AS, SLF, and HP is needed to document group comparability, provide clear denominators, and avoid relying on narrative summaries alone. Interpretation should be aligned strictly with the statistical findings: the stated “progressive decline/gradient” is well supported for PCS, but is less consistently supported for MCS (SLF vs HP is not clearly reported as significant), so conclusions should be qualified accordingly. The use of the median test is nonstandard and low power; please justify it or report Kruskal–Wallis tests with an appropriate post-hoc procedure, and specify the multiplicity correction underlying “adjusted P” values. Domain-level reporting also needs improvement: χ^2 statistics are presented without mapping to specific domains, and RP/RE exhibit pronounced ceiling effects (median 100 across groups), for which median (IQR) alone is insufficient—consider reporting Q1/Q3 explicitly and/or proportions at ceiling/floor.

Response: Thank you very much for your question. Regarding the non-parametric test method, we have reselected the Kruskal-Wallis test and corrected the test value, with no change in the overall test result. A detailed reply has already been provided previously. Concerning the median notation, we accept your suggestion and have revised all instances in the manuscript to M(Q1, Q3).

Regression results should be framed as associations rather than causal effects: current language over-claims (“determinants,” “risk factor,” “improved HRQL,” “protective factors”). Several

predictors (P-GDP, P-DIRH, ESS, TSE) are county/area-level contextual measures; interpreting them as individual-level exposures (e.g., “poverty” at household level, or “elimination improves HRQL”) risks ecological fallacy and should be avoided. Relatedly, interpretability of socioeconomic effects is limited because P-DIRH and P-GDP are dichotomized (high/low); therefore statements implying “unit reductions” are incorrect, and effect estimates should be expressed as differences between categories. The positive association between ESS “elimination” and PCS/MCS in the AS group warrants cautious interpretation and exploration (e.g., contextual confounding, or model instability), rather than being presented as evidence of program effect.

Response:

We sincerely thank the reviewer for this critical and insightful feedback regarding the interpretation of our regression results. The points raised are essential for ensuring the statistical rigor and appropriate inference of our findings. We fully agree with all comments and have revised the manuscript accordingly to address each concern.

Associations vs. Causal Effects: We have carefully reviewed the language throughout the manuscript, particularly in the Abstract, Results, and Discussion sections, and replaced terms that could imply causality (e.g., "determinants," "improved," "protective factors," "exacerbated") with more accurate correlational language (e.g., "were associated with," "correlated with," "linked to"). We have also added explicit reminders of the cross-sectional, non-causal nature of the study where appropriate.

Avoiding Ecological Fallacy: We appreciate the crucial reminder regarding the distinction between area-level and individual-level variables. In the revised manuscript, we have clarified the level of measurement for P-GDP, P-DIRH, ESS, and TSE in the Methods section. Throughout the Results and Discussion, we now explicitly frame these as area-level or contextual factors (e.g., "living in a county with low P-GDP was associated with..." or "residing in areas that achieved elimination status was correlated with..."), avoiding any interpretation that attributes these characteristics to individuals.

Correct Interpretation of Dichotomized Variables: The reviewer is correct that statements implying a "per-unit" change for dichotomized variables are inappropriate. We have removed any such phrasing (e.g., "a 1-unit reduction in P-GDP"). The effects of P-DIRH and P-GDP are now consistently described as differences between the "High" and "Low" categories (e.g., "participants in low P-GDP counties had lower PCS scores compared to those in high P-GDP

counties").

Cautious Interpretation of the ESS Association in AS Group: We have toned down the interpretation of the positive association between elimination status (ESS) and HRQL in AS patients. The revised Discussion now presents this as an interesting contextual finding that warrants further investigation, highlighting potential explanations such as unmeasured area-level confounders (e.g., better overall healthcare infrastructure in elimination areas) or model characteristics, rather than direct evidence of program impact.

These revisions significantly strengthen the methodological clarity and inferential caution of our paper. We are grateful for the reviewer's expertise in identifying these important issues.

The specific modification content is as follows:

1. Methods section (Classification by socio-economic and demographic characteristics):

Add at the end of the relevant paragraph: "*Note: P-DIRH and P-GDP are county-level contextual variables, not individual household measures.*"

2. Results section (Analysis of factors affecting HRQL...):

Change the title of Table 1 to: "*Table 1. Factors associated with SF-36 scores in a population of schistosomiasis patients (multivariable linear regression results)*"

In the text describing regression results, change vague expressions such as "significantly association PCS scores" to "*were significantly associated with PCS scores*".

3. Discussion section:

Find and modify all verbs that imply causality, such as "exacerbated" and "improved" (when referring to statistical associations), to "were associated with" and "co-occurred with".

Change the "Areas with lower P-GDP or P-DIRH exhibited poorer HRQL outcomes." to a more accurate "Residing in areas (counties) classified as having low P-GDP or P-DIRH was associated with poorer HRQL outcomes."

Remove the sentence containing "For instance, a 1-unit reduction in P-GDP was associated with decreased PCS scores..." in the original text. Replace it with: "*For instance, AS and SLF patients residing in counties categorized as ‘Low P-GDP’ had significantly lower PCS scores compared to those in ‘High P-GDP’ counties (βAS = -0.123, βSLF = -0.049).*"

We have reinterpreted the content marked with "The study identified key factor associated with higher HRQL, including regions that achieved schistosomiasis elimination, which demonstrated co-occurred with physical component summary (PCS; β = 0.114) and mental component summary (MCS; β = 0.109) scores among advanced schistosomiasis (AS) patients, offering actionable evidence for policy enhancement." in the 7th paragraph of the original discussion section, added cautious language, and revised it to "*An interesting contextual finding was the positive association between residing in areas that had reached schistosomiasis elimination status (ESS) and higher PCS/MCS scores among AS patients. While this correlation might reflect the benefits of sustained control programs and associated healthcare infrastructure improvements in these areas, it must be interpreted with caution. This association could also be influenced by unmeasured area-level confounders (e.g., overall socioeconomic development, healthcare access) or model-specific factors. Therefore, it should not be construed as direct evidence of a causal program effect but rather as a hypothesis-generating observation warranting further targeted investigation.*"

Finally, several reporting issues require correction: "SLP" in the table should be "SLF," p-values should follow standard conventions (e.g., report $P < 0.001$ rather than $P < 0.0001$), and regression tables should include sample size (N) per model, model fit (R^2/adjusted R^2), and—ideally—95% confidence intervals for coefficients.

Response: Thank you very much for your suggestions. We fully accept them and have added all the information to the table.

Table 1. Factors associated with SF-36 scores in a population of schistosomiasis patients (multivariable linear regression results)

Overall rating category	Significant Predictors	AS			SLF		
		β		*p*	*β*		*p*
		Value	*95% CI*		Value	*95% CI*	
PCS	Gender(Female)	-0.097	-5.236 ~ -1.910	<0.001	-0.112	-5.107 ~ -2.186	<0.001
	Age(60-75)	-0.223	-11.722 ~ -7.600	<0.001	-0.263	-11.890 ~ -8.269	<0.001
	P-DIRH(Low)	-0.048	-4.088 ~ 0.348	0.098	-0.020	-2.638 ~ 1.256	0.486
	P-GDP(Low)	-0.102	-6.830 ~ -2.142	<0.001	-0.035	-3.407 ~ 0.714	0.200
	TSE(Hilly/mountainous regions)	-0.019	-2.876 ~ 1.498	0.537	0.067	2.261 ~ 4.101	0.026
	ESS(Elimination)	0.122	2.777 ~ 7.115	<0.001	-0.033	-3.074 ~ 0.735	0.229
	Sample size (N)	844			844		
	Model fit (R^2/adjusted R^2)	0.281/0.079			0.298/0.089		
MCS	Gender(Female)	-0.093	-5.027 ~ -1.721	<0.001	-0.122	-5.539 ~ -2.548	<0.001
	Age(60-75)	-0.147	-8.268 ~ -4.170	<0.001	-0.170	-8.423 ~ -4.715	<0.001
	P-DIRH(Low)	-0.067	-4.758 ~ -0.348	0.023	-0.084	-4.906 ~ -0.919	0.004
	P-GDP(Low)	-0.031	-3.671 ~ 0.989	0.259	0.011	-1.685 ~ 2.534	0.693
	TSE(Hilly/mountainous regions)	-0.118	-6.434 ~ -2.086	<0.001	-0.070	-4.263 ~ -0.331	0.022
	ESS(Elimination)	0.110	2.178 ~ 6.490	<0.001	-0.057	-4.108 ~ -0.118	0.038
	Sample size (N)	884			844		
	Model fit (R^2/adjusted R^2)	0.208/0.043			0.253/0.064		

β: Standard Beta Coefficient; *95%CI*: 95% confidence interval for the unstandardized coefficient; AS: advanced schistosomiasis; SLF: schistosomiasis-induced liver fibrosis; PCS: physical component summary; MCS: mental component summary; P-DIRH: Per-capita disposable income of rural households; P-GDP: Per-capita gross domestic product; TSE: type of schistosomiasis endemic area; ESS: Endemic status of schistosomiasis

Improve the radar plot readability (Fig. 2). With filled polygons, overlapping colors blend and obscure group differences. It would be clearer to (i) plot the largest polygon first and the smallest last , and/or (ii) reduce fill opacity substantially and emphasize distinct outlines/markers. Alternatively, consider showing lines only (no fill).

Response: OK.

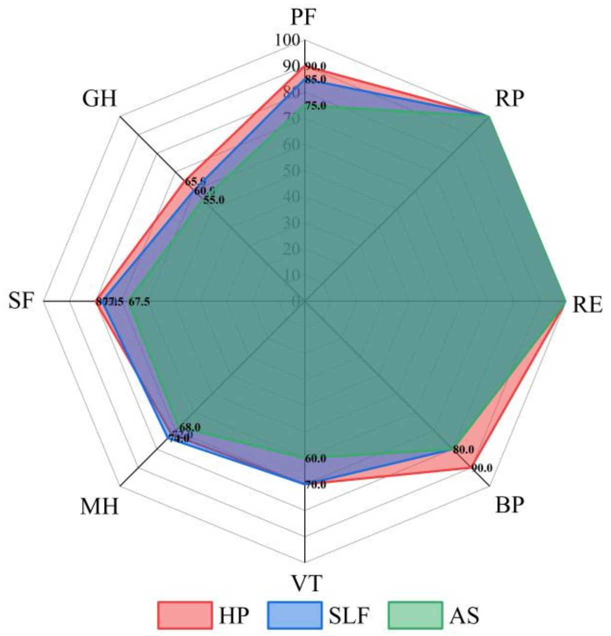

Reviewer #3:

Overall, the analyses presented align with the statistical methods described earlier. Group comparisons of PCS, MCS, and SF-36 dimension scores use nonparametric summaries and median-based tests, as planned. The use of multivariate linear regression to identify factors associated with PCS and MCS is consistent with the stated approach.

However, some elements of the analysis plan are not fully reflected in the results. The manuscript does not clearly describe how multiple pairwise comparisons were adjusted, despite reporting adjusted P values. The specific adjustment method should be stated and consistently applied. In addition, while backward regression is mentioned, there is no reporting of model diagnostics, goodness-of-fit measures, or checks of regression assumptions, which limits assessment of the robustness of the findings.

Response: Thank you very much for your suggestions. We fully accept them and have added all the information to the table.In addition, regarding "multiple comparisons", we have made supplements and modifications in the Methods section.

The modified content is as follows:

Multivariate linear regression analysis was used to employed the associated between various factors and PCS, MCS scores. All preset dependent variables (gender, age, P-DIRH, P-GDP, TSE, and ESS) were simultaneously included in the model useing Enter mothod to avoid variable selection bias and significance inflation that may result from stepwise regression. Statistical significance was set at $P \leq 0.05$. The

Table 1. Factors associated with SF-36 scores in a population of schistosomiasis patients (multivariable linear regression results)

Overall rating category	Significant Predictors	AS β Value	AS β 95% CI	AS p		SLF β Value	SLF β 95% CI	SLF p
PCS	Gender(Female)	-0.097	-5.236 ~ -1.910	<0.001		-0.112	-5.107 ~ -2.186	<0.001
	Age(60-75)	-0.223	-11.722 ~ -7.600	<0.001		-0.263	-11.890 ~ -8.269	<0.001
	P-DIRH(Low)	-0.048	-4.088 ~ 0.348	0.098		-0.020	-2.638 ~ 1.256	0.486
	P-GDP(Low)	-0.102	-6.830 ~ -2.142	<0.001		-0.035	-3.407 ~ 0.714	0.200
	TSE(Hilly/mountainous regions)	-0.019	-2.876 ~ 1.498	0.537		0.067	2.261 ~ 4.101	0.026
	ESS(Elimination)	0.122	2.777 ~ 7.115	<0.001		-0.033	-3.074 ~ 0.735	0.229
	Sample size (N)	844				844		
	Model fit (R^2/adjusted R^2)	0.281/0.079				0.298/0.089		
MCS	Gender(Female)	-0.093	-5.027 ~ -1.721	<0.001		-0.122	-5.539 ~ -2.548	<0.001
	Age(60-75)	-0.147	-8.268 ~ -4.170	<0.001		-0.170	-8.423 ~ -4.715	<0.001
	P-DIRH(Low)	-0.067	-4.758 ~ -0.348	0.023		-0.084	-4.906 ~ -0.919	0.004
	P-GDP(Low)	-0.031	-3.671 ~ 0.989	0.259		0.011	-1.685 ~ 2.534	0.693
	TSE(Hilly/mountainous regions)	-0.118	-6.434 ~ -2.086	<0.001		-0.070	-4.263 ~ -0.331	0.022
	ESS(Elimination)	0.110	2.178 ~ 6.490	<0.001		-0.057	-4.108 ~ -0.118	0.038
	Sample size (N)	884				844		
	Model fit (R^2/adjusted R^2)	0.208/0.043				0.253/0.064		

β: Standard Beta Coefficient; *95%CI*: 95% confidence interval for the unstandardized coefficient; AS: advanced schistosomiasis; SLF: schistosomiasis-induced liver fibrosis; PCS: physical component summary; MCS: mental component summary; P-DIRH: Per-capita disposable income of rural households; P-GDP: Per-capita gross domestic product; TSE: type of schistosomiasis endemic area; ESS: Endemic status of schistosomiasis

The Results section is generally well structured, progressing logically from sample characteristics to overall HRQL outcomes, dimension-level analyses, and multivariate modeling. Key findings are clearly stated, and the reporting of medians and IQRs is appropriate for non-normally distributed data.

Response: Thank you very much for your question. Concerning the median notation, we accept your suggestion and have revised all instances in the manuscript to M(Q1, Q3).

That said, some results would benefit from greater completeness and precision. The sample size per group is not explicitly reported in the Results, despite being critical for interpreting effect estimates and regression outputs. In the regression analyses, only standardized beta coefficients and P values are presented; confidence intervals and measures of explained variance (such as R^2) are not reported, which limits interpretability and comparison across models.

Response: Thank you very much for your suggestions. We fully accept them and have added all the information to the table.

Table 1. Factors associated with SF-36 scores in a population of schistosomiasis patients (multivariable linear regression results)

Overall rating category	Significant Predictors	AS β Value	AS β 95% CI	AS p	SLF β Value	SLF β 95% CI	SLF p
PCS	Gender(Female)	-0.097	-5.236 ~ -1.910	<0.001	-0.112	-5.107 ~ -2.186	<0.001
	Age(60-75)	-0.223	-11.722 ~ -7.600	<0.001	-0.263	-11.890 ~ -8.269	<0.001
	P-DIRH(Low)	-0.048	-4.088 ~ 0.348	0.098	-0.020	-2.638 ~ 1.256	0.486
	P-GDP(Low)	-0.102	-6.830 ~ -2.142	<0.001	-0.035	-3.407 ~ 0.714	0.200
	TSE(Hilly/mountainous regions)	-0.019	-2.876 ~ 1.498	0.537	0.067	2.261 ~ 4.101	0.026
	ESS(Elimination)	0.122	2.777 ~ 7.115	<0.001	-0.033	-3.074 ~ 0.735	0.229
	Sample size (N)	844			844		
	Model fit (R^2/adjusted R^2)	0.281/0.079			0.298/0.089		
MCS	Gender(Female)	-0.093	-5.027 ~ -1.721	<0.001	-0.122	-5.539 ~ -2.548	<0.001
	Age(60-75)	-0.147	-8.268 ~ -4.170	<0.001	-0.170	-8.423 ~ -4.715	<0.001
	P-DIRH(Low)	-0.067	-4.758 ~ -0.348	0.023	-0.084	-4.906 ~ -0.919	0.004
	P-GDP(Low)	-0.031	-3.671 ~ 0.989	0.259	0.011	-1.685 ~ 2.534	0.693
	TSE(Hilly/mountainous regions)	-0.118	-6.434 ~ -2.086	<0.001	-0.070	-4.263 ~ -0.331	0.022
	ESS(Elimination)	0.110	2.178 ~ 6.490	<0.001	-0.057	-4.108 ~ -0.118	0.038
	Sample size (N)	884			844		
	Model fit (R^2/adjusted R^2)	0.208/0.043			0.253/0.064		

β: Standard Beta Coefficient; 95%CI: 95% confidence interval for the unstandardized coefficient; AS: advanced schistosomiasis; SLF: schistosomiasis-induced liver fibrosis; PCS: physical component summary; MCS: mental component summary; P-DIRH: Per-capita disposable income of rural households; P-GDP: Per-capita gross domestic product; TSE: type of schistosomiasis endemic area; ESS: Endemic status of schistosomiasis

Additionally, the text occasionally strays toward interpretation (for example, statements implying improvement due to the elimination status), which would be more suitable for the Discussion section.

Response: Thank you very much for your questions and suggestions. We have supplemented and improved them in the discussion section.

In Figure 2, the reporting of multiple chi-square values in the text is unclear and may benefit from a clearer mapping between each statistic and the corresponding SF-36 dimension.

Response: OK, we have made the supplement, as follows:

> and GH: 65.0 (25.0). Tests revealed significant differences (*H* = 119.184[PF], 48.037[BP], 42.505[VT], 102.000[MH], 80.883[SF], 107.434[GH]; *all P* < 0.05) in all dimensions except RP and RE. Pairwise comparisons demonstrated that PF, VT, SF,

Conclusions

Reviewer #2:

However, the Discussion frequently overreaches the evidence base: causal language and framing ("determinants," "risk/protective factors," "improved HRQL," "progressive impact," "actionable evidence," "mechanisms") should be replaced with associational language consistent with a cross-sectional design, and the narrative should be aligned strictly with the reported results (the stage gradient is clearly supported for PCS, whereas MCS differences—particularly SLF vs HP—appear less consistent and should be qualified). Several contextual predictors (P-GDP, P-DIRH, ESS, TSE) are area-level measures; the Discussion should avoid individual-level

interpretations (household poverty; elimination causing HRQL improvements) and acknowledge potential ecological inference and residual confounding, especially given inconsistent ESS directions across groups.

Response: Thank you for your valuable comments on this paper. We fully agree with your correction regarding 'the discussion section often exceeding the evidence base,' particularly in the use of causal language, interpretation of cross-sectional study design, and explanation of region-level variables. These comments are crucial for enhancing the scientific rigor and accuracy of the paper's explanations. We have carefully read and fully understood your comments, and will make the following systematic and targeted revisions to the 'Discussion' section of the manuscript: ensuring that the language, framework, and interpretations are strictly consistent with the inherent nature of cross-sectional design, and clarifying the interpretation of region-level variables.

1. We will systematically review and revise the entire text (especially the 'Discussion' section), replacing all implicit or explicit causal expressions (such as 'determinants', 'risk/protective factors', 'improved HRQL', 'progressive impact', 'actionable evidence', 'mechanisms') with strictly associative language that describes observed co-occurrences or correlations.

Change "Our cross-sectional survey included 2,652 participants, and the results revealed a gradient in HRQL scores (AS < SLF < HP)" to "*Our cross-sectional survey included 2,652 participants, and the results revealed a gradually increasing trend in HRQL scores (AS < SLF < HP)*"

2. We have noted the issue you pointed out regarding the MCS differences (particularly between the SLF group and the HP group) being less consistent than the Physical Component Summary (PCS) of physical health. In the 'Discussion' section, we have added: "*Although an overall trend was observed (AS < SLF < HP), it was noted that the magnitude of MCS differences and statistical significance in the comparison between the SLF and HP groups may be weaker than that of PCS. This suggests that the manifestation of mental health impacts may not be entirely parallel to physical health impacts across different disease stages, which warrants further investigation in future studies.*"

3. **Clarify variable levels:** When discussing relevant results, P-GDP, P-DIRH, ESS, and TSE will always be explicitly emphasized as contextual variables at the county level. These variables reflect the overall economic level and

disease control status of the participants' regions, rather than their personal or household attributes.

Socio-economics variables: The per-capita disposable income (P-DIRH) and per-capita gross domestic product (P-GDP) data used were sourced from the "Jiangxi Statistical Yearbook (2022)" at the county level. We compared the P-DIRH and P-GDP values of the surveyed counties with the provincial average. Those higher than the average were categorized as "High", and those lower than the average were categorized as "Low". Note: P-DIRH and P-GDP are county-level contextual variables, not individual household measures.

Avoid individualized interpretation: Any statements that may directly imply individual poverty or family income will be deleted or modified. For example, discussions about poorer HRQL in areas with low per capita GDP will be rephrased the "Economic factors further exacerbated these disparities. Areas with lower P-GDP or P-DIRH were associated with poorer HRQL." as: "*Patients living in counties with lower overall economic development levels have lower HRQL scores compared to those living in counties with higher development levels, which may reflect structural differences in these regions regarding accessibility of medical resources, health service infrastructure, or social support systems.*"

The Conclusions are currently too strong for the design and analyses: claims of being the "first," of elucidating "mechanisms," and of demonstrating that current control strategies "underestimate" HRQL burdens or that specific interventions "can mitigate enduring damage" should be moderated and framed as implications/hypotheses rather than findings. A more defensible conclusion would emphasize stage-stratified HRQL differences and associated factors, and recommend longitudinal and multi-site studies to evaluate causal pathways and interventions.

Response: Thank you for your profound and important comments on the conclusion section of this paper. We fully agree that, within the framework of a cross-sectional study design, the expression in the conclusion section should

be more cautious, avoiding causal inferences and overly definitive statements that go beyond the data support. Your suggestions will help us make the conclusion more rigorous and better reflect the findings and limitations of this study. We have carefully read and fully understood your comments, and we will revise the 'Conclusion' section accordingly to weaken overly strong declarations, rephrase them as insights or hypotheses based on observed association patterns, and highlight recommendations for future research.

1. We will revise the opening statement to avoid absolute expressions such as "the first". Instead, we will emphasize that this study "*provides a stratified analysis of HRQL distinguishing between AS and SLF in a low-endemic setting*".

This study offers provides a stratified analysis of HRQL distinguishing between AS and SLF in a low-endemic setting and makes three key contributions: 1. Stage-specific insights: By distinguishing between AS and SLF cohorts, we identified distinct HRQL profiles, providing a foundation for stage-targeted rehabilitation interventions. 2. Socioeconomic integration: By integrating regional economic

Admin-O
删除: the first comprehensive analysis of health-related quality of life (HRQL) among schistosomiasis patients in low-transmission settings

Conclusion

This study conducted a stratify and analyze HRQL differences between AS and SLF patients in a low-prevalence schistosomiasis setting in Jiangxi Province, China. By

Admin-O
删除: is the first to

2. We will delete or modify any statements that imply the clarification of causal "mechanisms". For example, changing "addresses critical research gaps in ... socioeconomic impact mechanisms" to "*explores the association between HRQL and socioeconomic contextual factors' to indicate that we are exploring associations rather than mechanisms.*"

Conclusion

This study is the first to stratify and analyze HRQL differences between AS and SLF patients in a low-prevalence schistosomiasis setting in Jiangxi Province, China. By integrating regional economic and epidemiological data, this work explores the association between HRQL and socioeconomic contextual factors. Our findings suggest that HRQL impairments in low-prevalence schistosomiasis settings may be underestimated, as current strategies prioritize transmission control over long-term patient well-being. Combining disease control measures with HRQL-focused interventions—such as integrating mental health support and economic

Admin-O
删除:

Admin-O
删除: addresses critical research gaps in disease-stage-specific interventions and socioeconomic impact mechanisms

3. For the assertion that "HRQL burden may be underestimated" we will revise the wording, changing "Our findings suggest that HRQL impairments in low-prevalence schistosomiasis settings may be underestimated, as current strategies prioritize transmission control over long-term patient well-being." to "*The significant HRQL impairment observed in this study suggests that in areas with low prevalence, the extent of long-term health-related quality of life damage in patients may not be fully recognized by current strategies primarily*

focused on transmission control."

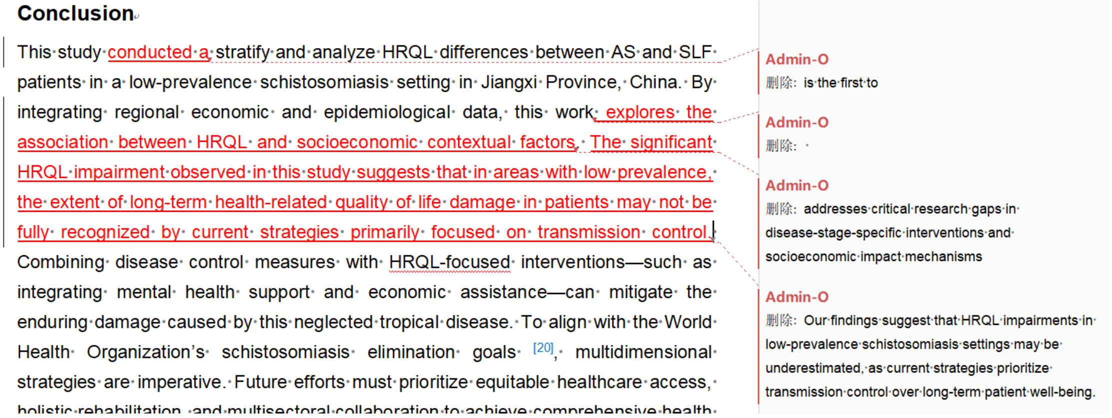

Conclusion

This study conducted a stratify and analyze HRQL differences between AS and SLF patients in a low-prevalence schistosomiasis setting in Jiangxi Province, China. By integrating regional economic and epidemiological data, this work explores the association between HRQL and socioeconomic contextual factors. The significant HRQL impairment observed in this study suggests that in areas with low prevalence, the extent of long-term health-related quality of life damage in patients may not be fully recognized by current strategies primarily focused on transmission control. Combining disease control measures with HRQL-focused interventions—such as integrating mental health support and economic assistance—can mitigate the enduring damage caused by this neglected tropical disease. To align with the World Health Organization's schistosomiasis elimination goals [20], multidimensional strategies are imperative. Future efforts must prioritize equitable healthcare access, holistic rehabilitation, and multisectoral collaboration to achieve comprehensive health

Admin-O
删除: is the first to

Admin-O
删除:

Admin-O
删除: addresses critical research gaps in disease-stage-specific interventions and socioeconomic impact mechanisms

Admin-O
删除: Our findings suggest that HRQL impairments in low-prevalence schistosomiasis settings may be underestimated, as current strategies prioritize transmission control over long-term patient well-being.

4. Regarding the statement that "specific interventions can alleviate lasting damage", we will modify the "can" in the sentence "Combining disease control measures with HRQL-focused interventions—such as integrating mental health support and economic assistance—**can** mitigate the enduring damage caused by this neglected tropical disease." to "may help".

The Limitations section should be strengthened materially. Beyond general self-report concerns, it should avoid framing SF-36 limitations primarily as "recall bias" and instead emphasize reporting/response bias (e.g., social desirability, interviewer effects, and differential interpretation of items across groups, particularly in older and rural populations). It should also explicitly address: (i) cross-sectional inference (no temporality), (ii) representativeness and potential selection bias, (iii) the contextual/area-level nature and dichotomisation of socioeconomic indicators (risk of ecological fallacy and information loss), (iv) stepwise/backward model selection and multiple testing, (v) potential clustering by geographic unit, and (vi) SF-36 scoring choices (PCS/MCS computation method) and ceiling effects in RP/RE. The proposed future directions (longitudinal, comparative, mixed-methods) are appropriate, but should be explicitly linked to these specific limitations.

Response: Thank you very much for your extremely profound and important supplementary comments on the "Limitations" section of this article. The issues you pointed out, particularly regarding the complexity of reporting bias, the fundamental limitations of cross-sectional design in causal inference, the risk of ecological fallacy with region-level variables, potential biases in statistical methods, and specific limitations of the SF-36 scale, are all key to enhancing the rigor of this study's analysis and the accuracy of its conclusions. We fully agree with your comments and will comprehensively and substantially rewrite and expand the 'Limitations' section to more comprehensively and

honestly reflect the shortcomings of this study. Specifically:

"*This study has several limitations. First, the cross-sectional design precludes inferences regarding causality or temporality in the observed associations. Additionally, the SF-36 has known methodological considerations, such as the specific algorithm used to compute PCS/MCS scores and potential ceiling effects in dimensions like Role-Physical and Role-Emotional, which may limit sensitivity in certain subgroups. Second, the representativeness of our findings is subject to several constraints. The study's exclusive focus on Jiangxi Province limits its generalizability to regions with distinct epidemiological or cultural contexts. More importantly, the matched case-control design, while enhancing comparability and statistical power for inter-group contrasts, resulted in a sample that does not reflect the true distribution of disease stages in the underlying community. Therefore, the findings are applicable to revealing association patterns but should not be extrapolated to estimate the overall HRQL burden in the endemic population. Furthermore, the exclusion of participants with major chronic comorbidities (e.g., cardiovascular diseases, diabetes) strengthens internal validity but limits generalizability to real-world populations where comorbidities are prevalent, potentially introducing selection bias. Third, the measurement and modelling of key variables have inherent limitations. We did not collect individual-level data on potential confounders such as education, occupation, or healthcare access. The socioeconomic indicators used (P-DIRH, P-GDP) are contextual, area-level variables that were dichotomized, risking ecological fallacy in interpretation and loss of statistical information. Statistically, while we used the Enter method for regression to avoid biases from stepwise selection, the multiple comparisons conducted across analyses increase the risk of Type I error. We also did not account for potential clustering of responses by geographic unit (e.g., county or township), which might affect standard error estimates.*

Therefore, future research should prioritize longitudinal studies to track changes in HRQL following treatment and evaluate intervention effectiveness. Comparative analyses across diverse epidemiological settings (e.g., marshland/lake versus hilly/mountainous regions) could elucidate environmental association on HRQL. Additionally, mixed-methods studies integrating qualitative evidence—such as patient narratives or caregiver perspectives — may uncover unmet psychosocial needs and guide

patient-centered rehabilitation models. Finally, expanding socioeconomic indicators (e.g., household debt, healthcare affordability) would enhance the understanding of structural barriers to health equity."

Reviewer #3:

However, as with the Discussion, some caution is warranted in phrasing. Statements suggesting that combined HRQL-focused interventions can mitigate long-term damage should be framed more explicitly as implications derived from observed associations rather than demonstrated outcomes of this study. Clarifying this would maintain methodological consistency with the cross-sectional design.

Response: Thank you for the profound and important comments on the conclusion section of this paper. We fully agree. Additionally, we have made minor adjustments to the wording of this text. The specific modifications are as follows:

We will modify the "can" in the sentence "Combining disease control measures with HRQL-focused interventions—such as integrating mental health support and economic assistance—**can** mitigate the enduring damage caused by this neglected tropical disease." to "***may help***".

The Conclusion does not explicitly restate limitations, which is acceptable and common for this section. However, it appropriately avoids overgeneralization by anchoring claims to the Jiangxi Province context and by calling for future efforts rather than asserting definitive solutions.

One potential improvement would be a brief qualifier acknowledging that the findings, while informative, are context-specific. This would further align the Conclusion with the carefully articulated limitations presented earlier in the manuscript.

Response: Thank you for your careful review and valuable comments on this paper. You pointed out that the conclusion section should further elaborate on the contextual specificity of the research findings to be consistent with the limitations detailed in the paper, and we fully agree with this suggestion, which will greatly benefit the rigor and academic expression of this paper. We have comprehensively and substantially revised and expanded the 'Limitations' section to more comprehensively and honestly reflect the shortcomings of this study. Specifically:

"*This study has several limitations. First, the cross-sectional design precludes inferences regarding causality or temporality in the observed associations.*

Additionally, the SF-36 has known methodological considerations, such as the specific algorithm used to compute PCS/MCS scores and potential ceiling effects in dimensions like Role-Physical and Role-Emotional, which may limit sensitivity in certain subgroups. Second, the representativeness of our findings is subject to several constraints. The study’s exclusive focus on Jiangxi Province limits its generalizability to regions with distinct epidemiological or cultural contexts. More importantly, the matched case-control design, while enhancing comparability and statistical power for inter-group contrasts, resulted in a sample that does not reflect the true distribution of disease stages in the underlying community. Therefore, the findings are applicable to revealing association patterns but should not be extrapolated to estimate the overall HRQL burden in the endemic population. Furthermore, the exclusion of participants with major chronic comorbidities (e.g., cardiovascular diseases, diabetes) strengthens internal validity but limits generalizability to real-world populations where comorbidities are prevalent, potentially introducing selection bias. Third, the measurement and modelling of key variables have inherent limitations. We did not collect individual-level data on potential confounders such as education, occupation, or healthcare access. The socioeconomic indicators used (P-DIRH, P-GDP) are contextual, area-level variables that were dichotomized, risking ecological fallacy in interpretation and loss of statistical information. Statistically, while we used the Enter method for regression to avoid biases from stepwise selection, the multiple comparisons conducted across analyses increase the risk of Type I error. We also did not account for potential clustering of responses by geographic unit (e.g., county or township), which might affect standard error estimates.

Therefore, future research should prioritize longitudinal studies to track changes in HRQL following treatment and evaluate intervention effectiveness. Comparative analyses across diverse epidemiological settings (e.g., marshland/lake versus hilly/mountainous regions) could elucidate environmental association on HRQL. Additionally, mixed-methods studies integrating qualitative evidence—such as patient narratives or caregiver perspectives—may uncover unmet psychosocial needs and guide patient-centered rehabilitation models. Finally, expanding socioeconomic indicators (e.g., household debt, healthcare affordability) would enhance the understanding of structural barriers to health equity."
