# Supplementary material for: Assessing Health-Related Quality of life and socioeconomic impacts in low-endemic schistosomiasis: A study from Rural China
Source: PLoS Negl Trop Dis. 2026 Jul 16;20(7):e0014544. doi: 10.1371/journal.pntd.0014544 (PMC13374899; doi:10.1371/journal.pntd.0014544)
Supplement: Attachment — Submitted filename: Response_to_Reviewers_auresp_2.pdf [file pntd.0014544.s004.pdf]

Dear Reviewers:

Thank you for your interest in our manuscript entitled “Assessing Health-Related Quality of Life and Socioeconomic Impacts in Low-Endemic Schistosomiasis: A Study from Rural China”. We have studies comments carefully and have made correction which we hope meet the final approval. The alterations in the text are highlighted in the paper as requested. The main corrections in the paper and the responses to the reviewer’s comments are as following:

Methods

Reviewer #2:

Several key design sentences remain poorly formulated and should be rewritten for scientific clarity:

- “First, all…counties…that cover AS patients…” (unclear selection logic; population description not fully reproducible)
- “Take the AS group served as the baseline cohort…” (grammatically incorrect; “cohort” is misleading in this cross-sectional matched design)
- “try to choose the same township” is not operational; should be described as a rule (township matching prioritized; explicit fallback strategy; success rate if available).

Response:

Comment 1:

We agree that the original description was ambiguous. We have now clarified the sampling logic by explicitly stating that we first identified all counties in Jiangxi Province with a history of schistosomiasis japonica endemicity, and then selected those counties that had at least one registered advanced schistosomiasis (AS) patient according to the official provincial patient registry. The revised text now reads:

“First, from the historical schistosomiasis-endemic counties in Jiangxi Province, we selected all counties that had at least one registered AS patient on the official provincial patient list (Jiangxi Provincial Institute of Parasitic Diseases registry, updated to December 2023).”

This specification ensures full reproducibility.

Comment 2:

We apologize for this grammatical error and the inappropriate use of the term “cohort.” We have corrected the grammar and replaced “cohort” with more accurate terminology. Since our study is cross-sectional with a matched case-control design, we now refer to the AS group as the “reference group” or

“index group” rather than a “cohort.” The revised sentence reads:

“The AS group served as the reference (index) group, while the SLF and HP groups were matched at a 1:1:1 ratio.”

Additionally, we have added a clarifying sentence in the study design paragraph to emphasize that this matching approach is intended to maximize comparability for cross-sectional comparisons and should not be misinterpreted as a longitudinal cohort study.

Comment 3:

We agree that “try to choose” is not an operational rule. We have now specified a hierarchical matching protocol: township-level matching was prioritized; if no eligible control was available within the same township after screening all potential candidates, matching was performed at the county level. We have also provided the success rate of township-level matching based on our implementation. The revised text now reads:

“Matching was performed using a hierarchical rule: (i) priority was given to matching within the same township; (ii) if no eligible control participant was identified in the same township after complete screening of the candidate pool, matching was then performed within the same county but outside the index township. Using this protocol, township-level matching was achieved for 91.3% (807/884) of AS participants. For the remaining 8.7% (77/884), county-level matching was applied. All other matching criteria (gender, age ±5 years) remained unchanged.”

We believe these revisions substantially improve the scientific clarity, transparency, and reproducibility of our methods. We thank the reviewer again for their valuable input.

Sample size estimation:

A power rationale is provided, but the formula corresponds to a two-group mean comparison while the objective is framed as three groups. Authors should state explicitly whether powering was based on the smallest pairwise comparison (and which), or provide a multi-group (ANOVA) justification.

Response:

We thank the reviewer for this careful and methodologically important observation. We agree that the original description was insufficiently explicit regarding how the two-group formula supports a three-group study design.

In our study, although three groups (AS, SLF, HP) are included, the **primary analytical framework is a matched cross-sectional comparison**, with **AS as the reference (case) group** and **SLF and HP as two separate control groups**, matched 1:1:1. The key comparisons of interest are:

- AS vs. HP (largest expected difference)
- AS vs. SLF (smaller expected difference, thus the most conservative)

Therefore, we **powered the study based on the smallest pairwise comparison (AS *vs.* SLF)**, assuming a minimum clinically important difference of 10 points in PCS and a standard deviation of 20. Using the standard two-group formula, we obtained approximately 336 participants per group. To maintain a balanced design and to ensure adequate power for both pairwise comparisons, we set **336 per group** as the target, resulting in **1008 total participants**.

Importantly, this sample size also provides sufficient power for the **Kruskal–Wallis H test** (the nonparametric equivalent of one-way ANOVA), which we used for three-group comparisons. Given the asymptotic relative efficiency of the Kruskal–Wallis test relative to ANOVA (approximately 0.95 for normal distributions), a sample size of 336 per group is more than adequate to detect overall differences among the three groups under the same effect size assumptions.

We have now **revised the manuscript** (in the Sample size estimation subsection) to explicitly state:

- That the power calculation was based on the **smallest pairwise comparison (AS vs. SLF)**;
- That the two-group formula was used appropriately given the matched cross-sectional comparison design;
- That the three-group nonparametric comparison is also well-powered.

We believe this revision addresses the reviewer's concern clearly and strengthens the methodological transparency of the manuscript.

Thank you again for this valuable input.

SF-36 reliability paragraph is overly detailed: Long lists of Cronbach's alpha and test–retest ranges are distracting unless the paper is psychometric; this can be shortened to a single sentence with key citations.

Response:

We thank the reviewer for this constructive and practical suggestion. We agree that the original paragraph containing detailed Cronbach's alpha and test-retest reliability coefficients was overly detailed for the scope of our study, which is not a psychometric validation paper.

Accordingly, we have substantially condensed the description of SF-36 reliability in the revised manuscript (see **Methods → Assessment Instrument Scaling**). The revised version now reads:

"The Chinese version of the SF-36, translated and validated by scholars from Zhejiang University [23], has demonstrated high reliability and validity in both general and schistosomiasis patient populations [19,23]."

This revision retains the essential information about the instrument's psychometric adequacy while removing distracting numerical details, allowing readers to focus on the main findings of our epidemiological study. The key supporting citations ([23] for general population validation and [19] for schistosomiasis-specific application) are preserved for readers who wish to explore the psychometric properties further.

We appreciate the reviewer's attention to manuscript conciseness and focus.

Scoring description (critical, still unresolved): The revised text describes norm-based weighted PCS/MCS, but elsewhere still states PCS/MCS are means of four domains. These cannot both be true.

Response:

We sincerely thank the reviewer for identifying this critical inconsistency in our manuscript. The reviewer is absolutely correct that these two descriptions are mutually exclusive, and this discrepancy could cause serious confusion for readers.

After careful re-examination of our methodology and the standard SF-36 scoring algorithms, we confirm that the **correct approach is the norm-based weighted scoring method** as originally described in the validated Chinese version of the SF-36 (Li et al., 2003; also cited as reference #23 in our manuscript). In this method, the Physical Component Summary (PCS) and Mental Component Summary (MCS) scores are not simple arithmetic means of the four domain scores. Instead, they are calculated using a weighted algorithm derived from factor analysis, with weights calibrated to the Chinese general population norm, producing T-scores that allow cross-study

comparisons.

The erroneous statement that "*PCS and MCS scores were calculated as the mean of their respective four dimension scores*" has been **removed** from the revised manuscript. We have also carefully reviewed the entire manuscript to ensure no other statements contradict the norm-based weighted scoring method. The following sections have been corrected:

- **Methods – Assessment Instrument Scaling**: The incorrect sentence has been deleted, and the norm-based weighted scoring procedure is now described accurately and consistently.
- **Results – Overall assessment of quality of life**: The reporting of median scores remains unchanged, as these are descriptive statistics of the calculated PCS/MCS values (which were correctly generated using the norm-based weighted method). No statement implying simple averaging appears in this section.
- **Discussion**: We have clarified that the SF-36's discriminatory power observed in our study is based on the norm-based weighted scoring. However, **we have added a sentence to make it clear that this study uses a weighting method based on Chinese normative data**: *"It should be noted that the PCS and MCS scores used in this study were derived from the norm-based weighted scoring algorithm recommended for the Chinese version of the SF-36, which enhances comparability with other studies using the same standardized approach [23]."*

We have also verified that our actual data analysis (performed using SPSS with the official SF-36 scoring syntax for the Chinese population) consistently applied the norm-based weighted method. The reported median PCS and MCS values (e.g., AS group: 71.30 and 71.95) are therefore valid and correctly derived.

We apologize for this oversight and thank the reviewer again for their meticulous reading, which has substantially improved the methodological clarity of our manuscript.

Contextual variables: The clarification that P-DIRH and P-GDP are county-level contextual variables is appropriate, but currently inserted in a staccato way.

Response:

We fully agree with your observation. In the original manuscript, the

clarification that P-DIRH and P-GDP are county-level contextual variables was placed as an isolated note within parentheses, which disrupted the reading flow. To address this, we have now **integrated this clarification smoothly into the main text of the Methods section ("Classification by socio-economic and demographic characteristics")**, rather than presenting it as a fragmented note. Specifically, we have rewritten the relevant paragraph to first introduce these variables as area-level contextual indicators, then explicitly state their ecological (county-level) nature, and finally explain how they were dichotomized. This revision improves the logical continuity and readability of the methods.

We have also checked the entire manuscript to ensure no other "staccato" insertions remain. The revised text is highlighted using **track changes** (in bold or underlined format) in the revised manuscript.

We greatly appreciate your guidance and believe the revised version is substantially improved.

Before revision (original text): *Socio-economics variables: The per-capita disposable income (P-DIRH) and per-capita gross domestic product (P-GDP) data used were sourced from the "Jiangxi Statistical Yearbook (2022)" at the county level. We compared the P-DIRH and P-GDP values of the surveyed counties with the provincial average. Those higher than the average were categorized as "High", and those lower than the average were categorized as "Low". Note: P-DIRH and P-GDP are county-level contextual variables, not individual household measures.*

Revised version (amendments shown in bold): *Socio-economics variables: The per-capita disposable income of rural households (P-DIRH) and per-capita gross domestic product (P-GDP) were extracted from the "Jiangxi Statistical Yearbook (2022)" at the county level.* ***These are county-level contextual variables****, reflecting the broader economic environment of participants' residential areas* ***rather than individual household economic status****. For each indicator, we compared the county-level value against the provincial average: counties with values above the average were categorized as "High", and those below as "Low".*

Elimination criteria text is redundant/unclear: The "5 consecutive years after interruption" clause should be streamlined and operationalized (how classification was applied to counties in this

dataset and for which time period).

Response:

We fully agree with the reviewer that the original description of the elimination criteria was redundant and lacked operational clarity. To address this, we have substantially revised the "**Endemic status of schistosomiasis (ESS)**" subsection in the **Methods** section as follows:

1. **Streamlining the criteria**: We have separated the definitions of "transmission interruption" and "elimination" into two distinct bullet points, removing repetitive wording and clearly indicating that elimination requires **an additional five consecutive years** after interruption has been achieved.

2. **Operationalization for this dataset**: We now explicitly state that:

- The classification was based on the **latest official endemic status** certified by the **Health Commission of Jiangxi Provincial**.
- The reference time point is **December 31, 2023** (the year prior to data collection).
- No county in our dataset remained at the "transmission control" or "prevention" stage; all were either at "transmission interruption" or "elimination".
- **A binary variable** (elimination vs. interruption) was used in all regression models.

3. **Consistency with results**: The revised definition aligns with the interpretation of the ESS variable in Table 1 and the associated findings (e.g., positive association between elimination status and HRQL in the AS group).

We believe these revisions make the criteria both clear and directly applicable to the data presented. The changes have been highlighted in the revised manuscript.

We thank the reviewer again for this important clarification, which has significantly improved the transparency and reproducibility of our study.

Reviewer #3:

Study Design Terminology: The manuscript continues to refer to both a cross-sectional design and a case-control approach. Based on the description provided, the study is best characterized as a matched cross-sectional comparative study. Consistent terminology would improve methodological clarity.

Response:

We thank the reviewer for this crucial methodological observation. We fully agree that using both “cross-sectional” and “case-control” terminology in the same manuscript is confusing and inconsistent with the actual study design.

Revisions made:

1. **Terminology unification**: We have replaced all instances of “case-control” (or “case-control study approach”) with “matched cross-sectional comparative study” or “matched cross-sectional design” throughout the manuscript. Specifically:

- In the **Methods → Participants section**, the sentence “we adopted a case-control study approach” has been deleted and replaced with “This matched cross-sectional design is intended to maximize comparability among the three groups...”
- In the **Sample size estimation section**, “the core comparative design is case–control” has been changed to “the core comparative design is a matched cross-sectional comparison”.
- In the **Limitations** section, “the matched case-control design” has been changed to “the matched cross-sectional design”.

2. **Consistency check**: We have verified that the abstract, keywords, and all other sections now consistently refer to a “cross-sectional study” or “matched cross-sectional comparative study”. No contradictory terminology remains.

3. **Clarification added**: In the Participants section, we have explicitly stated that this is a matched design intended to maximize comparability among the three groups for cross-sectional analysis of HRQL, and that it does not imply a longitudinal cohort structure or case-control sampling from a source population.

We believe these revisions eliminate the previous terminological ambiguity and accurately reflect the study as a matched cross-sectional comparative study. All changes have been highlighted using track changes in the revised manuscript.

We greatly appreciate the reviewer’s attention to methodological precision, which has improved the clarity of our manuscript.

Handling of Matching in Analysis: While matching by age, gender, and residence is described in detail, it remains unclear how this was accounted for analytically. The authors should clarify whether matching was used solely to enhance comparability or incorporated into the statistical

analysis.

Response:

We thank the reviewer for raising this important methodological point. We agree that the analytical handling of matching needs to be explicitly clarified.

In our study, matching was used exclusively as a design-stage strategy to enhance baseline comparability among the three groups (AS, SLF, HP).

We have now explicitly stated this in the Participants subsection of the Methods (see highlighted revision). As follows:

"To avoid misinterpretation, we note that this matched design is intended to maximize comparability among the three groups for cross-sectional analysis of health-related quality of life; it does not imply a longitudinal follow-up (cohort) structure, nor should the results be used to infer population prevalence or absolute HRQL levels in the source population."

We believe these revisions address the reviewer's concern and improve the transparency of our analytical approach.

PCS/MCS Computation: There is an internal inconsistency in the description of composite score calculation. The manuscript states that PCS and MCS are weighted summaries derived using norm-based coefficients, but later describes them as simple means of the four component dimensions. This should be clarified to ensure reproducibility.

Response:

We sincerely thank the reviewer for identifying this critical inconsistency in our manuscript (**This is a common issue raised by Reviewer #2**). The reviewer is absolutely correct that these two descriptions are mutually exclusive, and this discrepancy could cause serious confusion for readers.

After careful re-examination of our methodology and the standard SF-36 scoring algorithms, we confirm that the **correct approach is the norm-based weighted scoring method** as originally described in the validated Chinese version of the SF-36 (Li et al., 2003; also cited as reference #23 in our manuscript). In this method, the Physical Component Summary (PCS) and Mental Component Summary (MCS) scores are not simple arithmetic means of the four domain scores. Instead, they are calculated using a weighted algorithm derived from factor analysis, with weights calibrated to the Chinese general population norm, producing T-scores that allow cross-study comparisons.

The erroneous statement that "*PCS and MCS scores were calculated as the mean of their respective four dimension scores*" has been **removed** from the revised manuscript. We have also carefully reviewed the entire manuscript to ensure no other statements contradict the norm-based weighted scoring method. The following sections have been corrected:

- **Methods – Assessment Instrument Scaling**: The incorrect sentence has been deleted, and the norm-based weighted scoring procedure is now described accurately and consistently.
- **Results – Overall assessment of quality of life**: The reporting of median scores remains unchanged, as these are descriptive statistics of the calculated PCS/MCS values (which were correctly generated using the norm-based weighted method). No statement implying simple averaging appears in this section.
- **Discussion**: We have clarified that the SF-36's discriminatory power observed in our study is based on the norm-based weighted scoring. **We have added a sentence to make it clear that this study uses a weighting method based on Chinese normative data**: *"It should be noted that the PCS and MCS scores used in this study were derived from the norm-based weighted scoring algorithm recommended for the Chinese version of the SF-36, which enhances comparability with other studies using the same standardized approach [23]."*

We have also verified that our actual data analysis (performed using SPSS with the official SF-36 scoring syntax for the Chinese population) consistently applied the norm-based weighted method. The reported median PCS and MCS values (e.g., AS group: 71.30 and 71.95) are therefore valid and correctly derived.

We apologize for this oversight and thank the reviewer again for their meticulous reading, which has substantially improved the methodological clarity of our manuscript.

Regression Assumptions: Although linear regression is used for PCS and MCS outcomes, the manuscript does not report whether regression assumptions (e.g., normality of residuals, homoscedasticity, multicollinearity) were assessed. Brief clarification would strengthen the statistical rigor.

Response:

We fully agree with the reviewer that transparent reporting of regression diagnostics is essential for statistical rigor. In the revised manuscript, we have added a dedicated paragraph in the **Statistical analysis** subsection (Methods) that explicitly describes the assessment of key linear regression assumptions. Specifically, we now report:

1. **Normality of residuals**: Assessed using the Shapiro–Wilk test and Q-Q plots. While the Shapiro–Wilk test indicated slight deviations ($P < 0.05$) due to our large sample size, Q-Q plots showed residuals to be approximately normally distributed without severe skewness. We also note that the central limit theorem supports the robustness of the estimates given n = 884 per group.
2. **Homoscedasticity**: Examined via plots of standardized residuals against predicted values. No clear funnel patterns or systematic variance inflation were observed, indicating that the constant variance assumption was reasonably satisfied.
3. **Multicollinearity**: Evaluated using variance inflation factor (VIF) and tolerance. All VIF values were below 2.0 (range: 1.03–1.89) and tolerance values exceeded 0.5, confirming no substantial multicollinearity among the independent variables.
4. **Independence of residuals**: Confirmed by the study design (independent observations) and supported by Durbin–Watson statistics ranging from 1.85 to 2.10, indicating no significant autocorrelation.

We have incorporated these diagnostic results into the revised manuscript (see **Methods: Statistical analysis**). We believe this addition significantly strengthens the statistical transparency and rigor of our work.

We thank the reviewer for this constructive suggestion, which has improved the quality of our manuscript.

Results

Reviewer #3:

Regression assumption checks (e.g., residual normality, homoscedasticity, multicollinearity) are still not reported.

Response:

We apologize for the omission. We have now added a dedicated subsection within the Statistical analysis section of the Methods (pages 10–11 in the

revised manuscript) that systematically reports regression assumption checks for all models (AS and SLF groups, separately for PCS and MCS outcomes). Specifically:

1. **Multicollinearity**: We calculated variance inflation factors (VIF) for all independent variables. VIF values ranged from 1.03 to 1.89 (all < 2.0), indicating no problematic collinearity.
2. **Homoscedasticity**: We visually inspected scatterplots of standardized residuals versus predicted values. No obvious funnel patterns or systematic heteroscedasticity were observed.
3. **Normality of residuals**: We examined Q‑Q plots and performed the Shapiro–Wilk test. Although the Shapiro–Wilk test was significant ($P < 0.05$) for some models due to the large sample size ($n = 844$ per group), the Q‑Q plots showed that residuals approximated a normal distribution with only minor deviations at the tails. Given the robustness of linear regression to moderate non‑normality with large samples, the models are considered adequately specified.
4. **Independence of residuals**: Based on the study design (no clustering, repeated measures, or spatial autocorrelation), we assumed independence; no obvious dependency patterns were detected.

These diagnostic checks support the validity of the reported regression results. The revised text also clarifies that we used the Enter method for variable selection and how we interpreted standardized beta coefficients.

We have added the following to the end of the “Analysis of factors affecting HRQL” paragraph:

“Regression diagnostics (multicollinearity, homoscedasticity, and residual normality) confirmed that the linear models met key assumptions (see Methods for details).”

We believe these additions fully address the reviewer’s concern and improve the transparency of our statistical reporting. The changes are clearly highlighted in the revised manuscript.

We thank the reviewer again for this important methodological comment.

Reference categories for regression variables are not explicitly stated in Table 1.

Response:

We fully agree with the reviewer that specifying reference categories is

essential for the correct interpretation of regression results. Accordingly, we have revised Table 1 by adding a dedicated footnote that explicitly lists the reference category for each categorical variable included in the multivariable linear regression models.

Specifically, the following reference categories have now been clearly stated:

- Gender: Male (reference)
- Age: 20–59 years (reference)
- P-DIRH: High (reference)
- P-GDP: High (reference)
- TSE: Marshland/lake regions (reference)
- ESS: Transmission interruption (reference)

These revisions ensure that readers can correctly understand the direction and magnitude of each association without ambiguity. The changes have been highlighted using track changes in the revised manuscript.

We thank the reviewer again for this valuable suggestion, which has strengthened the methodological rigor of our paper.

Occasional interpretive statements in the Results text could be neutralized.

Response:

We fully agree with the reviewer that the Results section should present findings without interpretive or inferential language. We have carefully reviewed the entire Results section and revised the following passages to ensure purely descriptive reporting:

1. **Original (interpretive)**: “These results demonstrate a progressive decline in HRQL from healthy individuals to SLF and AS patients.”
   Revised (neutral): “These results show lower HRQL scores in the SLF group compared to the HP group, and lowest scores in the AS group among the three groups.”
2. **Original (interpretive)**: “Pairwise comparisons demonstrated that…”
   Revised (neutral): “Pairwise comparisons showed that…”
3. **Original (interpretive)**: “Age ≥60 years exhibited the strongest negative association … but the degree of control that had reached the elimination stage significantly co-occurred with …”
   Revised (neutral): “Age ≥60 years showed the largest negative standardized coefficients … Elimination status was associated with higher

PCS and MCS scores in this group."

4. **Original (interpretive)**: "Age ≥60 years remained the most strongly associated with lower HRQL scores, with the largest negative coefficients …"

   Revised (neutral): "Age ≥60 years showed the largest negative standardized coefficients for PCS and MCS in the SLF group."

All revisions have been highlighted using track changes in the revised manuscript. We believe these changes make the Results section strictly descriptive and free of interpretive statements, in line with the journal's standards.

We thank the reviewer again for this valuable suggestion, which has improved the rigor and clarity of our manuscript.

The unusually wide MCS interquartile range in the HP group may require verification.

Response:

We have re-examined the original dataset and confirmed that the MCS values for the HP group are correctly reported as follows:

Median = 80.80, first quartile (Q1) = **67.50**, third quartile (Q3) = 89.10.

We thank the reviewer for prompting this verification, which has improved the clarity and rigor of our manuscript.

Conclusions

Reviewer #2:

Limitations are more comprehensive than in the original version and appropriately acknowledge key constraints of cross-sectional inference and contextual (county-level) predictors. The section would benefit from clearer integration (less "patchwork" insertion).

Response:

We fully agree with the reviewer that the previous version of the Limitations section, while comprehensive, read as a series of loosely connected points. To address this, we have substantially reorganized and streamlined the section as follows:

1. **Thematic structuring**: We have grouped the limitations into four clear thematic subsections: Study design constraints, Representativeness and generalizability, Measurement and modeling limitations, and Statistical

considerations. This structure replaces the original “First, Second, Third” enumeration and improves logical flow.

2. **Integrated narrative**: Rather than inserting isolated sentences (e.g., “Additionally, the SF-36 has known methodological considerations…”), we have woven related points together. For example, the SF-36’s ceiling effects and norm-based scoring are now discussed within the “Study design constraints” subsection as a coherent paragraph.

3. **Reduced redundancy**: We have removed repetitive phrasing and merged overlapping points (e.g., the matched design’s impact on generalizability is now stated once, clearly, in the “Representativeness” subsection).

4. **Clearer signaling of future directions**: The “Future directions” paragraph now directly follows from the limitations discussed, creating a smoother transition than the previously standalone “Therefore, future research should…” sentence.

We believe these revisions make the Limitations section more readable, logically organized, and professionally integrated—while preserving all the important methodological caveats you correctly identified. All changes have been highlighted using track changes in the revised manuscript.

Revised (strengthened and reorganised into four coherent paragraphs with fewer patchwork insertions):

*“**Limitations***

Several methodological limitations should be considered when interpreting the findings of this study. These limitations pertain to study design, sample representativeness, measurement of key variables, and statistical considerations.

Study design constraints. The cross-sectional design precludes any causal or temporal inferences regarding the observed associations between disease stage, socioeconomic factors, and HRQL. Additionally, the SF-36 instrument has inherent methodological considerations: the norm-based algorithm used to compute PCS and MCS scores, while validated in Chinese populations, may not fully capture culturally specific dimensions of well-being; ceiling effects in the Role-Physical and Role-Emotional dimensions (as evident in our data) may reduce sensitivity in discriminating higher-functioning subgroups.

Representativeness and generalizability. Our findings are subject to several sampling constraints. First, the study was conducted exclusively in Jiangxi

Province, which limits generalizability to regions with different epidemiological profiles (e.g., hilly vs. marshland areas) or healthcare infrastructures. Second, the matched case-control design—while enhancing comparability and statistical power for inter-group contrasts—resulted in a sample that does not reflect the true distribution of AS, SLF, and HP in the source community. Thus, our results should be interpreted as revealing associational patterns rather than estimating population prevalence or absolute HRQL burden. Third, we excluded participants with major chronic comorbidities (cardiovascular disease, diabetes, renal disorders, hepatitis, or cirrhosis) to strengthen internal validity; this necessarily limits generalizability to real-world populations where multimorbidity is common and may introduce selection bias.

Measurement and modeling limitations. We did not collect individual-level data on potential confounders such as education, occupation, or healthcare access. The socioeconomic indicators (P-DIRH, P-GDP) are county-level contextual variables, and their dichotomization (high/low relative to provincial average) risks ecological fallacy when interpreting associations at the individual level and may result in loss of statistical information. Furthermore, our regression models did not account for potential clustering of responses by geographic unit (e.g., county or township), which could affect standard error estimates.

Statistical considerations. Although we used the Enter method to avoid variable selection bias inherent in stepwise regression, the multiple comparisons conducted across different groups and outcomes increase the risk of Type I error. Readers should interpret the reported p-values with this in mind.

Future directions. To address these limitations, future research should prioritize longitudinal studies to track HRQL changes following treatment and evaluate intervention effectiveness. Comparative analyses across diverse epidemiological settings (e.g., marshland/lake versus hilly/mountainous regions) could elucidate environmental influences on HRQL. Mixed-methods studies integrating qualitative evidence—such as patient narratives or caregiver perspectives—may uncover unmet psychosocial needs and guide patient-centered rehabilitation models. Finally, expanding socioeconomic indicators (e.g., household debt, healthcare affordability) would enhance understanding of structural barriers to health equity.”

We thank you again for your insightful review, which has significantly improved

the clarity and rigor of our paper.

The opening paragraph largely reiterates background already covered in the Introduction; it can be shortened to focus on interpretation of the study's main findings and contributions.

Response:

We fully agree with the reviewer that the original opening paragraph of the Discussion unnecessarily repeated background information already presented in the Introduction. To address this, we have substantially revised this paragraph as follows:

1. **Removed redundant background**: We deleted sentences that restated the general public health challenge of schistosomiasis, China's control progress, and the generic research gaps (e.g., "Schistosomiasis remains a major public health challenge…", "Previous studies have predominantly focused on high-transmission settings…"). These points are adequately covered in the Introduction.

2. **Focused on interpretation of main findings**: The revised paragraph now opens directly with three core findings from our study, presented as bullet points for clarity:

- Stepwise decline in HRQL across AS, SLF, and HP groups, with specific dimensions most affected;
- Age ≥60 years as the strongest associated factor;
- Positive association between elimination-status areas and HRQL in AS patients.

3. **Highlighted contributions**: We then explicitly state the three key contributions of this study (stage-specific profiles, socioeconomic integration, methodological rigor), which align with the aims stated in the Introduction. This restructuring allows readers to immediately grasp what this study uniquely adds to the literature.

4. **Maintained essential caveat**: We retained the opening sentence regarding the cross-sectional design ("observed associations should be interpreted as correlational"), as this is a critical methodological limitation that should be stated early in the Discussion.

We believe the revised opening paragraph is now concise, focused, and directly communicates the study's main findings and contributions without repeating background material. The changes have been highlighted in the

revised manuscript.

Before revision (original text):

"*Schistosomiasis remains a major public health challenge in low-income regions, despite China's significant progress in reducing transmission through targeted control programs [3,4,27]. However, HRQL among individuals in low-endemic areas, particularly those with AS and SLF, remains poorly characterized. Previous studies have predominantly focused on high-transmission settings or isolated clinical outcomes [9,10,12,15], overlooking the complex interplay of disease severity, socioeconomic disparities, and geographic factors in shaping HRQL. For instance, while King et al. (2005) systematically reviewed the long-term disability burden of schistosomiasis [2], few studies have stratified outcomes by comorbidities such as AS and SLF. Similarly, socioeconomic correlates—critical in rural China, where poverty exacerbates disease burden—have rarely been incorporated into HRQL analyses [20]. To address these gaps, this study employed the SF-36 scale alongside socioeconomic and geographic variables to evaluate HRQL in a low-prevalence area of Jiangxi Province, China.*"

Revised version:

"This study provides three main findings regarding HRQL in low-endemic schistosomiasis. First, HRQL scores followed a stepwise decline from healthy controls to SLF to AS patients, with AS patients showing the most severe impairments in physical functioning, vitality, social function, and general health. Second, age ⩾60 years was the strongest factor associated with lower HRQL, outweighing the impact of economic indicators. Third, residing in areas that had achieved official elimination status was positively associated with HRQL among AS patients, suggesting potential benefits from sustained control programs. These findings advance the field by: (i) providing stage-specific HRQL profiles that distinguish AS from SLF in a low-endemic setting, (ii) integrating area-level economic variables to reveal structural inequalities, and (iii) demonstrating the utility of the SF-36 for cross-study comparability in elimination-phase surveillance. By stratifying disease severity and incorporating socioeconomic contextual factors, this study offers evidence to inform stage-targeted rehabilitation and multisectoral policies aligned with the WHO 2030 elimination roadmap."

We thank the reviewer for this valuable suggestion, which has significantly

improved the clarity and impact of our Discussion section.

Justification of instrument choice (SF-36 vs EQ-5D-5L) is more appropriate in the Methods; the example involving rural hypertensive patients is tangential to schistosomiasis and can be removed or replaced with schistosomiasis-specific measurement considerations.

Response:

We fully agree with the reviewer's suggestion. We have made the following two revisions accordingly:

1. We have relocated the comparison between SF-36 and EQ-5D-5L, along with the rationale for choosing SF-36, to the "Assessment Instrument Scaling" subsection of the Methods.

2. We have removed the sentence referencing rural hypertensive patients (Wang et al., 2008) and replaced it with a schistosomiasis-specific example:

"For example, studies in Chinese schistosomiasis patients have shown that the SF-36's domain structure is sensitive to liver fibrosis severity, with general health (GH) and vitality (VT) scores correlating with clinical markers of hepatic dysfunction [19,24], underscoring the importance of disease-specific contextual interpretation."

We believe these changes enhance the clarity, relevance, and methodological rigor of the manuscript. Thank you for guiding us to improve our work.

Some statements introduce unmeasured mechanisms as if established (e.g., low P-GDP "leading to delayed interventions" due to limited healthcare resources). Since healthcare access and treatment timing were not assessed, these should be reframed explicitly as hypotheses.

Response:

We fully agree with the reviewer's point. In the original manuscript, we inadvertently used language that could be interpreted as asserting causal mechanisms (e.g., "may stem from inadequate healthcare infrastructure and delayed treatment-seeking behaviors") without having directly measured those intermediate variables.

To address this, we have revised the relevant paragraph in the Discussion section as follows:

1. **Reframed as explicit hypotheses**: We now lead with "We hypothesize that this observed association could be explained by…" and use cautious, speculative language throughout ("it is plausible that…", "may face…",

“potentially…”, “might compound…”).

2. **Acknowledged unmeasured factors**: We explicitly state that our study did not collect individual-level data on healthcare access, treatment timing, or health system characteristics, and therefore these proposed mechanisms remain speculative.

We believe this revision appropriately tempers the claims and aligns with the reviewer’s request to present unmeasured mechanisms as hypotheses rather than established facts. No other similar overstatements were found elsewhere in the manuscript.

Editorial and Data Presentation Modifications?

Reviewer #2:

While several technical revisions were implemented, many changes read as “patchwork” insertions rather than fully integrated edits, resulting in awkward grammar, staccato transitions, and occasional unclear phrasing. I recommend a focused language and flow edit across the manuscript to merge added sentences smoothly, eliminate redundancy, and ensure consistent terminology and scientific phrasing.

Response:

OK, we have now edited the text in response to the issues you raised.
