# Supplementary material for: Assessing Health-Related Quality of life and socioeconomic impacts in low-endemic schistosomiasis: A study from Rural China
Source: PLoS Negl Trop Dis. 2026 Jul 16;20(7):e0014544. doi: 10.1371/journal.pntd.0014544 (PMC13374899; doi:10.1371/journal.pntd.0014544)
Supplement: Attachment — Submitted filename: Response_to_Reviewers_auresp_3.pdf [file pntd.0014544.s005.pdf]

Dear Reviewers:

Thank you for your interest in our manuscript entitled “Assessing Health-Related Quality of Life and Socioeconomic Impacts in Low-Endemic Schistosomiasis: A Study from Rural China”. We have studies comments carefully and have made correction which we hope meet the final approval. The alterations in the text are highlighted in the paper as requested. The main corrections in the paper and the responses to the reviewer’s comments are as following:

Journal Requirements:

1) Your manuscript's sections are not in the correct order. Please amend to the following order: Abstract, Introduction, Results, Discussion, and Methods

Response:

OK, we have made adjustments in accordance with the journal's typesetting requirements.

Methods

Reviewer #2:

Study design: Two nearly identical disclaimers about the matched design appear within one page of each other in the Participants section and should be merged into one. The term "case-sectional study approach" is not a recognized methodological term and should be removed. The term "matched case-control design" reappears in the Limitations section, contradicting the corrected terminology used elsewhere.

Response:

Thank you for your careful reading and constructive comments on our manuscript. We have addressed the specific issues you raised regarding study design terminology. Below is our point-by-point response.

Comment 1: We agree with your observation. We have merged the two separate disclaimers into a single, concise paragraph in the Participants section. In doing so, we have also removed the non-standard term “case-sectional study approach” and replaced it with a clearer description: “we adopted a matched cross-sectional design, setting the AS group as the reference group and constructing the SLF and HP groups through 1:1:1 matching.” The merged disclaimer now appears once and clearly states the intended comparability and the limitations regarding prevalence inference. (Please see the revised Participants section in the Methods.) The changes made in the revision are as follows:

Original content (two separate paragraphs containing non-standard

terminology):

"To avoid misinterpretation, we note that this matched design is intended to maximize comparability among the three groups for cross-sectional analysis of health-related quality of life; it does not imply a longitudinal follow-up (cohort) structure, nor should the results be used to infer population prevalence or absolute HRQL levels in the source population."

"The study aims to compare differences in HRQL among populations at different disease stages (AS, SLF, HP). To this end, we adopted a case-sectional study approach, setting the AS group as the 'case' baseline and constructing the SLF group and HP group through equal matching. This matched cross-sectional design is intended to maximize comparability among the three groups (AS, SLF,HP) to clearly reveal the association between disease stage and HRQL; however, its results should not be used to infer the actual prevalence of AS, SLF, or HP in the source population or the overall average HRQL level."

Revised (combined into a single paragraph; "case-sectional study approach" removed):

"*The study aims to compare differences in HRQL among populations at different disease stages (AS, SLF, HP). To this end, we adopted a matched cross-sectional design, setting the AS group as the reference group and constructing the SLF and HP groups through 1:1:1 matching. This matched design is intended to maximize comparability among the three groups to clearly reveal the association between disease stage and HRQL; it does not imply a longitudinal follow-up (cohort) structure. The results should not be used to infer the actual prevalence of AS, SLF, or HP in the source population, nor the absolute HRQL levels in the general population.*"

Comment 2: Thank you for pointing out this inconsistency. We have revised the **Limitations** section accordingly, changing "matched case-control design" to "matched cross-sectional design" to align with the corrected terminology used throughout the manuscript. (Please see the second bullet point under "Representativeness and generalizability" in the Limitations section.)

We believe these revisions have improved the clarity and methodological rigor of the manuscript. We appreciate your thorough review and hope that the revised version meets your approval.

Sample size: The manuscript states the power calculation was based on AS vs. HP as "the smallest expected pairwise difference" and "most conservative comparison." This is likely incorrect — AS vs. SLF (two disease stages) would be expected to yield the smaller difference compared to AS vs. HP (disease vs. healthy controls), making AS vs. SLF the more conservative basis for power calculation.

Response:

We thank the reviewer for this careful and methodologically important observation. We fully agree that **the comparison between two disease stages (AS vs. SLF) is expected to yield a smaller difference than that between a disease stage and healthy controls (AS vs. HP)**. Therefore, the AS–SLF contrast, rather than AS–HP, should indeed serve as the most conservative basis for sample size estimation.

Accordingly, we have revised the sample size justification in the Methods section as follows:

- We explicitly state that the power calculation is now based on AS vs. SLF as the smallest expected pairwise difference.
- We retain the same parameters ($\sigma = 20$, $\delta = 10$, $\alpha = 0.05$, power = 0.90), which still yield a required sample size of approximately 336 per group.
- To further address the reviewer's concern, we additionally performed a sensitivity calculation using a more conservative $\delta = 7.5$ (assuming an even smaller AS–SLF difference). This increased the required sample size to approximately 590 per group.
- Our final sample of 884 participants per group still exceeds this more stringent requirement, confirming that the study is well-powered even under the conservative AS–SLF assumption.

The specific amendments are as follows:

Original text (incorrect wording):

"Therefore, the sample size was calculated based on the smallest expected pairwise difference, i.e., between AS and HP, to ensure adequate power for the most conservative comparison."

Revised (correct version):

"*Therefore, the sample size was calculated based on the comparison that is likely to yield the smallest expected difference, i.e., between AS and SLF, as this represents the most conservative basis for power calculation.*"

Additional information:

"*To further validate the robustness of the sample size, we recalculated using a more conservative δ = 7.5 (reflecting a potentially smaller AS–SLF difference), which yielded a required sample size of approximately 590 per group. Our actual sample (n = 884 per group) still provides adequate power, confirming the study is well-designed for detecting differences between all three groups.*"

We believe this revision correctly addresses the reviewer's concern and strengthens the methodological transparency of the manuscript. The revised text has been highlighted in the resubmitted file.

We sincerely thank the reviewer for helping us improve the statistical rigor of our work.

Statistical analyses: Table 1 reports N = 844 for some models and N = 884 for others

Response:

We thank the reviewer for their careful reading and for identifying this inconsistency in Table 1. We have rechecked our original dataset and confirmed that the total sample size for each group (AS, SLF, and HP) is 884, and all regression analyses were performed on the full sample of 884 participants per group.

The appearance of "844" in some cells of Table 1 was a typographical error during manuscript preparation. We have now corrected all instances of "844" to "884" in Table 1 for the following models:

- Physical Component Summary (PCS) – AS group: corrected from 844 to 884
- Physical Component Summary (PCS) – SLF group: corrected from 844 to 884
- Mental Component Summary (MCS) – SLF group: corrected from 844 to 884

All other sample size entries in Table 1 (e.g., MCS – AS group: 884) were already correct and remain unchanged. These corrections do not affect any statistical results, conclusions, or other parts of the manuscript.

We apologize for any confusion this error may have caused and appreciate the reviewer's diligence in improving the accuracy of our report.

Results

Reviewer #2:

Gradient claim: The abstract and Discussion repeatedly describe an unqualified gradient "AS < SLF < HP" for both PCS and MCS. This is not fully supported: while all three pairwise comparisons are significant for PCS, the SLF vs. HP comparison for MCS is never reported as significant.

Response:

We sincerely thank the reviewer for this careful and precise observation. We fully agree with the reviewer that our original claim of an unqualified gradient “AS < SLF < HP” for both PCS and MCS is not fully supported by the data. As correctly noted, while the pairwise comparisons for PCS were all significant (AS vs. SLF, AS vs. HP, and SLF vs. HP), the comparison between SLF and HP for MCS was not statistically significant.

To address this issue, we have carefully revised the manuscript to avoid overgeneralization. Specifically, we have:

Abstract (revised): We have replaced the statement “It was observed that the HRQL scores showed a gradually increasing trend (AS < SLF < HP)” with a more precise description. We now explicitly state the significant gradient for PCS. **Revised to** “*It was observed that the HRQL scores showed a gradually increasing trend (AS < SLF < HP) for the physical component summary (PCS).*”

Discussion (revised): We have revised the statement “HRQL scores followed a stepwise decline from healthy controls to SLF to AS patients” to instead read: “For physical health (PCS), scores followed a stepwise decline … For mental health (MCS), while AS patients showed significantly lower scores than both SLF and HP groups, the difference between SLF and HP did not reach statistical significance.”

We thank the editor and reviewer for their continued guidance.

Table 1 — ESS direction reversal: Elimination status (ESS) is positively associated with HRQL in AS patients (β = +0.122 PCS, +0.110 MCS) but negatively associated in SLF patients (MCS: β = -0.057, P = 0.038). This opposite direction finding is reported in the table but never acknowledged or discussed anywhere in the manuscript.

Response:

We sincerely thank you for your careful re-review and for pointing out the critical issue regarding the unreported direction reversal of the ESS association in Table 1. We apologize for this omission in our previous submission. We fully agree with the reviewer that this opposite-direction finding is noteworthy and should not be left undiscussed. We have now added a dedicated paragraph in the Discussion section to explicitly acknowledge and interpret this finding. Specifically, we now:

"*An unexpected yet noteworthy finding emerged from our multivariable regression: the direction of association between ESS and HRQL differed across disease stages. Among AS patients, residing in areas that had officially achieved schistosomiasis elimination was positively associated with both PCS ($\beta = 0.122$, $P < 0.001$) and MCS ($\beta = 0.110$, $P < 0.001$). In contrast, among SLF patients, elimination status was negatively associated with MCS scores ($\beta = -0.057$, $P = 0.038$), with no significant association with PCS. To our knowledge, this opposite-direction finding has not been previously reported and warrants careful interpretation. One plausible explanation involves surveillance intensity and detection bias. In elimination-certified areas, the healthcare system maintains highly sensitive surveillance for schistosomiasis, which may lead to the identification and registration of SLF patients with milder or subclinical symptoms who might otherwise remain undiagnosed in non-elimination areas. The inclusion of these "healthier" SLF patients could paradoxically lower the group's average HRQL if they perceive their newly labeled condition as a source of anxiety or stigma—an effect less likely to occur in AS patients whose severe symptoms are unequivocal and well-recognized. Alternatively, the positive association in AS patients may reflect tangible benefits from targeted social support programs that are often strengthened during the elimination phase (e.g., regular home visits, subsidized medications, rehabilitation services), which disproportionately benefit the most disabled group. A third possibility is residual confounding by unmeasured area-level factors, such as the density of primary care providers, local economic subsidies for chronic patients, or differential health-seeking behaviors. Given the cross-sectional design and the exploratory nature of this subgroup analysis, the opposite associations should be interpreted as hypothesis-generating rather than causal. Future mixed-methods studies incorporating patient interviews and health system audits are needed to elucidate the mechanisms underlying this*

stage-specific heterogeneity."

Fig. 2: The VT dimension is incorrectly labelled "validity" in the figure legend — it should be "vitality."

Response:

We apologize for this typographical error. We have corrected the figure legend in the revised manuscript: the abbreviation "VT" now reads "vitality" instead of "validity". No other changes were made to the figure or its content.

We confirm that this correction has been made in the revised manuscript with track changes. The figure legend now accurately reflects the SF-36 dimension. Thank you again for your valuable feedback.

Table 1 — R^2 values: The large discrepancy between unadjusted and adjusted R^2 values (e.g., 0.281 vs. 0.079 for AS-PCS; 0.208 vs. 0.043 for AS-MCS) is unexpected given the sample size (N ~884) and number of predictors. With these parameters the adjustment penalty should be minimal.

Response:

We thank the reviewers for pointing out this significant data inconsistency. We have re-examined the original statistical output and confirmed that the values for model fit (R^2 / Adj. R^2) in Table 1 were incorrect due to an error during data extraction, in which R values were mistakenly entered into the R^2 column and R^2 values were entered into the Adj. R^2 column. We have revised the values as follows (see the figure below for the statistical values):

AS-PCS：R^2 = 0.079; adjusted R^2 = 0.076

AS-MCS： R^2 = 0.043; adjusted R^2 = 0.040

AS-PCS： R^2 = 0.089; adjusted R^2 = 0.086

AS-MCS： R^2 = 0.064; adjusted R^2 = 0.061

The revised Table 1 is provided in the updated manuscript with the corrected model fit statistics for both AS and SLF groups. We deeply apologize for this oversight and appreciate the reviewer's careful reading.

We believe these corrections fully resolve the concern and thank the reviewer for their valuable feedback.

模型摘要

模型	R	R^2<br>R 方	Adj.R^2<br>调整后 R 方	标准估算的错误
1	.281[a]	.079	.076	17.49944

a. 预测变量：(常量), 人均生产总值, 性别, 疫区类型, 年龄组, 控制程度, 人均可支配收入

模型摘要

模型	R	R 方	调整后 R 方	标准估算的错误
1	.208[a]	.043	.040	17.39738

a. 预测变量：(常量), 人均生产总值, 性别, 疫区类型, 年龄组, 控制程度, 人均可支配收入

模型摘要

模型	R	R 方	调整后 R 方	标准估算的错误
1	.298[a]	.089	.086	15.36745

a. 预测变量：(常量), 人均生产总值, 性别, 疫区类型, 年龄组, 控制程度, 人均可支配收入

模型摘要

模型	R	R 方	调整后 R 方	标准估算的错误
1	.253[a]	.064	.061	15.73478

a. 预测变量：(常量), 人均生产总值, 性别, 疫区类型, 年龄组, 控制程度, 人均可支配收入

Conclusions

Reviewer #2:

Conclusions supported by data: The opening of the Discussion presents the ESS finding among AS patients as one of three "main findings," giving it unwarranted prominence given that it is statistically significant only in one group, runs in the opposite direction in SLF, and is acknowledged elsewhere as potentially confounded. It should be downgraded. Additionally, the Discussion summary paragraph states that "regions with low P-GDP frequently lacked accessible healthcare resources, leading to delayed interventions" — this reintroduces causal language that was explicitly corrected earlier in the same Discussion in response to previous review comments.

Response:

We sincerely appreciate the experts' corrections. We completely agree that presenting the discovery of ESS (Elimination State) as one of the three major findings indeed gives it an undue prominence. This finding showed the opposite direction and was statistically unstable in the SLF group, and we ourselves have acknowledged the potential for confounding factors. Therefore, we have revised the paragraph at the beginning of the Discussion:

- The phrase 'three main findings' was removed, and the original third point (findings related to ESS) was taken out of the main findings and no longer presented as a parallel main conclusion.
- The paragraph was reorganized to retain only two key findings, with ESS-related observations presented as an additional exploratory result requiring cautious interpretation to reduce its emphasis (a discussion section has been added).

At the same time, regarding the question you raised, "regions with low P-GDP frequently lacked accessible healthcare resources, leading to delayed interventions"， we have revised the sentence in the final summary paragraph of the Discussion. Specifically, we have replaced the causal phrasing "leading to delayed interventions" with a more circumspect and correlational wording: "**which may be associated with delayed interventions**". The revised sentence now reads:

Original text:

"Regions with low P-GDP frequently lacked accessible healthcare resources, **leading to delayed interventions for AS patients and** underscoring structural inequalities that exacerbate disease burden."

Revised:

"*Regions with low P-GDP frequently lacked accessible healthcare resources,* ***which may be associated with delayed interventions for AS patients,***

underscoring structural inequalities that could exacerbate disease burden."

This revision ensures consistency with the cross-sectional, non-causal nature of our study design and aligns with the cautious language used elsewhere in the Discussion (e.g., "observed associations," "should be interpreted as correlational").

We are grateful for the reviewer's meticulous reading and believe the manuscript is now substantially improved. We look forward to your final decision.

Gradient qualification: The conclusion that HRQL follows a gradient AS < SLF < HP is stated without qualification throughout, including in the abstract and author summary. As noted in the Results comments, this is not fully supported for MCS and should be qualified accordingly.

Response:

Thank you very much for your question, which you have mentioned in the 'Results' section, and we have revised it according to your comments.

Editorial and Data Presentation Modifications?

Reviewer #2:

Several language and presentation issues require correction:

"Areas meeting elimination criteria demonstrated co-occurred with HRQL" — broken syntax, requires full rewrite

Response:

Thank you for identifying this unclear phrasing. We have rewritten the sentence for clarity and grammatical correctness. The revised sentence now reads:

"*Areas meeting elimination criteria were positively associated with HRQL (β = 0.114 to 0.109).*"

This change has been made in the **Abstract** (line marked in revised manuscript).

"This study offers provides" (Introduction)

Response:

The duplicate verb has been removed. The corrected sentence is:

"*This study provides a stratified analysis of HRQL distinguishing between AS and SLF in a low-endemic setting...*"
This change appears in the **Introduction** section (the second paragraph of the list preceding the "Results" section).

"case-sectional study approach" (Methods) — not a recognized term

Response:

We agree that "case-sectional" is not standard terminology. We have replaced it with the accurate and widely accepted term:

"*cross-sectional design*"

This correction has been made in the **Methods** section (subsection "Participants").

"Enter mothod" (Methods)

Response:

The typo has been corrected to:

"*Enter method*"

This change is in the **Statistical analysis** subsection of **Methods**.

"Health Commission fo Jiangxi Provincial" (Methods)

Response:

The phrase has been corrected to:

"*Health Commission of Jiangxi Province*"

This appears in the **Methods** section under "Endemic status of schistosomiasis (ESS)", subsection "Operationalization in this study".

"were significantly association with" / "MCS scores were association by" / "Gender disparities further association mental health outcomes" (Results/Discussion) — the same grammatical error appears three times

Response:

We have corrected all three instances:

- In **Results** (Analysis of factors affecting HRQL in schistosomiasis patients):

Original: "were significantly association with" → Corrected: "*were significantly associated with*"

Original: "MCS scores were association by gender" → Corrected: "*MCS scores were associated with gender*"

- In **Discussion** (paragraph on gender disparities):

Original: "Gender disparities further association mental health outcomes" →

Corrected: “*Gender disparities further associated mental health outcomes*”
All corrections have been made in the respective sections.

"This study conducted a stratify and analyze" (Conclusion)

Response:

The incorrect verb form has been revised. The sentence now reads:
“*This study conducted a stratified analysis of HRQL differences between AS and SLF patients in a low-prevalence schistosomiasis setting in Jiangxi Province, China.*”
This change is in the **Conclusion** section.

"strategies references for alleviate" (Author summary)

Response:

The phrasing has been corrected to standard English:
“...provides stage-specific, equity-focused strategies to alleviate the enduring burden of schistosomiasis.”
This change appears in the **Author Summary** section.

We have carefully proofread the entire manuscript to ensure no similar issues remain. All changes are marked using Microsoft Word’s “Track Changes” function for your convenience. We believe the manuscript now meets the journal’s language standards and respectfully submit it for final consideration.
Thank you again for your constructive feedback and support throughout the review process.

Terminology consistency:

"matched case-control design" reappears in the Limitations section despite being corrected elsewhere — should be "matched cross-sectional design" throughout

Response:

We apologize for the oversight. The term “matched case-control design” has been replaced with “*matched cross-sectional design*” in the Limitations section. The manuscript now uses consistent terminology throughout. No other instances of the incorrect term remain.

We appreciate your thoroughness and hope the revised manuscript meets the journal’s standards.

Introduction:

"Although pathogen transmission risk persists objectively, population-level infection rates have markedly declined" — contradictory and should be clarified

Response:

We agree that the original wording could be misleading. The intended meaning is that while the potential for pathogen transmission (e.g., presence of infected snails or contaminated water bodies) still exists in certain endemic foci, the actual infection rate among the human population has decreased substantially due to effective control measures. To avoid contradiction, we have revised the sentence to clearly distinguish between “transmission risk as an ecological condition” and “infection rates as an epidemiological outcome.”

Revised sentence:

"*Although the ecological conditions supporting pathogen transmission (e.g., presence of infected snails or contaminated water bodies) still exist in some areas, the effective implementation of control measures has led to a marked decline in population-level infection rates.* "

"These gaps hinder the development of targeted interventions for residual infections"

Response:

We appreciate the reviewer’s request for clarification. The term “residual infections” here refers to chronic, long-standing schistosomiasis cases (including advanced schistosomiasis and schistosomiasis-induced liver fibrosis) that persist even after transmission has been largely interrupted. These patients are often overlooked because routine surveillance focuses on new infections. We have revised the sentence to explicitly define “residual infections” in this context.

Revised sentence:

"*These gaps hinder the development of targeted interventions for residual infections—i.e., chronic, long-standing schistosomiasis cases (including AS and SLF) that persist even after transmission has been largely interrupted—because such patients are often excluded from routine surveillance focused on new infections.*"

"we demonstrated the interplay between structural inequalities and health outcomes" — "demonstrated" overstates a cross-sectional associational finding

Response:

We fully agree with the reviewer’s concern. Given the cross-sectional design of

our study, we cannot demonstrate causal relationships or definitive “interplay.” We have therefore replaced “demonstrated” with more cautious and accurate wording: “identified associations suggesting.”

Original text:

"we **demonstrated the** interplay between structural inequalities and health outcomes"

Revised:

"*we **identified associations suggesting an** interplay between structural inequalities and health outcomes*"
