# Supplementary material for: Assessing Health-Related Quality of life and socioeconomic impacts in low-endemic schistosomiasis: A study from Rural China
Source: PLoS Negl Trop Dis. 2026 Jul 16;20(7):e0014544. doi: 10.1371/journal.pntd.0014544 (PMC13374899; doi:10.1371/journal.pntd.0014544)
Supplement: Peer review history

# Peer review: Decision Letter 0

**DOI:** 10.1371/journal.pntd.0014544.r001

**Authors:** Dileepa Ediriweera, Wei Wang

19 Jan 2026

PNTD-D-25-01367

Assessing Health-Related Quality of Life and Socioeconomic Impacts in Low-Endemic Schistosomiasis: A Study from Rural China

PLOS Neglected Tropical Diseases

Dear Dr. Hu,

Thank you for submitting your manuscript to PLOS Neglected Tropical Diseases. After careful consideration, we feel that it has merit but does not fully meet PLOS Neglected Tropical Diseases's publication criteria as it currently stands. Therefore, we invite you to submit a revised version of the manuscript that addresses the points raised during the review process.

Please submit your revised manuscript within by Mar 20 2026 11:59PM. If you will need more time than this to complete your revisions, please reply to this message or contact the journal office at plosntds@plos.org. When you're ready to submit your revision, log on to https://www.editorialmanager.com/pntd/ and select the 'Submissions Needing Revision' folder to locate your manuscript file.

Please include the following items when submitting your revised manuscript:

* A letter that responds to each point raised by the editor and reviewer(s). You should upload this letter as a separate file labeled 'Response to Reviewers'. This file does not need to include responses to any formatting updates and technical items listed in the 'Journal Requirements' section below.

* A marked-up copy of your manuscript that highlights changes made to the original version. You should upload this as a separate file labeled 'Revised Manuscript with Track Changes'.

* An unmarked version of your revised paper without tracked changes. You should upload this as a separate file labeled 'Manuscript'.

If you would like to make changes to your financial disclosure, competing interests statement, or data availability statement, please make these updates within the submission form at the time of resubmission. Guidelines for resubmitting your figure files are available below the reviewer comments at the end of this letter.

We look forward to receiving your revised manuscript.

Kind regards,

Wei Wang

Academic Editor

PLOS Neglected Tropical Diseases

Shaden Kamhawi

co-Editor-in-Chief

PLOS Neglected Tropical Diseases

orcid.org/0000-0003-4304-636XX

Paul Brindley

co-Editor-in-Chief

PLOS Neglected Tropical Diseases

orcid.org/0000-0003-1765-0002

**Journal Requirements:**

If the reviewer comments include a recommendation to cite specific previously published works, please review and evaluate these publications to determine whether they are relevant and should be cited. There is no requirement to cite these works unless the editor has indicated otherwise.

1) Please upload all main figures as separate Figure files in .tif or .eps format. For more information about how to convert and format your figure files please see our guidelines:

https://journals.plos.org/plosntds/s/figures

**Reviewers' Comments:**

Reviewer's Responses to Questions

**Key Review Criteria Required for Acceptance?**

As you describe the new analyses required for acceptance, please consider the following:

**Methods**

-Are the objectives of the study clearly articulated with a clear testable hypothesis stated?

-Is the study design appropriate to address the stated objectives?

-Is the population clearly described and appropriate for the hypothesis being tested?

-Is the sample size sufficient to ensure adequate power to address the hypothesis being tested?

-Were correct statistical analysis used to support conclusions?

-Are there concerns about ethical or regulatory requirements being met?

Reviewer #1: The objectives are clearly stated and the study design is appropriate.

Reviewer #2: The study objective is clearly stated, but it is framed broadly and not articulated as a prespecified, testable hypothesis. Moreover, terms such as “influencing factors,” “determinants,” or references to “risk/protective factors” imply causality that is not supported by the cross-sectional design. The design is appropriate for describing and comparing HRQL across disease stages (AS, SLF, HP) and for assessing associations with covariates, but it cannot substantiate causal interpretations or mechanistic claims.

The study population is described, yet key aspects remain insufficiently clear for appraisal of internal and external validity—particularly the sampling strategy, the administrative/unit level of contextual variables (P-DIRH, P-GDP, ESS, TSE), and the implications of the equal group allocation. This design supports between-group comparisons, but it limits inference about population-level HRQL distributions or average HRQL in the underlying community.

The sample size is large and likely adequate to detect between-group differences; however, no formal sample size or power calculation is provided, and power for analyses involving area-level predictors may depend on the number of geographic units represented.

Statistical analyses are partly appropriate: summarising SF-36 scores with medians and IQRs is reasonable given non-normality. Nonetheless, the use of the median test is nonstandard and low power compared with Kruskal–Wallis tests with appropriate post-hoc procedures. The manuscript reports “adjusted P” values without specifying the multiple-comparison correction method. The regression approach raises additional concerns: linear regression on bounded 0–100 outcomes can be acceptable, but model diagnostics and robustness checks are not reported; Standardized β coefficients should not be interpreted as ‘unit changes’, they represent changes in the outcome (in SD units) per 1 SD change in the predictor and are particularly hard to interpret for binary predictors, where the SD depends on category prevalence; backward/stepwise selection is prone to unstable variable selection and inflated significance unless justified and supported by sensitivity analyses. Further, potential clustering is not clearly addressed despite the inclusion of area-level predictors. Finally, SF-36 scoring is not described with sufficient detail for reproducibility (the manuscript suggests a generic 1,2,3… item coding approach without explicitly reporting the SF-36 item-specific recoding/reverse-scoring rules and missing-item handling), and the PCS/MCS computation appears to rely on a simple unweighted average of four domains rather than the standard norm-based, weighted PCS/MCS algorithm.

No major ethical or regulatory concerns are apparent based on the reported procedures.

Reviewer #3: Clarity of objectives and hypothesis

The study design and population groups are described in detail; however, a clearly articulated study objective and explicit, testable hypothesis are not stated in this section. While it is implied that the authors aim to compare health-related quality of life across populations with advanced schistosomiasis, schistosomiasis-induced liver fibrosis, and healthy individuals, this should be explicitly stated, ideally with a directional hypothesis. Clarifying whether the authors hypothesize graded HRQL impairment across disease severity would strengthen the conceptual framing and guide the interpretation of results.

Appropriateness of study design

The cross-sectional design is appropriate for comparing HRQL outcomes across disease states at a single time point. Given that the study's aim appears to be descriptive and comparative rather than causal, the chosen design is acceptable. However, the limitations inherent to cross-sectional studies, particularly the inability to infer temporality or causation between disease status and HRQL outcomes, should be acknowledged explicitly in the manuscript.

Population description and suitability

The study population is well defined, with clear diagnostic criteria for AS and SLF based on national standards. The matching strategy by county, gender, and age enhances comparability between groups and is a strength. The exclusion of individuals with chronic comorbidities that significantly affect quality of life is appropriate for reducing confounding. However, this may limit generalizability to real-world schistosomiasis populations where comorbidities are common. This trade-off should be discussed.

Sample size and statistical power

The manuscript does not report the total sample size per group in this section, nor does it describe any a priori sample size or power calculation. Without this information, it isn't easy to assess whether the study was adequately powered to detect meaningful differences in HRQL scores or associations in multivariate analyses. The authors should clarify the sample size rationale and discuss potential implications of insufficient power, if applicable.

Statistical analysis

The use of nonparametric methods for non-normally distributed SF-36 scores is appropriate, and the normalization of scores to a 0-100 scale follows standard practice. The description of the PCS and MCS computation is clear.

However, there are some concerns regarding statistical consistency. While group comparisons use nonparametric tests and medians, multivariate linear regression assumes normally distributed residuals and linear relationships. The authors should justify the use of linear regression for PCS and MCS scores, report diagnostic checks, or consider alternative models if assumptions are violated. In addition, backward elimination can increase the risk of overfitting and model instability; justification for this approach, along with the reporting of model diagnostics, would strengthen the analysis.

Ethical and regulatory considerations

Ethical approval is clearly stated, including the approving body and reference number. Informed consent procedures, participants’ right to withdraw, and anonymization of data are adequately described. There are no apparent ethical or regulatory concerns based on the information provided.

**********

**Results**

-Does the analysis presented match the analysis plan?

-Are the results clearly and completely presented?

-Are the figures (Tables, Images) of sufficient quality for clarity?

Reviewer #1: Yes

Reviewer #2: The Results section is generally well organised and reports clear between-group differences in HRQL; however, several aspects require correction. First, a standard baseline characteristics table comparing AS, SLF, and HP is needed to document group comparability, provide clear denominators, and avoid relying on narrative summaries alone. Interpretation should be aligned strictly with the statistical findings: the stated “progressive decline/gradient” is well supported for PCS, but is less consistently supported for MCS (SLF vs HP is not clearly reported as significant), so conclusions should be qualified accordingly. The use of the median test is nonstandard and low power; please justify it or report Kruskal–Wallis tests with an appropriate post-hoc procedure, and specify the multiplicity correction underlying “adjusted P” values. Domain-level reporting also needs improvement: χ² statistics are presented without mapping to specific domains, and RP/RE exhibit pronounced ceiling effects (median 100 across groups), for which median (IQR) alone is insufficient—consider reporting Q1/Q3 explicitly and/or proportions at ceiling/floor.

Regression results should be framed as associations rather than causal effects: current language over-claims (“determinants,” “risk factor,” “improved HRQL,” “protective factors”). Several predictors (P-GDP, P-DIRH, ESS, TSE) are county/area-level contextual measures; interpreting them as individual-level exposures (e.g., “poverty” at household level, or “elimination improves HRQL”) risks ecological fallacy and should be avoided. Relatedly, interpretability of socioeconomic effects is limited because P-DIRH and P-GDP are dichotomized (high/low); therefore statements implying “unit reductions” are incorrect, and effect estimates should be expressed as differences between categories. The positive association between ESS “elimination” and PCS/MCS in the AS group warrants cautious interpretation and exploration (e.g., contextual confounding, or model instability), rather than being presented as evidence of program effect.

Finally, several reporting issues require correction: “SLP” in the table should be “SLF,” p-values should follow standard conventions (e.g., report P < 0.001 rather than P < 0.0001), and regression tables should include sample size (N) per model, model fit (R²/adjusted R²), and—ideally—95% confidence intervals for coefficients.

Reviewer #3: RESULTS

Overall, the analyses presented align with the statistical methods described earlier. Group comparisons of PCS, MCS, and SF-36 dimension scores use nonparametric summaries and median-based tests, as planned. The use of multivariate linear regression to identify factors associated with PCS and MCS is consistent with the stated approach.

However, some elements of the analysis plan are not fully reflected in the results. The manuscript does not clearly describe how multiple pairwise comparisons were adjusted, despite reporting adjusted P values. The specific adjustment method should be stated and consistently applied. In addition, while backward regression is mentioned, there is no reporting of model diagnostics, goodness-of-fit measures, or checks of regression assumptions, which limits assessment of the robustness of the findings.

Clarity and completeness of results presentation

The Results section is generally well structured, progressing logically from sample characteristics to overall HRQL outcomes, dimension-level analyses, and multivariate modeling. Key findings are clearly stated, and the reporting of medians and IQRs is appropriate for non-normally distributed data.

That said, some results would benefit from greater completeness and precision. The sample size per group is not explicitly reported in the Results, despite being critical for interpreting effect estimates and regression outputs. In the regression analyses, only standardized beta coefficients and P values are presented; confidence intervals and measures of explained variance (such as R²) are not reported, which limits interpretability and comparison across models. Additionally, the text occasionally strays toward interpretation (for example, statements implying improvement due to the elimination status), which would be more suitable for the Discussion section.

Quality and clarity of figures and tables

The figures and tables appear conceptually appropriate for the data presented. Figures 1 and 2 are used to visualize distributions and median differences across groups, which support the nonparametric analytical approach. The figure legends are generally clear and define group abbreviations and SF-36 dimensions.

However, the quality of the presentation cannot be fully assessed without viewing the actual figures. Based on the text, it would be important that figures clearly display medians, IQRs, and group sizes, and that axes are labeled consistently with score ranges (0-100). In Figure 2, the reporting of multiple chi-square values in the text is unclear and may benefit from a clearer mapping between each statistic and the corresponding SF-36 dimension.

Table 1 presents key regression results, but has some clarity issues. The table heading refers to “SLP” instead of “SLF,” which may confuse readers. Variable coding and reference categories are not explicitly stated in the table, and the inclusion of non-significant predictors alongside significant ones without a clear rationale may reduce readability. Adding confidence intervals and clarifying reference groups would improve the table’s usefulness.

**********

**Conclusions**

-Are the conclusions supported by the data presented?

-Are the limitations of analysis clearly described?

-Do the authors discuss how these data can be helpful to advance our understanding of the topic under study?

-Is public health relevance addressed?

Reviewer #1: Yes

Reviewer #2: The manuscript addresses a question of clear public health relevance in a post-control context, highlighting that morbidity and quality-of-life impacts can persist even as transmission declines. The effort to incorporate socioeconomic and geographic context is commendable and aligns with the need to understand social determinants of health when designing equitable elimination-phase strategies. In addition, the emphasis on mental health adds value, as psychosocial burden is often under-recognized in neglected tropical disease programs and may be especially consequential in resource-constrained rural settings.

However, the Discussion frequently overreaches the evidence base: causal language and framing (“determinants,” “risk/protective factors,” “improved HRQL,” “progressive impact,” “actionable evidence,” “mechanisms”) should be replaced with associational language consistent with a cross-sectional design, and the narrative should be aligned strictly with the reported results (the stage gradient is clearly supported for PCS, whereas MCS differences—particularly SLF vs HP—appear less consistent and should be qualified). Several contextual predictors (P-GDP, P-DIRH, ESS, TSE) are area-level measures; the Discussion should avoid individual-level interpretations (household poverty; elimination causing HRQL improvements) and acknowledge potential ecological inference and residual confounding, especially given inconsistent ESS directions across groups.

The Conclusions are currently too strong for the design and analyses: claims of being the “first,” of elucidating “mechanisms,” and of demonstrating that current control strategies “underestimate” HRQL burdens or that specific interventions “can mitigate enduring damage” should be moderated and framed as implications/hypotheses rather than findings. A more defensible conclusion would emphasize stage-stratified HRQL differences and associated factors, and recommend longitudinal and multi-site studies to evaluate causal pathways and interventions.

The Limitations section should be strengthened materially. Beyond general self-report concerns, it should avoid framing SF-36 limitations primarily as “recall bias” and instead emphasize reporting/response bias (e.g., social desirability, interviewer effects, and differential interpretation of items across groups, particularly in older and rural populations). It should also explicitly address: (i) cross-sectional inference (no temporality), (ii) representativeness and potential selection bias, (iii) the contextual/area-level nature and dichotomisation of socioeconomic indicators (risk of ecological fallacy and information loss), (iv) stepwise/backward model selection and multiple testing, (v) potential clustering by geographic unit, and (vi) SF-36 scoring choices (PCS/MCS computation method) and ceiling effects in RP/RE. The proposed future directions (longitudinal, comparative, mixed-methods) are appropriate, but should be explicitly linked to these specific limitations.

Reviewer #3: Conclusion

1. Are the conclusions supported by the data presented?

The Conclusion accurately reflects the core findings of the study, particularly the stratified HRQL differences between AS and SLF patients and the persistent burden observed in a low-prevalence setting. The emphasis on underrecognized HRQL impairment is well supported by the documented reductions in SF-36 physical and mental component scores, as well as the socioeconomic gradients identified in the regression analyses.

However, as with the Discussion, some caution is warranted in phrasing. Statements suggesting that combined HRQL-focused interventions can mitigate long-term damage should be framed more explicitly as implications derived from observed associations rather than demonstrated outcomes of this study. Clarifying this would maintain methodological consistency with the cross-sectional design.

2. Are the limitations of analysis acknowledged or reflected in the Conclusion?

The Conclusion does not explicitly restate limitations, which is acceptable and common for this section. However, it appropriately avoids overgeneralization by anchoring claims to the Jiangxi Province context and by calling for future efforts rather than asserting definitive solutions.

One potential improvement would be a brief qualifier acknowledging that the findings, while informative, are context-specific. This would further align the Conclusion with the carefully articulated limitations presented earlier in the manuscript.

3. Do the authors explain how the findings advance understanding of the topic?

Yes. The Conclusion clearly articulates the study’s contribution by highlighting its novel stratification of AS and SLF patients in a low-prevalence setting and its integration of economic and epidemiological data. This reinforces the manuscript’s central value: shifting the narrative from transmission metrics alone to long-term morbidity and quality-of-life outcomes.

The framing effectively positions the study as filling a gap between disease elimination targets and patient-centered outcomes, which is a meaningful conceptual advance for schistosomiasis research and for neglected tropical diseases more broadly.

4. Is public health relevance clearly articulated?

Yes, and this is one of the strongest aspects of the Conclusion. The linkage to WHO elimination goals and the call for multidimensional strategies align well with current global health priorities. The emphasis on equitable healthcare access, mental health support, and multisectoral collaboration resonates strongly with the PLOS NTDs readership.

The Conclusion successfully reframes schistosomiasis control as not only a parasitological or epidemiological challenge, but also a long-term public health and social equity issue. This framing strengthens the manuscript’s policy relevance and translational value.

**********

**Editorial and Data Presentation Modifications?**

Use this section for editorial suggestions as well as relatively minor modifications of existing data that would enhance clarity. If the only modifications needed are minor and/or editorial, you may wish to recommend “Minor Revision” or “Accept”.

Reviewer #1: No suggestions for modifications.

Reviewer #2: • Add a baseline characteristics table.

• Clarify administrative units and terminology.

• Improve SF-36 reporting format. Report scores consistently as median (Q1, Q3) rather than median (IQR width) alone, and ensure domain labels are consistent (e.g., “social functioning” for SF).

• Correct and standardize statistical reporting.

- Use conventional p-value formatting (e.g., P < 0.001 rather than P < 0.0001).

- When presenting global tests across domains, label test statistics by domain (currently χ² values are listed without mapping).

- Specify the multiple-comparison adjustment underlying “adjusted P”.

• Revise regression table presentation.

- Correct typos/labels (e.g., “SLP” → “SLF”).

- Add N per model, R²/adjusted R², and 95% confidence intervals for coefficients;

- Avoid interpreting standardized β as “unit change”

• Improve the radar plot readability (Fig. 2). With filled polygons, overlapping colors blend and obscure group differences. It would be clearer to (i) plot the largest polygon first and the smallest last , and/or (ii) reduce fill opacity substantially and emphasize distinct outlines/markers. Alternatively, consider showing lines only (no fill).

• Wording and style edits (examples). Several phrases are awkward or imprecise and should be revised for clarity and methodological accuracy, e.g.:

- “incidence of presenting infections” (unclear; consider “active infection,” “infection burden” as appropriate)

Reviewer #3: (No Response)

**********

**Summary and General Comments**

Use this section to provide overall comments, discuss strengths/weaknesses of the study, novelty, significance, general execution and scholarship. You may also include additional comments for the author, including concerns about dual publication, research ethics, or publication ethics. If requesting major revision, please articulate the new experiments that are needed.

Reviewer #1: The study compares health-related quality of life (HRQL) in advanced schistosomiasis (AS) and schistosomiasis-induced liver fibrosis (SLF) patients in low-prevalence schistosomiasis in Jiangxi, China, using economic and epidemiological data to reveal underestimated HRQL impairments. It recommends combining disease control with HRQL-focused interventions, like mental health and economic support, to align with WHO elimination goals. It also emphasizes that equitable healthcare, rehabilitation, and multisectoral collaboration are key to comprehensive recovery. The study's limitations are well stated, and its recommendations for health services and for future research are well justified.

Reviewer #2: This manuscript addresses a question of clear public health relevance: how health-related quality of life (HRQL) differs across schistosomiasis disease stages in a post-control/low-transmission setting. The stage-stratified comparison (AS vs SLF vs healthy comparators) is clinically meaningful and, together with the attempt to incorporate socioeconomic and geographic context, aligns with current priorities to integrate social determinants into elimination-phase strategies. The attention to mental health dimensions is also valuable, as psychosocial burden is often under-recognized in NTD programs and may be particularly consequential in resource-constrained rural settings.

At the same time, substantial revisions are needed to improve methodological transparency, interpretability, and alignment between analyses and conclusions. Key elements of the sampling strategy and population comparability are insufficiently described (particularly selection of the healthy group and the implications of the equal group allocation), limiting assessment of representativeness and potential selection bias. The SF-36 scoring approach is not described with enough detail for reproducibility (SF-36 version, item-specific recoding/reverse scoring and missing-item handling are not specified), and the manuscript’s PCS/MCS computation appears to rely on simple unweighted domain averaging rather than standard norm-based summary scoring; this must be clarified, appropriately labelled, and justified to avoid misleading comparability claims. The statistical approach also requires strengthening: the “median test” is nonstandard/low power, the multiplicity correction underlying “adjusted P” values is not stated, and regression modelling relies on backward selection without adequate justification or sensitivity analyses. Interpretation of standardized β coefficients is problematic (especially for binary predictors), and results involving area-level indicators (P-GDP/P-DIRH/ESS/TSE) should be framed explicitly as contextual associations to avoid ecological inference.

The Discussion and Conclusions further overstate what can be inferred from a cross-sectional design, frequently using causal language (“determinants,” “risk/protective factors,” “improved HRQL,” “mechanisms”) and at points extending beyond the reported results. These sections should be moderated to associational language, and supported by a strengthened limitations discussion that explicitly addresses cross-sectional inference, selection/representativeness, contextual variable interpretation, dichotomisation of predictors, multiple testing, potential clustering by geographic unit, and ceiling effects in some SF-36 domains.

Overall, the topic is important and the dataset appears substantial, but the manuscript would benefit from major revision. No new experiments are required, but recoding, re-analyses and clearer reporting are needed to support robust interpretation.

Reviewer #3: This manuscript addresses an important and often overlooked aspect of schistosomiasis control by examining health-related quality of life (HRQL) in a low-prevalence setting in China. By stratifying patients into advanced schistosomiasis (AS), schistosomiasis liver fibrosis (SLF), and healthy populations, the study moves beyond conventional transmission-focused metrics and provides a patient-centered assessment of disease burden. The topic is timely and relevant, particularly as endemic countries transition from control toward elimination and face the challenge of managing long-term morbidity.

Strengths of the study include the large sample size, the clear stratification of disease stages, and the integration of socioeconomic and regional economic indicators into HRQL analyses. The use of the validated Chinese SF-36 instrument is appropriate, and the analytical approach is generally sound. The Discussion is well contextualized within both national and global schistosomiasis literature, and the public health implications are clearly articulated in line with the WHO 2030 Roadmap. The manuscript is clearly written, well-referenced, and demonstrates solid scholarship.

In terms of novelty, the study is among the first to systematically compare HRQL between AS and SLF patients in a low-prevalence setting while explicitly incorporating economic context. This adds meaningful insight into the residual burden of schistosomiasis after transmission has been reduced and challenges assumptions that morbidity diminishes in parallel with prevalence.

The significance of the findings lies in their relevance to policy and program design. The results underscore the need to complement transmission control with long-term morbidity management, mental health support, and socioeconomic interventions. These insights are particularly valuable for countries approaching elimination, where post-treatment quality of life is increasingly central to comprehensive disease control.

Weaknesses of the study are largely methodological and appropriately acknowledged by the authors. The cross-sectional design limits causal inference, and reliance on self-reported HRQL data introduces potential recall and reporting bias. The absence of key individual-level socioeconomic variables, such as education and healthcare access, may result in residual confounding. Additionally, the focus on a single province limits generalizability to other ecological and cultural settings within China and beyond.

The overall execution of the study is strong. The methodology is clearly described, the analyses are coherent, and the results are presented logically. Minor issues relate mainly to cautious phrasing of conclusions to avoid implying causality and to further tightening of language in some sections for clarity and conciseness.

No concerns are identified regarding research ethics, dual publication, or publication ethics, provided that ethical approval, informed consent, and data availability are clearly documented in the Methods section. The manuscript appears to adhere to accepted ethical standards for human subjects research.

**********

PLOS authors have the option to publish the peer review history of their article (what does this mean?). If published, this will include your full peer review and any attached files.

If you choose “no”, your identity will remain anonymous but your review may still be made public.

**Do you want your identity to be public for this peer review?**  For information about this choice, including consent withdrawal, please see our Privacy Policy.

Reviewer #1: **Yes:**  Ahmed Adeel

Reviewer #2: **Yes:**  Manfredi Greco

Reviewer #3: No

**Figure resubmission:**

While revising your submission, we strongly recommend that you use PLOS’s NAAS tool (https://ngplosjournals.pagemajik.ai/artanalysis) to test your figure files. NAAS can convert your figure files to the TIFF file type and meet basic requirements (such as print size, resolution), or provide you with a report on issues that do not meet our requirements and that NAAS cannot fix.

After uploading your figures to PLOS’s NAAS tool - https://ngplosjournals.pagemajik.ai/artanalysis, NAAS will process the files provided and display the results in the "Uploaded Files" section of the page as the processing is complete. If the uploaded figures meet our requirements (or NAAS is able to fix the files to meet our requirements), the figure will be marked as "fixed" above. If NAAS is unable to fix the files, a red "failed" label will appear above. When NAAS has confirmed that the figure files meet our requirements, please download the file via the download option, and include these NAAS processed figure files when submitting your revised manuscript.

**Reproducibility:**

To enhance the reproducibility of your results, we recommend that authors of applicable studies deposit laboratory protocols in protocols.io, where a protocol can be assigned its own identifier (DOI) such that it can be cited independently in the future. Additionally, PLOS ONE offers an option to publish peer-reviewed clinical study protocols. Read more information on sharing protocols at https://plos.org/protocols?utm_medium=editorial-email&utm_source=authorletters&utm_campaign=protocols

# Author comment: Author response to Decision Letter 1

**DOI:** 10.1371/journal.pntd.0014544.r002

30 Jan 2026

Attachment Submitted filename: Response to Reviewers.pdf

# Peer review: Decision Letter 1

**DOI:** 10.1371/journal.pntd.0014544.r003

**Authors:** Dileepa Ediriweera, Wei Wang, Dileepa Ediriweera, Wei Wang

2 Apr 2026

PNTD-D-25-01367R1

Assessing Health-Related Quality of Life and Socioeconomic Impacts in Low-Endemic Schistosomiasis: A Study from Rural China

PLOS Neglected Tropical Diseases

Dear Dr. Hu,

Thank you for submitting your manuscript to PLOS Neglected Tropical Diseases. After careful consideration, we feel that it has merit but does not fully meet PLOS Neglected Tropical Diseases's publication criteria as it currently stands. Therefore, we invite you to submit a revised version of the manuscript that addresses the points raised during the review process.

Please submit your revised manuscript by May 02 2026 11:59PM. If you will need more time than this to complete your revisions, please reply to this message or contact the journal office at plosntds@plos.org. When you're ready to submit your revision, log on to https://www.editorialmanager.com/pntd/ and select the 'Submissions Needing Revision' folder to locate your manuscript file.

Please include the following items when submitting your revised manuscript:

* A letter that responds to each point raised by the editor and reviewer(s). You should upload this letter as a separate file labeled 'Response to Reviewers'. This file does not need to include responses to any formatting updates and technical items listed in the 'Journal Requirements' section below.

* A marked-up copy of your manuscript that highlights changes made to the original version. You should upload this as a separate file labeled 'Revised Manuscript with Track Changes'.

* An unmarked version of your revised paper without tracked changes. You should upload this as a separate file labeled 'Manuscript'.

If you would like to make changes to your financial disclosure, competing interests statement, or data availability statement, please make these updates within the submission form at the time of resubmission. Guidelines for resubmitting your figure files are available below the reviewer comments at the end of this letter.

We look forward to receiving your revised manuscript.

Kind regards,

Wei Wang

Academic Editor

PLOS Neglected Tropical Diseases

Dileepa Ediriweera

Section Editor

PLOS Neglected Tropical Diseases

Shaden Kamhawi

co-Editor-in-Chief

PLOS Neglected Tropical Diseases

orcid.org/0000-0003-4304-636XX

Paul Brindley

co-Editor-in-Chief

PLOS Neglected Tropical Diseases

orcid.org/0000-0003-1765-0002

**Reviewers' comments:**

Reviewer's Responses to Questions

**Key Review Criteria Required for Acceptance?**

As you describe the new analyses required for acceptance, please consider the following:

**Methods**

-Are the objectives of the study clearly articulated with a clear testable hypothesis stated?

-Is the study design appropriate to address the stated objectives?

-Is the population clearly described and appropriate for the hypothesis being tested?

-Is the sample size sufficient to ensure adequate power to address the hypothesis being tested?

-Were correct statistical analysis used to support conclusions?

-Are there concerns about ethical or regulatory requirements being met?

Reviewer #1: Yes

Reviewer #2: Objectives/design:

The objective is articulated (stage-stratified HRQL comparison and exploration of associated factors), and the matched 1:1:1 comparative design is broadly appropriate for these descriptive/associational aims.

Wording/clarity:

Several key design sentences remain poorly formulated and should be rewritten for scientific clarity:

- “First, all…counties…that cover AS patients…” (unclear selection logic; population description not fully reproducible)

- “Take the AS group served as the baseline cohort…” (grammatically incorrect; “cohort” is misleading in this cross-sectional matched design)

- “try to choose the same township” is not operational; should be described as a rule (township matching prioritized; explicit fallback strategy; success rate if available).

Sample size estimation:

A power rationale is provided, but the formula corresponds to a two-group mean comparison while the objective is framed as three groups. Authors should state explicitly whether powering was based on the smallest pairwise comparison (and which), or provide a multi-group (ANOVA) justification.

SF-36 reliability paragraph is overly detailed: Long lists of Cronbach’s alpha and test–retest ranges are distracting unless the paper is psychometric; this can be shortened to a single sentence with key citations.

Scoring description (critical, still unresolved): The revised text describes norm-based weighted PCS/MCS, but elsewhere still states PCS/MCS are means of four domains. These cannot both be true.

Contextual variables: The clarification that P-DIRH and P-GDP are county-level contextual variables is appropriate, but currently inserted in a staccato way;

Elimination criteria text is redundant/unclear: The “5 consecutive years after interruption” clause should be streamlined and operationalized (how classification was applied to counties in this dataset and for which time period).

Reviewer #3: The authors have addressed several of the earlier methodological issues.

Specifically:

The study objective is now clearly stated, resolving the prior concern regarding lack of clarity.

An a priori sample size and power calculation has been added, with explicit assumptions and target group sizes. This satisfactorily addresses the previous absence of power justification.

The use of backward elimination in multivariate analysis has been replaced with the Enter method, which improves model stability and reduces the risk of overfitting.

Ethical approval and informed consent procedures remain clearly documented.

However, several issues remain:

1. Study Design Terminology: The manuscript continues to refer to both a cross-sectional design and a case-control approach. Based on the description provided, the study is best characterized as a matched cross-sectional comparative study. Consistent terminology would improve methodological clarity.

2. Handling of Matching in Analysis: While matching by age, gender, and residence is described in detail, it remains unclear how this was accounted for analytically. The authors should clarify whether matching was used solely to enhance comparability or incorporated into the statistical analysis.

3. PCS/MCS Computation: There is an internal inconsistency in the description of composite score calculation. The manuscript states that PCS and MCS are weighted summaries derived using norm-based coefficients, but later describes them as simple means of the four component dimensions. This should be clarified to ensure reproducibility.

4. Regression Assumptions: Although linear regression is used for PCS and MCS outcomes, the manuscript does not report whether regression assumptions (e.g., normality of residuals, homoscedasticity, multicollinearity) were assessed. Brief clarification would strengthen the statistical rigor.

Overall, the major concerns raised in the initial review have been addressed, particularly regarding sample size justification and modeling strategy. The remaining issues relate primarily to clarification and internal consistency rather than fundamental design flaws. With these revisions, the methodological section would meet expected standards for transparency and rigor.

**********

**Results**

-Does the analysis presented match the analysis plan?

-Are the results clearly and completely presented?

-Are the figures (Tables, Images) of sufficient quality for clarity?

Reviewer #1: Yes

Reviewer #2: The Results are generally well organised and clearer than the previous version. The switch to Kruskal–Wallis testing with Bonferroni-adjusted post-hoc comparisons, reporting M(Q1, Q3), and explicitly mapping global test statistics to specific SF-36 domains are positive and responsive changes.

Reviewer #3: The authors have substantially improved reporting and transparency. Key updates include:

Sample size: Total participants and group-specific sizes are now clearly reported.

Multiple comparisons: Adjusted P values are clearly linked to pairwise tests, with Bonferroni correction specified.

Regression analyses: Backward elimination has been replaced with enter regression. Standardized β coefficients, 95% confidence intervals, and R²/adjusted R² values are now reported, improving interpretability and methodological rigor.

Figures and tables: Typographical errors corrected, and SF-36 dimension statistics are clearly mapped to test results. Inclusion of non-significant predictors is now consistent with the modeling approach.

Remaining minor issues include:

1. Regression assumption checks (e.g., residual normality, homoscedasticity, multicollinearity) are still not reported.

2. Reference categories for regression variables are not explicitly stated in Table 1.

3. Occasional interpretive statements in the Results text could be neutralized.

4. The unusually wide MCS interquartile range in the HP group may require verification.

Overall, the major deficiencies identified previously have been addressed. The remaining points are minor and pertain primarily to clarity rather than analytical validity. The Results section now meets expected standards for transparency and completeness.

**********

**Conclusions**

-Are the conclusions supported by the data presented?

-Are the limitations of analysis clearly described?

-Do the authors discuss how these data can be helpful to advance our understanding of the topic under study?

-Is public health relevance addressed?

Reviewer #1: Yes

Reviewer #2: Conclusions supported by data:

The main take-home—that HRQL differs by disease stage in a low-transmission setting—is supported, particularly for PCS.

Limitations (improved but can be tightened): L

imitations are more comprehensive than in the original version and appropriately acknowledge key constraints of cross-sectional inference and contextual (county-level) predictors. The section would benefit from clearer integration (less “patchwork” insertion).

Contribution to understanding (yes, with focus):

The discussion of stage-stratified HRQL and the attempt to incorporate socioeconomic/geographic context can advance understanding of residual morbidity in post-control settings.

Redundancy with Introduction:

The opening paragraph largely reiterates background already covered in the Introduction; it can be shortened to focus on interpretation of the study’s main findings and contributions.

Off-target instrument discussion:

Justification of instrument choice (SF-36 vs EQ-5D-5L) is more appropriate in the Methods; the example involving rural hypertensive patients is tangential to schistosomiasis and can be removed or replaced with schistosomiasis-specific measurement considerations.

Mechanistic overreach:

Some statements introduce unmeasured mechanisms as if established (e.g., low P-GDP “leading to delayed interventions” due to limited healthcare resources). Since healthcare access and treatment timing were not assessed, these should be reframed explicitly as hypotheses.

Reviewer #3: The revised Discussion and Conclusion sections demonstrate substantial improvements in clarity, interpretation, and contextualization of findings compared to the earlier version.

Interpretation of findings and causal language:

Previous concerns included language implying causation despite the cross-sectional design. In the revised text, the authors explicitly frame all associations as correlational, noting that cross-sectional data do not support causal inference. Statements regarding the positive association between residing in elimination-status areas (ESS) and higher HRQL are now clearly presented as observational and hypothesis-generating rather than causal. This addresses the earlier issue effectively.

Integration of socioeconomic and geographic factors:

Earlier comments highlighted limited discussion of socioeconomic influences on HRQL. The revised Discussion explicitly incorporates P-DIRH, P-GDP, TSE, and ESS variables, describing their relationships with PCS and MCS scores. The text contextualizes these associations within structural inequalities and regional healthcare access, strengthening the interpretive framework.

Stratification by disease stage and HRQL:

The revised manuscript now clearly differentiates HRQL outcomes across AS, SLF, and healthy populations. Dimension-level differences (PF, VT, SF, GH, BP, MH) are described in detail, with pairwise comparisons and trends consistently interpreted. This addresses the prior comment that stage-specific HRQL impacts were underemphasized.

Mental health outcomes:

Previously, mental health was less emphasized relative to physical health. The revised Discussion now thoroughly addresses MCS components, including vitality, social functioning, and mental health dimensions, and highlights gender disparities. These additions provide a more complete multidimensional assessment of HRQL.

Limitations:

Earlier concerns included insufficient discussion of SF-36 limitations, generalizability, and potential biases. The revised text now acknowledges the cross-sectional design, SF-36 algorithm considerations, ceiling effects, matched sampling, exclusion of comorbidities, and risks of ecological fallacy with area-level socioeconomic measures. Potential clustering by geographic units is noted, and the text appropriately cautions against extrapolation beyond the study context. Residual confounding from unmeasured individual-level factors could be emphasized slightly more, but the current revisions substantially strengthen transparency.

Public health relevance:

The revised sections now clearly articulate the study’s relevance to WHO elimination goals, multidimensional intervention strategies, and the importance of integrating mental health and socioeconomic support. This aligns well with global health priorities and addresses the prior comment that public health implications were underdeveloped.

Summary:

The authors have meaningfully addressed most of the prior concerns. The revised Discussion and Conclusion now present a careful, context-sensitive interpretation, clarify the observational nature of associations, emphasize HRQL stratification by disease stage, and integrate socioeconomic and geographic context. Only minor enhancements could include explicit acknowledgment of residual confounding from unmeasured individual-level variables and continued careful framing of intervention implications as associative rather than causal.

Overall, the revisions have improved the scientific clarity, rigor, and interpretability of the Discussion and Conclusion.

**********

**Editorial and Data Presentation Modifications?**

Use this section for editorial suggestions as well as relatively minor modifications of existing data that would enhance clarity. If the only modifications needed are minor and/or editorial, you may wish to recommend “Minor Revision” or “Accept”.

Reviewer #1: No modifications suggested.

Reviewer #2: While several technical revisions were implemented, many changes read as “patchwork” insertions rather than fully integrated edits, resulting in awkward grammar, staccato transitions, and occasional unclear phrasing. I recommend a focused language and flow edit across the manuscript to merge added sentences smoothly, eliminate redundancy, and ensure consistent terminology and scientific phrasing.

Reviewer #3: Accept

**********

**Summary and General Comments**

Use this section to provide overall comments, discuss strengths/weaknesses of the study, novelty, significance, general execution and scholarship. You may also include additional comments for the author, including concerns about dual publication, research ethics, or publication ethics. If requesting major revision, please articulate the new experiments that are needed.

Reviewer #1: I think the responses to the verous suggestions regarding the original submission are adequate.

Reviewer #2: The revision implements several key methodological improvements compared with the prior version. However, a small number of critical issues remain that limit reproducibility and interpretability.

Reviewer #3: The revised manuscript shows substantial improvements across all sections. The Methods are now detailed and transparent, with clear descriptions of the population, matching strategy, sample size justification, and statistical approaches, including nonparametric analyses and multivariable regression. Ethical considerations are well documented. The Results section is more complete, with explicit group sizes, detailed reporting of PCS, MCS, and SF-36 dimension scores, and regression outputs that include confidence intervals and measures of explained variance. Adjustments for multiple comparisons are now stated, addressing previous concerns about statistical clarity. The Discussion and Conclusion have been strengthened to interpret findings appropriately in a cross-sectional context, emphasizing associations rather than causation, integrating socioeconomic and geographic factors, and highlighting both physical and mental health impacts. Limitations are explicitly acknowledged, and public health relevance is clearly articulated in relation to WHO elimination goals and patient-centered interventions. Overall, the manuscript is scientifically rigorous, well-structured, and effectively communicates the significance of HRQL differences across disease stages, making it suitable for publication with only minor technical clarifications remaining.

**********

PLOS authors have the option to publish the peer review history of their article (what does this mean?). If published, this will include your full peer review and any attached files.

If you choose “no”, your identity will remain anonymous but your review may still be made public.

**Do you want your identity to be public for this peer review?**  For information about this choice, including consent withdrawal, please see our Privacy Policy.

Reviewer #1: **Yes:**  Ahmed Adeel

Reviewer #2: **Yes:**  Manfredi Greco

Reviewer #3: No

[NOTE: If reviewer comments were submitted as an attachment file, they will be attached to this email and accessible via the submission site. Please log into your account, locate the manuscript record, and check for the action link "View Attachments". If this link does not appear, there are no attachment files.]

**Figure resubmission:**

While revising your submission, we strongly recommend that you use PLOS’s NAAS tool (https://ngplosjournals.pagemajik.ai/artanalysis) to test your figure files. NAAS can convert your figure files to the TIFF file type and meet basic requirements (such as print size, resolution), or provide you with a report on issues that do not meet our requirements and that NAAS cannot fix.

After uploading your figures to PLOS’s NAAS tool - https://ngplosjournals.pagemajik.ai/artanalysis, NAAS will process the files provided and display the results in the "Uploaded Files" section of the page as the processing is complete. If the uploaded figures meet our requirements (or NAAS is able to fix the files to meet our requirements), the figure will be marked as "fixed" above. If NAAS is unable to fix the files, a red "failed" label will appear above. When NAAS has confirmed that the figure files meet our requirements, please download the file via the download option, and include these NAAS processed figure files when submitting your revised manuscript.

**Reproducibility:**

To enhance the reproducibility of your results, we recommend that authors of applicable studies deposit laboratory protocols in protocols.io, where a protocol can be assigned its own identifier (DOI) such that it can be cited independently in the future. Additionally, PLOS ONE offers an option to publish peer-reviewed clinical study protocols. Read more information on sharing protocols at https://plos.org/protocols?utm_medium=editorial-email&utm_source=authorletters&utm_campaign=protocols

# Author comment: Author response to Decision Letter 2

**DOI:** 10.1371/journal.pntd.0014544.r004

3 Apr 2026

Attachment Submitted filename: Response_to_Reviewers_auresp_2.pdf

# Peer review: Decision Letter 2

**DOI:** 10.1371/journal.pntd.0014544.r005

**Authors:** Dileepa Ediriweera, Wei Wang, Dileepa Ediriweera, Wei Wang, Dileepa Ediriweera, Wei Wang

20 May 2026

PNTD-D-25-01367R2

Assessing Health-Related Quality of Life and Socioeconomic Impacts in Low-Endemic Schistosomiasis: A Study from Rural China

PLOS Neglected Tropical Diseases

Dear Dr. Hu,

Thank you for submitting your manuscript to PLOS Neglected Tropical Diseases. After careful consideration, we feel that it has merit but does not fully meet PLOS Neglected Tropical Diseases's publication criteria as it currently stands. Therefore, we invite you to submit a revised version of the manuscript that addresses the points raised during the review process.

Please submit your revised manuscript by Jun 19 2026 11:59PM. If you will need more time than this to complete your revisions, please reply to this message or contact the journal office at plosntds@plos.org. When you're ready to submit your revision, log on to https://www.editorialmanager.com/pntd/ and select the 'Submissions Needing Revision' folder to locate your manuscript file.

Please include the following items when submitting your revised manuscript:

* A letter that responds to each point raised by the editor and reviewer(s). You should upload this letter as a separate file labeled 'Response to Reviewers'. This file does not need to include responses to any formatting updates and technical items listed in the 'Journal Requirements' section below.

* A marked-up copy of your manuscript that highlights changes made to the original version. You should upload this as a separate file labeled 'Revised Manuscript with Track Changes'.

* An unmarked version of your revised paper without tracked changes. You should upload this as a separate file labeled 'Manuscript'.

If you would like to make changes to your financial disclosure, competing interests statement, or data availability statement, please make these updates within the submission form at the time of resubmission. Guidelines for resubmitting your figure files are available below the reviewer comments at the end of this letter.

We look forward to receiving your revised manuscript.

Kind regards,

Wei Wang

Academic Editor

PLOS Neglected Tropical Diseases

Dileepa Ediriweera

Section Editor

PLOS Neglected Tropical Diseases

Shaden Kamhawi

co-Editor-in-Chief

PLOS Neglected Tropical Diseases

orcid.org/0000-0003-4304-636XX

Paul Brindley

co-Editor-in-Chief

PLOS Neglected Tropical Diseases

orcid.org/0000-0003-1765-0002

**Journal Requirements:**

If the reviewer comments include a recommendation to cite specific previously published works, please review and evaluate these publications to determine whether they are relevant and should be cited. There is no requirement to cite these works unless the editor has indicated otherwise.

1) Your manuscript's sections are not in the correct order.  Please amend to the following order: Abstract, Introduction, Results, Discussion, and Methods

**Reviewers' comments:**

Reviewer's Responses to Questions

**Key Review Criteria Required for Acceptance?**

As you describe the new analyses required for acceptance, please consider the following:

**Methods**

-Are the objectives of the study clearly articulated with a clear testable hypothesis stated?

-Is the study design appropriate to address the stated objectives?

-Is the population clearly described and appropriate for the hypothesis being tested?

-Is the sample size sufficient to ensure adequate power to address the hypothesis being tested?

-Were correct statistical analysis used to support conclusions?

-Are there concerns about ethical or regulatory requirements being met?

Reviewer #2: Study design: Two nearly identical disclaimers about the matched design appear within one page of each other in the Participants section and should be merged into one. The term "case-sectional study approach" is not a recognized methodological term and should be removed. The term "matched case-control design" reappears in the Limitations section, contradicting the corrected terminology used elsewhere.

Sample size: The manuscript states the power calculation was based on AS vs. HP as "the smallest expected pairwise difference" and "most conservative comparison." This is likely incorrect — AS vs. SLF (two disease stages) would be expected to yield the smaller difference compared to AS vs. HP (disease vs. healthy controls), making AS vs. SLF the more conservative basis for power calculation.

Statistical analyses: Table 1 reports N = 844 for some models and N = 884 for others

**********

**Results**

-Does the analysis presented match the analysis plan?

-Are the results clearly and completely presented?

-Are the figures (Tables, Images) of sufficient quality for clarity?

Reviewer #2: Gradient claim: The abstract and Discussion repeatedly describe an unqualified gradient "AS < SLF < HP" for both PCS and MCS. This is not fully supported: while all three pairwise comparisons are significant for PCS, the SLF vs. HP comparison for MCS is never reported as significant.

Table 1 — ESS direction reversal: Elimination status (ESS) is positively associated with HRQL in AS patients (β = +0.122 PCS, +0.110 MCS) but negatively associated in SLF patients (MCS: β = -0.057, P = 0.038). This opposite direction finding is reported in the table but never acknowledged or discussed anywhere in the manuscript.

Fig. 2: The VT dimension is incorrectly labelled "validity" in the figure legend — it should be "vitality."

Table 1 — R² values: The large discrepancy between unadjusted and adjusted R² values (e.g., 0.281 vs. 0.079 for AS-PCS; 0.208 vs. 0.043 for AS-MCS) is unexpected given the sample size (N ~884) and number of predictors. With these parameters the adjustment penalty should be minimal.

**********

**Conclusions**

-Are the conclusions supported by the data presented?

-Are the limitations of analysis clearly described?

-Do the authors discuss how these data can be helpful to advance our understanding of the topic under study?

-Is public health relevance addressed?

Reviewer #2: Conclusions supported by data: The opening of the Discussion presents the ESS finding among AS patients as one of three "main findings," giving it unwarranted prominence given that it is statistically significant only in one group, runs in the opposite direction in SLF, and is acknowledged elsewhere as potentially confounded. It should be downgraded. Additionally, the Discussion summary paragraph states that "regions with low P-GDP frequently lacked accessible healthcare resources, leading to delayed interventions" — this reintroduces causal language that was explicitly corrected earlier in the same Discussion in response to previous review comments.

Gradient qualification: The conclusion that HRQL follows a gradient AS < SLF < HP is stated without qualification throughout, including in the abstract and author summary. As noted in the Results comments, this is not fully supported for MCS and should be qualified accordingly.

**********

**Editorial and Data Presentation Modifications?**

Use this section for editorial suggestions as well as relatively minor modifications of existing data that would enhance clarity. If the only modifications needed are minor and/or editorial, you may wish to recommend “Minor Revision” or “Accept”.

Reviewer #2: Several language and presentation issues require correction:

"Areas meeting elimination criteria demonstrated co-occurred with HRQL" — broken syntax, requires full rewrite

"This study offers provides" (Introduction)

"case-sectional study approach" (Methods) — not a recognized term

"Enter mothod" (Methods)

"Health Commission fo Jiangxi Provincial" (Methods)

"were significantly association with" / "MCS scores were association by" / "Gender disparities further association mental health outcomes" (Results/Discussion) — the same grammatical error appears three times

"This study conducted a stratify and analyze" (Conclusion)

"strategies references for alleviate" (Author summary)

Terminology consistency:

"matched case-control design" reappears in the Limitations section despite being corrected elsewhere — should be "matched cross-sectional design" throughout

Redundancy:

Two near-identical paragraphs disclaiming the matched design's inferential limits appear within one page in the Participants section and should be merged

Introduction:

"Although pathogen transmission risk persists objectively, population-level infection rates have markedly declined" — contradictory and should be clarified

"These gaps hinder the development of targeted interventions for residual infections"

"we demonstrated the interplay between structural inequalities and health outcomes" — "demonstrated" overstates a cross-sectional associational finding

**********

**Summary and General Comments**

Use this section to provide overall comments, discuss strengths/weaknesses of the study, novelty, significance, general execution and scholarship. You may also include additional comments for the author, including concerns about dual publication, research ethics, or publication ethics. If requesting major revision, please articulate the new experiments that are needed.

Reviewer #2: The manuscript has improved substantially across two revision rounds and no new analyses are required. The statistical approach, matching protocol, regression diagnostics, and limitations section are now appropriate.

However, a focused revision is needed before acceptance.

**********

PLOS authors have the option to publish the peer review history of their article (what does this mean?). If published, this will include your full peer review and any attached files.

If you choose “no”, your identity will remain anonymous but your review may still be made public.

**Do you want your identity to be public for this peer review?**  For information about this choice, including consent withdrawal, please see our Privacy Policy.

Reviewer #2: **Yes:**  Manfredi Greco

**Figure resubmission:**

While revising your submission, we strongly recommend that you use PLOS’s NAAS tool (https://ngplosjournals.pagemajik.ai/artanalysis) to test your figure files. NAAS can convert your figure files to the TIFF file type and meet basic requirements (such as print size, resolution), or provide you with a report on issues that do not meet our requirements and that NAAS cannot fix.

After uploading your figures to PLOS’s NAAS tool - https://ngplosjournals.pagemajik.ai/artanalysis, NAAS will process the files provided and display the results in the "Uploaded Files" section of the page as the processing is complete. If the uploaded figures meet our requirements (or NAAS is able to fix the files to meet our requirements), the figure will be marked as "fixed" above. If NAAS is unable to fix the files, a red "failed" label will appear above. When NAAS has confirmed that the figure files meet our requirements, please download the file via the download option, and include these NAAS processed figure files when submitting your revised manuscript.

**Reproducibility:**

To enhance the reproducibility of your results, we recommend that authors of applicable studies deposit laboratory protocols in protocols.io, where a protocol can be assigned its own identifier (DOI) such that it can be cited independently in the future. Additionally, PLOS ONE offers an option to publish peer-reviewed clinical study protocols. Read more information on sharing protocols at https://plos.org/protocols?utm_medium=editorial-email&utm_source=authorletters&utm_campaign=protocols

# Author comment: Author response to Decision Letter 3

**DOI:** 10.1371/journal.pntd.0014544.r006

26 May 2026

Attachment Submitted filename: Response_to_Reviewers_auresp_3.pdf

# Peer review: Decision Letter 3

**DOI:** 10.1371/journal.pntd.0014544.r007

**Authors:** Dileepa Ediriweera, Wei Wang, Dileepa Ediriweera, Wei Wang, Dileepa Ediriweera, Wei Wang, Dileepa Ediriweera, Wei Wang

3 Jul 2026

Dear Prof. Hu,

We are pleased to inform you that your manuscript 'Assessing Health-Related Quality of Life and Socioeconomic Impacts in Low-Endemic Schistosomiasis: A Study from Rural China' has been provisionally accepted for publication in PLOS Neglected Tropical Diseases.

Before your manuscript can be formally accepted you will need to complete some formatting changes, which you will receive in a follow up email. A member of our team will be in touch with a set of requests.

Please note that your manuscript will not be scheduled for publication until you have made the required changes, so a swift response is appreciated.

IMPORTANT: The editorial review process is now complete. PLOS will only permit corrections to spelling, formatting or significant scientific errors from this point onwards. Requests for major changes, or any which affect the scientific understanding of your work, will cause delays to the publication date of your manuscript.

Should you, your institution's press office or the journal office choose to press release your paper, you will automatically be opted out of early publication. We ask that you notify us now if you or your institution is planning to press release the article. All press must be co-ordinated with PLOS.

Thank you again for supporting Open Access publishing; we are looking forward to publishing your work in PLOS Neglected Tropical Diseases.

Best regards,

Wei Wang

Academic Editor

PLOS Neglected Tropical Diseases

Dileepa Ediriweera

Section Editor

PLOS Neglected Tropical Diseases

Shaden Kamhawi

co-Editor-in-Chief

PLOS Neglected Tropical Diseases

orcid.org/0000-0003-4304-636XX

Paul Brindley

co-Editor-in-Chief

PLOS Neglected Tropical Diseases

orcid.org/0000-0003-1765-0002

***********************************************************

p.p1 {margin: 0.0px 0.0px 0.0px 0.0px; line-height: 16.0px; font: 14.0px Arial; color: #323333; -webkit-text-stroke: #323333}span.s1 {font-kerning: none

Reviewer's Responses to Questions

**Key Review Criteria Required for Acceptance?**

As you describe the new analyses required for acceptance, please consider the following:

**Methods**

-Are the objectives of the study clearly articulated with a clear testable hypothesis stated?

-Is the study design appropriate to address the stated objectives?

-Is the population clearly described and appropriate for the hypothesis being tested?

-Is the sample size sufficient to ensure adequate power to address the hypothesis being tested?

-Were correct statistical analysis used to support conclusions?

-Are there concerns about ethical or regulatory requirements being met?

Reviewer #2: The manuscript has been substantially revised across three rounds and no new analyses are required. The following residual issues should be addressed:

The abstract opening ("To describe and compare...") lacks a subject and reads awkwardly; "This study aimed to describe and compare..." would be more appropriate.

Study design: The term "case-sectional design" persists in the Participants section despite being flagged in previous rounds, this is not a recognized methodological term. The matching success rate (91.3% township-level) is reported in the Methods but belongs in the Results under Sample characteristics. The phrase "content of the situation" in the inclusion criteria is unclear and should be replaced.

Statistical analyses: The large discrepancy between R² and adjusted R² in the previous version has been correctly identified as a data entry error and fixed — the corrected values (e.g., AS-PCS: 0.079/0.076) are now internally consistent and plausible. However the models still explain very little variance (R² 4–9%) and this should be acknowledged

**********

**Results**

-Does the analysis presented match the analysis plan?

-Are the results clearly and completely presented?

-Are the figures (Tables, Images) of sufficient quality for clarity?

Reviewer #2: The Results are clearly presented and match the analysis plan. The following issues remain:

Gradient claim: The abstract correctly limits the gradient AS < SLF < HP to PCS. However the author summary still uses "steep decline" without distinguishing PCS from MCS.

Sample characteristics: The sentence "41.29% of participants inhabited marshland/lake regions, whereas 72.06% were from areas meeting transmission interruption criteria" mixes two different classification systems (TSE and ESS) without making this explicit. These should be reported separately with labels.

**********

**Conclusions**

-Are the conclusions supported by the data presented?

-Are the limitations of analysis clearly described?

-Do the authors discuss how these data can be helpful to advance our understanding of the topic under study?

-Is public health relevance addressed?

Reviewer #2: Conclusions supported by data: The two main findings are well supported. The ESS finding has been appropriately downgraded to an exploratory observation.

The Discussion states that both AS and SLF patients in low P-GDP counties had significantly lower PCS scores, but Table 1 shows this association is only significant in the AS group the SLF-PCS P-GDP association is non-significant (P = 0.200).

The cross-sectional caveat ("observed associations should be interpreted as correlational") is placed as the opening sentence of the Discussion, it would read more naturally within the Limitations.

**********

**Editorial and Data Presentation Modifications?**

Use this section for editorial suggestions as well as relatively minor modifications of existing data that would enhance clarity. If the only modifications needed are minor and/or editorial, you may wish to recommend “Minor Revision” or “Accept”.

Reviewer #2: Section order: The current manuscript places Methods after the Discussion.

Abstract: Opening sentence should include a subject ("This study aimed to...").

Introduction: The citation paragraph on Chinese schistosomiasis literature lists multiple studies without a clear organizing argument and should be restructured to convey one explicit point.

Language errors remaining:

"MCS scores were associated by gender" (Results) — should be "associated with"

"Gender disparities further associated mental health outcomes" (Discussion) — grammatically incorrect

"matched case-sectional design" (Limitations)

"content of the situation" (Methods) — unclear, needs rewriting

Discussion structure:

Cross-sectional caveat should be moved from the Discussion opening to the Limitations

**********

**Summary and General Comments**

Use this section to provide overall comments, discuss strengths/weaknesses of the study, novelty, significance, general execution and scholarship. You may also include additional comments for the author, including concerns about dual publication, research ethics, or publication ethics. If requesting major revision, please articulate the new experiments that are needed.

Reviewer #2: This manuscript has undergone three rounds of substantive revision and addresses a relevant question on HRQL across schistosomiasis disease stages in a low-endemic setting. The authors have engaged constructively and most previously raised issues have been resolved.

I recommend acceptance. However, I wish to draw the authors' and editorial office's attention to several points that should be addressed before final publication. The Discussion overstates the P-GDP finding by stating that both AS and SLF patients in low P-GDP counties had significantly lower PCS scores. The manuscript does not currently conform to the standard PLOS NTDs section order, with Methods appearing after the Discussion rather than before the Results. Additionally, several language errors that were explicitly stated as corrected in the response letter remain present in the manuscript text. These points do not warrant a further revision round but should be verified and corrected at proof stage.

**********

PLOS authors have the option to publish the peer review history of their article (what does this mean?). If published, this will include your full peer review and any attached files.

If you choose “no”, your identity will remain anonymous but your review may still be made public.

**Do you want your identity to be public for this peer review?**  For information about this choice, including consent withdrawal, please see our Privacy Policy.

Reviewer #2: **Yes:**  Manfredi Greco

# Editor report: Acceptance letter

**DOI:** 10.1371/journal.pntd.0014544.r008

**Authors:** Dileepa Ediriweera, Wei Wang, Dileepa Ediriweera, Wei Wang, Dileepa Ediriweera, Wei Wang, Dileepa Ediriweera, Wei Wang

Dear Prof. Hu,

We are delighted to inform you that your manuscript, "Assessing Health-Related Quality of Life and Socioeconomic Impacts in Low-Endemic Schistosomiasis: A Study from Rural China," has been formally accepted for publication in PLOS Neglected Tropical Diseases.

We have now passed your article onto the PLOS Production Department who will complete the rest of the publication process. All authors will receive a confirmation email upon publication.

The corresponding author will soon be receiving a typeset proof for review, to ensure errors have not been introduced during production. Please review the PDF proof of your manuscript carefully, as this is the last chance to correct any scientific or type-setting errors. Please note that major changes, or those which affect the scientific understanding of the work, will likely cause delays to the publication date of your manuscript. Note: Proofs for Front Matter articles (Editorial, Viewpoint, Symposium, Review, etc...) are generated on a different schedule and may not be made available as quickly.

Soon after your final files are uploaded, the early version of your manuscript will be published online unless you opted out of this process. The date of the early version will be your article's publication date. The final article will be published to the same URL, and all versions of the paper will be accessible to readers.

For Research Articles, you will receive an invoice from PLOS for your publication fee after your manuscript has reached the completed accept phase. If you receive an email requesting payment before acceptance or for any other service, this may be a phishing scheme. Learn how to identify phishing emails and protect your accounts at https://explore.plos.org/phishing.

Thank you again for supporting open-access publishing; we are looking forward to publishing your work in PLOS Neglected Tropical Diseases.

Best regards,

Shaden Kamhawi

co-Editor-in-Chief

PLOS Neglected Tropical Diseases

Paul Brindley

co-Editor-in-Chief

PLOS Neglected Tropical Diseases
